# Efficacy and safety of rimegepant 75 mg orally disintegrating tablet for the acute treatment of chronic rhinosinusitis in adults: Results from a multicenter, randomized, placebo-controlled, phase 2/3 trial

**DOI:** 10.1371/journal.pone.0342907

**Published:** 2026-03-04

**Authors:** Daniel Franjic, Robert J. Fountaine, Catherine Nalpas, Budhaditya Goswami, Terence Fullerton

**Affiliations:** 1 Pfizer Inc, Groton, Connecticut, United States of America; 2 Pfizer Inc, Paris, France; 3 Pfizer Inc, Chennai, India; Yamagata University Faculty of Medicine: Yamagata Daigaku Igakubu Daigakuin Igakukei Kenkyuka, JAPAN

## Abstract

Calcitonin gene-related peptide (CGRP) activation could play a causal role in the pathophysiology of chronic rhinosinusitis (CRS), a long-term inflammatory disease of the nasal cavity and paranasal sinuses. This study investigated the efficacy and safety of rimegepant 75 mg orally disintegrating tablet, a CGRP receptor antagonist, versus placebo for acute treatment of CRS. This double-blind, randomized, placebo-controlled, phase 2/3 trial enrolled adults with CRS in the United States. Participants were randomized 1:1 to rimegepant or placebo stratified by the presence or absence of nasal polyps. Participants were dispensed a single dose of study drug administered when they experienced a qualifying facial pain/pressure/fullness (Numeric Rating Scale [NRS] ≥6). The primary efficacy endpoint was a change from baseline [CFB] at 2 hours post dose in the intensity of facial pain/pressure/fullness) with baseline NRS score ≥6. Secondary endpoints included a CFB at 2 hours post dose in the NRS score for nasal obstruction/congestion and nasal discharge, and Total Nasal Symptom Score (TNSS); proportion of participants reporting headache pain relief at 2 hours post dose; and proportion of participants using rescue medication within 24 hours post dose. Efficacy outcomes were evaluated using a linear model. Of 261 participants (mean age: 49.3 years) randomized to rimegepant (n = 131) or placebo (n = 130), 96 and 100 had evaluable data for efficacy analyses. No significant treatment differences (P > 0.05) were observed between groups for the primary (CFB at 2 hours post dose in the NRS score for intensity of facial pain/pressure/fullness, least-squares mean difference [95% CI] −0.1 [−0.7, 0.5]) and secondary outcomes. There were no serious safety findings. To our knowledge, this study is the first to explore the use of CGRP receptor antagonists for CRS. Although no treatment differences were identified, the findings could contribute to the design of future clinical trials and better disease understanding.

Trial registration

ClinicalTrials.gov NCT05248997.

## Introduction

Chronic rhinosinusitis (CRS) is an inflammatory disease of the nasal cavity and paranasal sinuses that has a duration of ≥12 weeks [1,2]. CRS can be broadly divided into two phenotypes based on the presence or absence of nasal polyps, with nasal polyposis present in at least approximately 20% of patients [1,3]. CRS is estimated to affect 5–12% of the general population, although when a requirement for clinically documented inflammation is applied the prevalence is 3–6% [4–6]. The pathophysiologic mechanism responsible for the development of CRS is currently unknown but considered to be multifactorial. Factors believed to predispose individuals to developing CRS include those that are environmental (e.g., allergens, microorganisms, pollutants) and systemic (e.g., abnormal ciliary function, cystic fibrosis, and other genetic factors) [3,5,7,8]. Diagnosis of CRS requires the presence of at least two of the four cardinal symptoms (facial pain/pressure/fullness, nasal obstruction/congestion, nasal discharge, and decreased sense of smell), as well as the confirmed presence of inflammation by radiographic imaging or nasal endoscopy [9].

The goal of treatment is to reduce inflammation (pain and swelling in particular), prevent infections, restore mucociliary clearance, and improve sinus drainage [7]. First-line treatment typically involves topical and intranasal steroids, nasal saline irrigation or spray, and antibiotics [2,10]. However, the acute and long-term efficacy of these options is limited [2,10]. More effective treatment options include systemic corticosteroids and long-term use of antibiotics, but these are associated with significant side effects [11,12]. Despite current medical therapies, around 50% of all patients with CRS will eventually undergo endoscopic surgery in order to control their symptoms and most will continue experiencing inadequate relief from symptoms, whereas about one-third will need at least one revision surgery [12,13]. These suggest that an unmet treatment need remains for patients with CRS.

The calcitonin gene-related peptide (CGRP) receptor is located within pain-signaling pathways, intracranial arteries, and the trigeminal ganglion, and its activation might play a causal role in CRS pathophysiology [14–17]. Rimegepant 75 mg orally disintegrating tablet (ODT) is the only small-molecule CGRP receptor antagonist (i.e., gepant) currently approved by the US Food and Drug Administration and the European Medicines Agency for both acute treatment of migraine and prevention of episodic migraine in adults [18,19]. Treatment with a CGRP receptor antagonist is hypothesized to exert multiple downstream effects that may provide benefit in the treatment of CRS, including blockade of neurogenic inflammation, decrease of arterial dilatation, and inhibition of pain-signal transmission. This multicenter, double-blind, randomized, placebo-controlled, phase 2/3 trial (NCT05248997) aimed to evaluate the efficacy and safety of a single dose of rimegepant 75 mg ODT for the treatment of adults with CRS in the United States.

## Methods

### Study design and treatment

The study protocol was approved by independent ethics committees and/or institutional review boards at each study site and was conducted in accordance with the International Conference on Harmonisation Good Clinical Practice guidelines, the Declaration of Helsinki, and all applicable local regulations. All participants provided written informed consent before any study-related procedures were undertaken.

The trial consisted of a screening period of 3–14 days, an acute treatment phase of ≤45 days or until the participant experienced a qualifying facial pain/pressure/fullness with an intensity ≥6 on the Numeric Rating Scale (NRS), and an end-of-treatment visit ≤7 days after dosing with study drug. The participant recruitment period started on February 17, 2022 and ended on January 31, 2024.

Adult participants (male or female ≥18 years old) were eligible to participate if they had a clinical history of CRS for ≥3 months; facial pain/pressure/fullness and ≥1 of the following symptoms: nasal obstruction/congestion, nasal discharge, or decreased sense of smell; nasal inflammation documented by at least one of the following methods: nasal endoscopy, radiographic imaging, or the presence of nasal polyps; and ≥2 episodes of facial pain/pressure/fullness of moderate or severe intensity in the 30 days prior to the screening visit. Participants were excluded if they had a clinical history of diagnosed primary headache disorder, including migraine, or medication overuse headache consistent with the International Classification of Headache Disorders 3rd Edition criteria, recurrent acute sinusitis (defined as ≥4 episodes of acute bacterial rhinosinusitis per year without signs/symptoms of rhinosinusitis between episodes), invasive fungal rhinosinusitis, hypogammaglobulinemia, immunodeficiency syndrome, granulomatous disease, malignant nasal or paranasal tumor, sinus mucocele or odontogenic sinusitis within 1 year prior to the screening visit, nasal or facial surgery within 6 months prior to the screening visit, or nasal or upper respiratory tract infection within 2 weeks prior to the screening visit; diagnosis of acute bacterial/viral rhinosinusitis or symptoms suggestive of an acute exacerbation of CRS within 2 weeks prior to the screening visit, cystic fibrosis, or primary ciliary dyskinesis; ongoing rhinitis medicamentosa; or were receiving aspirin-desensitization or maintenance therapy for aspirin-exacerbated respiratory disease.

Enrolled participants were randomized 1:1 to rimegepant 75 mg ODT or matching placebo, stratified according to the presence or absence of nasal polyps. Participants were dispensed a single dose of study drug and instructed to use it as an outpatient when they experienced facial pain/pressure/fullness with intensity ≥6 on the NRS. Throughout the treatment phase, participants who experienced facial pain/pressure/fullness with NRS intensity <6 were permitted to use acetaminophen (≤1000 mg/day), non-steroidal anti-inflammatory drugs, non-sedating oral antihistamines, oral decongestants, topical nasal decongestants, and topical nasal anticholinergics (up to the daily recommended dose indicated on the drug packaging) to treat symptoms. Participants were allowed to use rescue medications (the same as listed for facial pain/pressure/fullness with NRS intensity <6) 2 hours after dosing and completing their assessments in the electronic diary (eDiary). Exclusionary rescue medications (e.g., opioids, butalbital compounds) were not allowed in this study. Participants were required to maintain the same dose of any medications they were using to treat CRS symptoms from screening throughout the duration of the study, without initiating any new daily medication for CRS. Participants who required systemic corticosteroids and/or antibiotics during the study were discontinued.

### Outcomes and statistical analysis

#### Efficacy endpoints.

The primary efficacy outcome was the change from baseline (CFB) in the intensity of facial pain/pressure/fullness with baseline NRS severity ≥6 at 2 hours after dosing. The NRS is an 11-point scale ranging from 0 (no pain) to 10 (worst pain imaginable). The initial sample and expected effect size calculations are described in **S1 File**. Secondary efficacy outcomes included the CFB in the NRS score for nasal obstruction/congestion, nasal discharge score, and Total Nasal Symptom Score (TNSS) at 2 hours after dosing; proportion of participants reporting headache pain relief at 2 hours after dosing; and proportion of participants using rescue medication within 24 hours post dose. Exploratory efficacy outcomes included the Sino-Nasal Outcome Test-22 (SNOT-22) score and Patient Global Impression of Change (PGI-C) score at 24 hours after dosing.

Participants recorded efficacy data using an eDiary. The severity of three symptoms (facial pain/pressure/fullness, nasal congestion/obstruction, and nasal discharge) based on the NRS were recorded immediately before the use of study drug and at 0.25, 0.5, 0.75, 1, 1.5, 2, 4, 8, and 24 hours post dose. The TNSS was calculated as the sum of the scores recorded for these three symptoms. Participants with moderate or severe headache pain at the time of experiencing facial pain/pressure/fullness with NRS ≥ 6 recorded their pain intensity using a 4-point Likert scale (0 = none; 1 = mild; 2 = moderate; 3 = severe) at the same time points as those used for the NRS scores. The SNOT-22 is a 22-item symptom-based questionnaire that is divided into five domains (rhinologic, extranasal, ear/facial, psychologic, and sleep disturbance) [20]. The score for each item ranges from 0 (no problem) to 5 (problem as bad as it can be), with a total score of 0–110 (lower scores indicate better sinonasal-related quality of life [QoL]) [21]. The PGI-C is another patient-reported outcome measure (PROM) that assesses how an individual’s disease symptoms change over time using a 7-point Likert scale (1 = very much improved; 2 = much improved; 3 = minimally improved; 4 = no change; 5 = minimally worse; 6 = much worse; 7 = very much worse) [22,23].

Primary and secondary endpoints assessing changes from baseline were analyzed using a linear model that included baseline value as a covariate, along with treatment group, stratification factor (presence of nasal polyps: yes or no), scheduled time point, and the time point-by-treatment group interaction as fixed effects. The model did not include random effects, and the G matrix was effectively empty and therefore not used. Repeated measures within participants were modeled using an unstructured covariance matrix (R matrix) to account for within participant error. The model included time points at 0.25, 0.5, 0.75, 1, 1.5, and 2 hours post dose. All analyses were conducted under the assumption of normality within the repeated measures model. Assessment of linear model fit using studentized residuals indicated no substantial deviation from normality for the primary and secondary endpoints. Analyses following categorization of participants according to the presence of nasal polyps excluded this stratification factor in the linear model. All assessments after rescue medication administration were set to missing. Analyses of headache pain relief and the use of rescue medications were stratified by the presence or absence of nasal polyps at randomization using Mantel–Haenszel risk estimation. Analysis of SNOT-22 scores was based on analysis of covariance (ANCOVA) models with baseline score as a covariate and treatment group and stratification factor as fixed effects. For analysis of the PGI-C, odds ratio with Wald 95% confidence interval (CI) was calculated using a cumulative logit model with treatment group and stratification factor as fixed effects. Efficacy outcomes were assessed in the modified intent-to-treat (mITT) analysis set that included randomized participants who took study therapy, had qualifying face pain/pressure/fullness (intensity ≥6 on the NRS) prior to administering treatment, and provided ≥1 post-baseline efficacy data point.

#### Safety endpoints.

Safety was monitored throughout the study and included adverse events (AEs), ECG assessments, vital signs and physical measurements, routine clinical laboratory tests, and the Columbia-Suicide Severity Rating Scale (C-SSRS) [24]. The clinician-administered C-SSRS was completed at the baseline and end-of-treatment visits. Safety outcomes were assessed in the safety analysis set.

## Results

**Fig 1:**
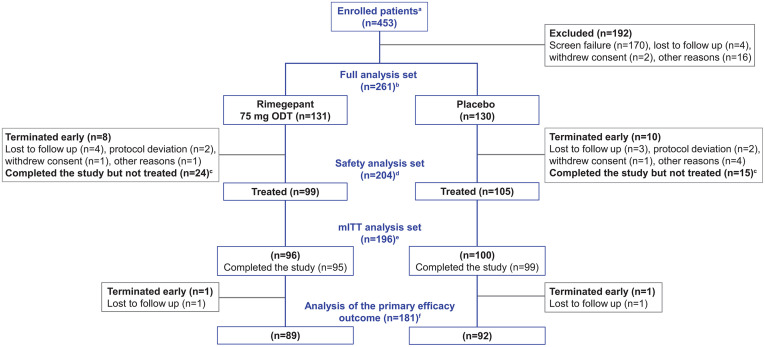
Patient disposition. ^a^ Enrolled patients provided informed consent and were assigned a subject identification number. ^b^ The full analysis set included patients who were randomized (1:1) to receive rimegepant 75 mg ODT or placebo. ^c^ Patients were not treated as they did not have a qualifying face pain/pressure/fullness of intensity ≥6 on the NRS. ^d^ The safety analysis set included randomized patients who took study therapy (rimegepant 75 mg ODT or placebo). ^e^ The mITT analysis set included randomized patients who took study therapy, had face pain/pressure/fullness with an intensity of ≥6 on the NRS before administering treatment, and provided at least one post-baseline efficacy data point. ^f^ The primary outcome was CFB in the NRS score for face pain/pressure/fullness at 2 hours post dose. Abbreviations: CFB, change from baseline; mITT, modified intent-to-treat; NRS, Numeric Rating Scale; ODT, orally disintegrating tablet.

Between February 2022 and April 2024, 453 participants with CRS were enrolled across 28 sites (Fig 1). After undergoing all screening procedures, 261 participants were randomized and comprised the full analysis set (rimegepant [n = 131], placebo [n = 130]). Eighteen participants (rimegepant [n = 8], placebo [n = 10]) terminated the study prematurely and were not treated.

Of the 261 participants, 99 and 105 used rimegepant and placebo, respectively, and were included in the safety analysis set (n = 204), of whom 2 participants did not complete the study (lost to follow-up: rimegepant [n = 1], placebo [n = 1]) (Fig 1). Furthermore, 96 and 100 participants in the rimegepant and placebo groups met criteria for efficacy analyses and were included in the mITT analysis set (n = 196).

Demographic and clinical characteristics were similar in the two treatment arms at baseline (Table 1). In the overall study population (n = 261), the mean (standard deviation [SD]) age of participants was 48.6 (15.3) and 49.9 (15.8) years in the rimegepant and placebo arms, respectively. In both groups, most were female (58.8% vs 52.3%), White (80.9% vs 80.8%), and had CRS without nasal polyps (rimegepant vs placebo: 79.4% vs 80.0%).

**Table 1:** Patient demographics and clinical characteristics at baseline (full analysis set).

	Treatment arm		Total N = 261
	Rimegepant 75 mg ODT n = 131	Placebo n = 130	
Age, mean (SD), y	48.6 (15.3)	49.9 (15.8)	49.3 (15.5)
Sex, n (%)			
Female	77 (58.8)	68 (52.3)	145 (55.6)
Male	54 (41.2)	62 (47.7)	116 (44.4)
Randomization stratum (presence of nasal polyps), n (%)			
No	104 (79.4)	104 (80.0)	208 (79.7)
Yes	27 (20.6)	26 (20.0)	53 (20.3)
Body mass index, mean (SD), kg/m^2^	27.2 (3.77)	26.9 (4.09)	27.1 (3.93)
Race, n (%)			
White	106 (80.9)	105 (80.8)	211 (80.8)
Black or African American	19 (14.5)	17 (13.1)	36 (13.8)
Other^a^	6 (4.6)	8 (6.2)	14 (5.4)

^a^Included patients who were Asian, Native Hawaiian or other Pacific Islander, multiracial, and of other race.

Abbreviations: ODT, orally disintegrating tablet; SD, standard deviation.

### Primary efficacy outcome

**Fig 2:**
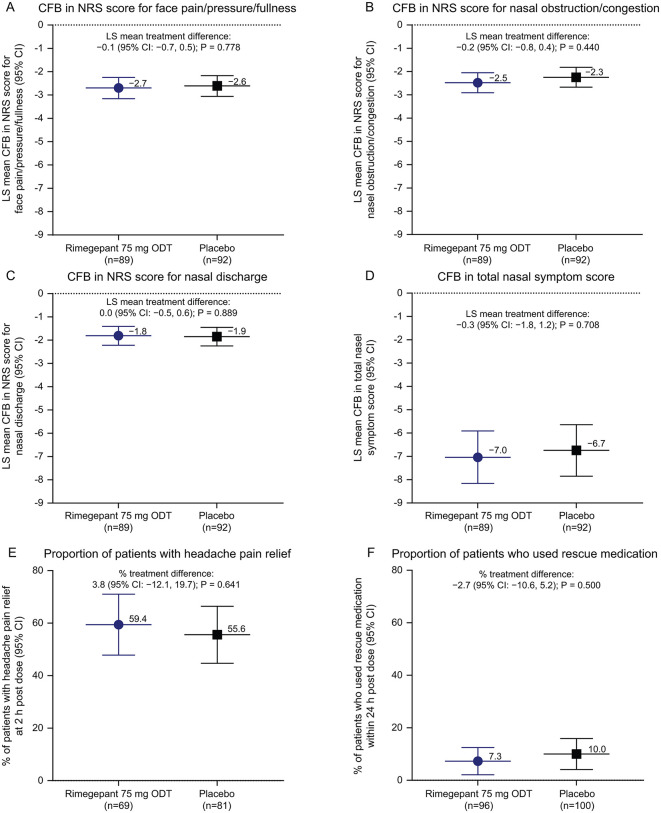
Primary and secondary efficacy endpoints for the overall mITT analysis set. CFB in NRS score for face pain/pressure/fullness **(A)**, nasal obstruction/congestion **(B)**, and nasal discharge **(C)**, total nasal symptom score **(D)**, and the proportion of patients with headache pain relief **(E)** at 2 hours post dose; and the proportion of patients who used rescue medication within 24 hours post dose **(F)**. The nominal P value is shown for the secondary efficacy outcomes (panels **B–F**). Abbreviations: CI, confidence interval; CFB, change from baseline; LS, least-squares; mITT, modified intent-to-treat; NRS, Numeric Rating Scale; ODT, orally disintegrating tablet.

**Fig 3:**
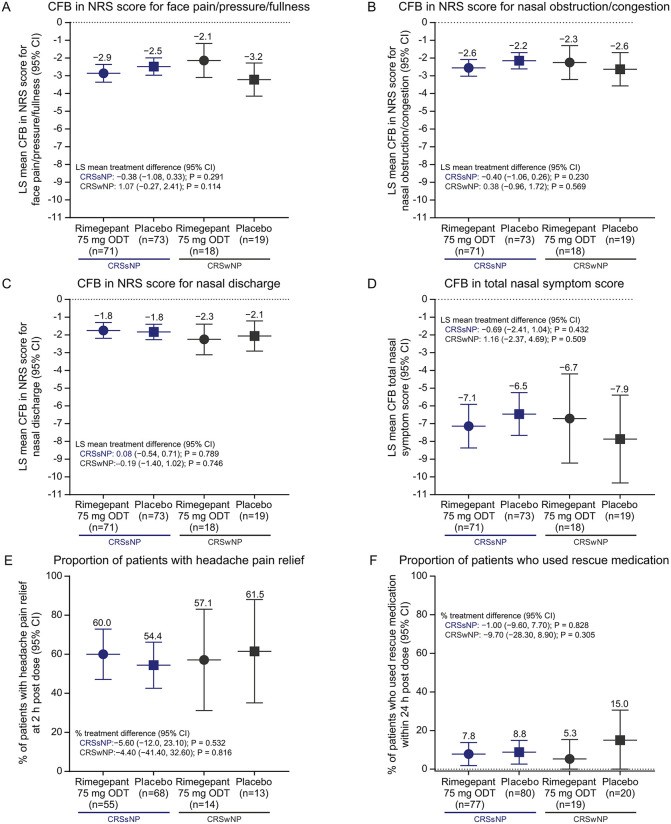
Primary and secondary efficacy endpoints with participants categorized based on the presence/absence of nasal polyps. CFB in NRS score for face pain/pressure/fullness **(A)**, nasal obstruction/congestion **(B)**, and nasal discharge **(C)**, total nasal symptom score **(D)**, and the proportion of patients with headache pain relief **(E)** at 2 hours post dose; and the proportion of patients who used rescue medication within 24 hours post dose **(F)**. The nominal P value is shown for the secondary efficacy outcomes (panels **B–F**). Abbreviations: CI, confidence interval; CFB, change from baseline; CRSsNP, chronic rhinosinusitis without nasal polyp; CRSwNP, chronic rhinosinusitis with nasal polyp; LS, least-squares; mITT, modified intent-to-treat; NRS, Numeric Rating Scale; ODT, orally disintegrating tablet.

The mean observed NRS score for face pain/pressure/fullness at baseline and 2 hours post dose is plotted in S1A Fig. The least-squares (LS) mean CFB (95% CI) was −2.7 (−3.2, −2.3) in the rimegepant and −2.6 (−3.1, −2.2) in the placebo arms (Fig 2A). There was no significant difference (95% CI) between treatment groups in the primary outcome (−0.1 [−0.7, 0.5]; 2-sided P = 0.778), based on the linear model. Moreover, similar results for the primary outcome were obtained when participants were categorized based on the presence or absence of nasal polyps (treatment difference [95% CI] between groups: CRS without nasal polyp [CRSsNP] −0.4 [−1.1, 0.3], 2-sided P = 0.291; CRS with nasal polyp [CRSwNP] 1.1 [−0.3, 2.4], 2-sided P = 0.114) (Fig 3A and S2A Fig).

### Secondary efficacy outcomes

The mean observed NRS score for nasal obstruction/congestion and discharge, and TNSS at baseline and 2 hours post dose is plotted in S1B–S1D Fig. The corresponding LS mean CFB at 2 hours post dose is shown in Fig 2B–D. There were no significant treatment differences (95% CI, nominal P value) between groups at 2 hours post dose in the NRS score for nasal obstruction/congestion (−0.2 [−0.8, 0.4]; P = 0.440), NRS score for nasal discharge (0.0 [−0.5, 0.6]; P = 0.889), and TNSS (−0.3 [−1.8, 1.2]; P = 0.708). Moreover, the proportion of participants who experienced headache pain relief at 2 hours post dose (rimegepant vs placebo, 59.4% vs 55.6%; nominal P = 0.641) and took rescue medication within 24 hours post dose (7.3% vs 10.0%; P = 0.500) were similar between treatment arms (Fig 2E and 2F). Further, results were consistent for all secondary outcomes when participants were categorized based on the presence or absence of nasal polyps including the CFB at 2 hours post dose in the NRS score for nasal obstruction/congestion (treatment difference [95% CI, nominal P value] between groups): (CRSsNP −0.4 [−1.1, 0.3], P = 0.230; CRSwNP, 0.4 [−1.0, 1.7], P = 0.569); nasal discharge (CRSsNP 0.1 [−0.5, 0.7], P = 0.789; CRSwNP, −0.2 [−1.4, 1.0], P = 0.746); TNSS (CRSsNP −0.7 [−2.4, 1.0], P = 0.432; CRSwNP 1.2 [−2.4, 4.7], P = 0.509); proportion of participants achieving headache pain relief at 2 hours post dose (rimegepant vs placebo: CRSsNP 60.0% vs 54.4%, nominal P = 0.532; CRSwNP 57.1% vs 61.5% [−41.4, 32.6], P = 0.816); and the proportion of participants using rescue medication ≤24 hours post dose (rimegepant vs placebo: CRSsNP 7.8% vs 8.8% [−9.6, 7.7], nominal P = 0.828; CRSwNP 5.3% vs 15.0%, [−28.3, 8.9], P = 0.305) (Fig 3B–F, S2B–S2D Fig).

### Exploratory efficacy outcomes

Among all participants in the mITT analysis set, 83 (rimegepant) and 82 (placebo) completed the PGI-C survey at 24 hours post dose. Similar proportions of rimegepant- and placebo-treated participants reported improvement (79.5% vs 75.6%), stability (18.1% vs 20.7%), and worsening (2.4% vs 3.7%) in their CRS symptoms (odds ratio [Wald 95% CI] for improvement, rimegepant vs placebo: 1.3 [0.60, 2.59], Table 2).

**Table 2:** PGI-C at 24 hours post dose (mITT analysis set).

	Treatment arm	
	Rimegepant 75 mg ODT n = 96	Placebo n = 100
Patients with PGI-C data at 24 h post dose	83 (86.5)	82 (82.0)
Improved^a^	66 (79.5)	62 (75.6)
No change	15 (18.1)	17 (20.7)
Worsening^b^	2 (2.4)	3 (3.7)
Odds ratio for improvement (Wald 95% CI; rimegepant vs placebo)^c^	1.25 (0.60, 2.59)	

All values are n (%).

The PGI-C assesses how a patient’s current illness has changed relative to baseline using a 7-point Likert scale: 1 = very much improved, 2 = much improved, 3 = minimally improved, 4 = no change, 5 = minimally worse, 6 = much worse, and 7 = very much worse. The percentages were based on the number of mITT subjects with non-missing PGI-C data at 24 h post dose.

^a^The categories with values of 1–3 on the Likert scale were combined into a single “Improved” category.

^b^The categories with values of 5–7 on the Likert scale were combined into a single “Worsening” category.

^c^A positive odds ratio may indicate that rimegepant is more likely than placebo to improve.

Abbreviations: CI, confidence interval; mITT, modified intent-to-treat; ODT, orally disintegrating tablet; PGI-C, Patient Global Impression of Change.

The observed mean (at baseline and 24 hours post dose) and LS mean CFB at 24 hours after dosing for the SNOT-22 questionnaire score are summarized in Table 3. There were no significant treatment differences (95% CI, nominal P value) between groups at 24 hours post dose for the SNOT-22 total score (−3.5 [−9.29, 2.29], P = 0.234) and individual domain scores, including nasal (−1.4 [−3.61, 0.87], P = 0.230), ear/facial (0.38 [−1.52, 0.77], P = 0.516), sleep (−1.00 [−2.48, 0.48], P = 0.183), function (−0.58 [−1.63, 0.48], P = 0.284), and emotional (−0.28 [−1.10, 0.53], P = 0.493).

**Table 3:** SNOT-22 total and domain scores (mITT analysis set).

	Treatment arm		Difference between treatment arms	P value
	Rimegepant 75 mg ODT n = 96	Placebo n = 100		
**Total**				
Mean (SD)				
Baseline^a^	48.6 (19.97)	44.5 (16.51)	–	–
24 h post dose^b^	37.4 (20.89)	39.8 (20.43)	–	–
LS mean CFB at 24 h post dose (95% CI)^b^	−9.32 (−13.89, −4.75)	−5.82 (−10.51, −1.12)	−3.50 (−9.29, 2.29)	0.234
**Domain**				
Nasal				
Mean (SD)				
Baseline, mean^a^	18.5 (7.99)	18.5 (7.46)	–	–
24 h post dose^b^	14.2 (8.61)	15.5 (7.41)	–	–
LS mean CFB at 24 h post dose (95% CI)^b^	−4.09 (−5.86, −2.31)	−2.72 (−4.55, −0.89)	−1.37 (−3.61, 0.87)	0.230
Ear/facial				
Mean (SD)				
Baseline^a^	8.3 (4.49)	8.4 (3.98)	–	–
24 h post dose^b^	6.4 (3.96)	7.1 (4.36)	–	–
LS mean CFB at 24 h post dose (95% CI)^b^	−1.98 (−2.88, −1.08)	−1.60 (−2.53, −0.68)	−0.38 (−1.52, 0.77)	0.516
Sleep				
Mean (SD)				
Baseline^a^	10.7 (5.32)	9.1 (5.09)	–	–
24 h post dose^b^	8.2 (5.49)	8.4 (5.70)	–	–
LS mean CFB at 24 h post dose (95% CI)^b^	−1.96 (−3.12, −0.80)	−0.96 (−2.15, 0.23)	−1.00 (−2.48, 0.48)	0.183
Function				
Mean (SD)				
Baseline^a^	7.2 (4.27)	5.7 (4.30)	–	–
24 h post dose^b^	5.6 (3.90)	5.7 (4.02)	–	–
LS mean CFB at 24 h post dose (95% CI)^b^	−1.02 (−1.85, −0.19)	−0.44 (−1.30, 0.42)	−0.58 (−1.63, 0.48)	0.284
Emotional				
Mean (SD)				
Baseline^a^	3.9 (3.40)	2.8 (3.05)	–	–
24 h post dose^b^	3.1 (2.81)	3.0 (3.12)	–	–
LS mean CFB at 24 h post dose (95% CI)^b^	−0.32 (−0.96, 0.31)	−0.04 (−0.70, −0.62)	−0.28 (−1.10, 0.53)	0.493

Analysis of SNOT-22 scores was based on ANCOVA models with baseline score as a covariate and treatment group and stratification factor (presence or absence of nasal polyps) as fixed effects.

Baseline SNOT-22 was defined as the last non-missing value during the pre-treatment analysis period.

^a^Number of participants at baseline: rimegepant, n = 96; placebo, n = 100.

^b^Number of participants at 24 h post dose: rimegepant, n = 83; placebo, n = 82.

Abbreviations: ANCOVA, analysis of covariance; CFB, change from baseline; CI, confidence interval; LS, least-squares; mITT, modified intent-to-treat; ODT, orally disintegrating tablet; SD, standard deviation; SNOT-22, 22-item Sino-Nasal Outcome Test.

### Safety outcomes

Overall, five all-cause AEs occurred in four participants in the rimegepant group, and five all-cause AEs occurred in five participants in the placebo group (Table 4). All on-treatment AEs were mild or moderate in severity, and there was only a single incidence for each preferred term of AE. None of the AEs related to the study drug (rimegepant [n = 1], dry throat; placebo [n = 2], nausea and hypertransaminasemia) were considered serious. At the end of the study, clinical laboratory tests, ECG tests, and vital signs showed no significant changes from baseline in either treatment arm. Similarly, there were no significant differences in the C-SSRS score between treatment groups at the end of the study.

**Table 4:** Adverse events during the on-treatment period (safety analysis set).

	Treatment arm		Total (n = 204)
	Rimegepant 75 mg ODT (n = 99)	Placebo (n = 105)	
Patients with adverse events	4 (4.0)	5 (4.8)	9 (4.4)
Adverse event related to study drug	1 (1.0)	2 (1.9)	3 (1.5)
Severe adverse event	0	0	0
Serious adverse event	0	0	0
Adverse events by preferred term			
Aspartate aminotransferase increased	1 (1.0)	0	1 (0.5)
Blood creatine phosphokinase increased	1 (1.0)	0	1 (0.5)
Back pain	0	1 (1.0)	1 (0.5)
Diarrhea	0	1 (1.0)	1 (0.5)
Dry throat	1 (1.0)	0	1 (0.5)
Electrocardiogram PR interval shortened	0	1 (1.0)	1 (0.5)
Head injury	1 (1.0)	0	1 (0.5)
Hypertransaminasemia	0	1 (1.0)	1 (0.5)
Nausea	0	1 (1.0)	1 (0.5)
Upper respiratory tract infection	1 (1.0)	0	1 (0.5)

All values are n (%).

Abbreviation: ODT, orally disintegrating tablet.

## Discussion

This phase 2/3 trial evaluated the acute efficacy and safety of a single dose of rimegepant 75 mg ODT in participants with CRS with (20%) or without (80%) nasal polyps. The study findings showed similar efficacy of rimegepant 75 mg ODT and placebo among all participants with CRS. There were no significant differences between the treatment groups in the primary, secondary, and exploratory efficacy outcomes. Also, results for the efficacy endpoints were consistent when participants were categorized according to the presence or absence of nasal polyps. The safety findings demonstrated that treatment with rimegepant was well tolerated.

To our knowledge, this is the first study to assess the therapeutic effects of CGRP receptor antagonists in CRS. Although the similar treatment effects of rimegepant and placebo observed in this study may question the role of CGRP-related signaling pathways in the pathophysiology of CRS, the implications of these findings should be considered in light of several limitations. The trial was designed to determine acute treatment effects of a single dose of rimegepant, taken once following a qualifying event of facial pain/pressure/fullness (intensity ≥6 on the NRS). Efficacy outcomes were assessed at 2 or 24 hours post dose. Testing different doses, dosing frequency, and treatment duration could yield different results. Although a previous dose ranging study suggested no additional benefit from rimegepant doses >75 mg for the acute treatment of migraine [25], this may be different in other pain models or conditions. Moreover, in a recent phase 4 study, the efficacy response for rimegepant 75 mg taken once daily appeared more robust compared with dosing every other day for the prevention of episodic migraine [26]. Additionally, future studies could investigate the potential preventive role of CGRP receptor antagonism in CRS over a longer time. Although no interventional studies have used CGRP receptor antagonists in patients with CRS, significant placebo effects in trials involving biologics and steroids have been reported to influence patient-reported and objective outcomes, broadly similar to the reduction in SNOT-22 and nasal obstruction scores observed in this study [27–29]. A reduction of 9 points in the SNOT-22 score could be clinically meaningful and indicative of improved CRS symptoms [28]. In this study, participants treated with rimegepant had a numerical decrease for the SNOT-22 total score of −9.3 (LS mean CFB) vs −5.8 in the placebo group at 24 hours post dose. These data illustrate the importance of employing an adequate control group in assessing the potential efficacy of an acute intervention in CRS. Moreover, the number of participants per treatment arm (≤100) in the mITT analysis set, along with placebo effects that could have been underestimated, may have underpowered the study to detect smaller differences between the rimegepant- and placebo-treated groups. Nonetheless, the lack of numerical trends, comparing rimegepant versus placebo, across outcome measures used to evaluate acute efficacy does not suggest a therapeutic benefit of CGRP antagonists in the acute treatment of CRS.

CRS significantly reduces the QoL of patients and PROMs can capture an individual’s assessment of the severity, changes, and QoL impact of their disease. Several PROMs have been extensively validated for CRS, such as the most used SNOT-22, whereas others are potentially more prone to subjective variability and require further validation (e.g., TNSS) [30,31]. However, in this study, SNOT-22 was not used with a 2-week recall period. This trial focused on patient-reported efficacy measures to determine the acute treatment effects of rimegepant in CRS, but future studies may benefit from evaluating other PROMs, incorporating functional outcomes, and identifying an optimal primary efficacy endpoint.

## Conclusions

To our knowledge, this study is the first to explore the use of CGRP receptor antagonists in participants with CRS. No treatment differences were identified between the rimegepant- and placebo-treated groups. Although the study was negative, the findings could contribute to the design of future clinical trials and better understanding of the disease.

## Supporting information

S1 Fig Mean observed NRS score for face pain/pressure/fullness (A), nasal obstruction/congestion (B), and nasal discharge (C), and total nasal symptom score (D). Abbreviations: mITT, modified intent-to-treat; NRS, Numerical Rating Scale; ODT, orally disintegrating tablet; SD, standard deviation.
(PDF)

S2 Fig Mean observed NRS score for face pain/pressure/fullness (A), nasal obstruction/congestion (B), and nasal discharge (C), and total nasal symptom score (D) of participants categorized based on the presence or absence of nasal polyps. Abbreviations: CRSsNP, chronic rhinosinusitis without nasal polyp; CRSwNP, chronic rhinosinusitis with nasal polyp; NRS, Numerical Rating Scale; ODT, orally disintegrating tablet; SD, standard deviation.
(PDF)

S1 File Supplemental methods. (DOCX)

S2 File Study protocol. (PDF)

S3 File CONSORT checklist. (DOCX)
