# Supplementary material for: Efficacy and safety of rimegepant 75 mg orally disintegrating tablet for the acute treatment of chronic rhinosinusitis in adults: Results from a multicenter, randomized, placebo-controlled, phase 2/3 trial
Source: PLoS One. 2026 Mar 4;21(3):e0342907. doi: 10.1371/journal.pone.0342907 (PMC12959675; doi:10.1371/journal.pone.0342907)
Supplement: S1 Fig — Abbreviations: mITT, modified intent-to-treat; NRS, Numerical Rating Scale; ODT, orally disintegrating tablet; SD, standard deviation. (PDF) [file pone.0342907.s001.pdf]

S1 Fig. Mean observed NRS score for face pain/pressure/fullness (A), nasal obstruction/congestion (B), and nasal discharge (C), and total nasal symptom score (D).

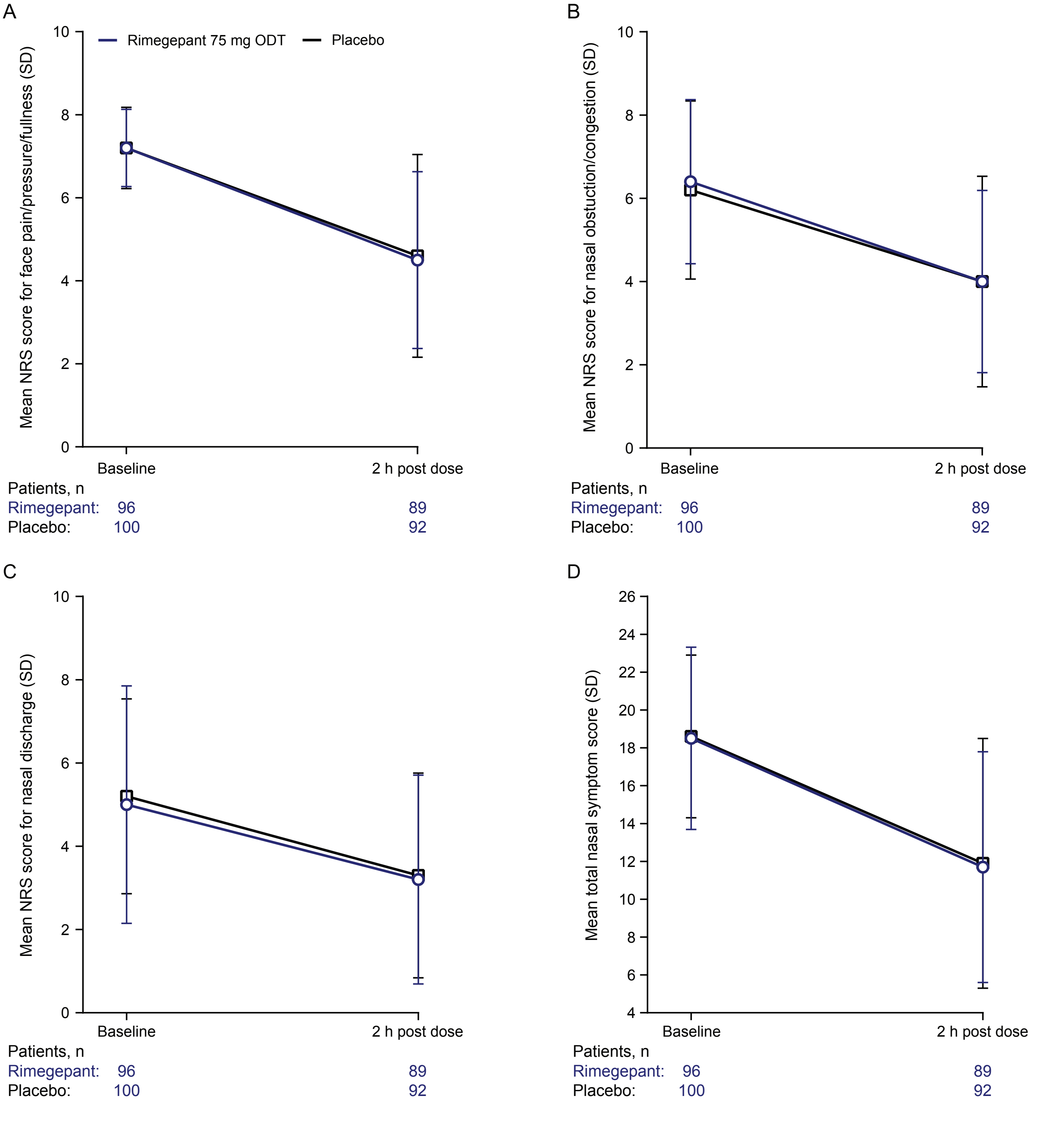

Abbreviations: mITT, modified intent-to-treat; NRS, Numerical Rating Scale; ODT, orally disintegrating tablet; SD, standard deviation.
