# Supplementary material for: Efficacy and safety of rimegepant 75 mg orally disintegrating tablet for the acute treatment of chronic rhinosinusitis in adults: Results from a multicenter, randomized, placebo-controlled, phase 2/3 trial
Source: PLoS One. 2026 Mar 4;21(3):e0342907. doi: 10.1371/journal.pone.0342907 (PMC12959675; doi:10.1371/journal.pone.0342907)
Supplement: S1 File — (DOCX) [file pone.0342907.s003.docx]

**Supplemental Methods**

**Sample size calculation**

Roughly 200 patients (100 per treatment arm) were expected in the modified intent-to-treat analysis set based on the expectation that 70% of the 286 randomized patients (143 per treatment arm) will have qualifying facial pain/pressure/fullness (pain intensity ≥6 on the Numeric Rating Scale [NRS, range 0–10]) within the allotted time period and complete their eDiary in the 24 hours following treatment. The study was expected to have approximately 88% power for the primary endpoint if rimegepant 75 mg ODT provided a 2-point reduction in NRS pain, a 1.35-point advantage over placebo on the primary endpoint, and a common standard deviation (SD) of 3.0. The estimates for the change from baseline in the NRS and common SD were consistent with modest reduction in pain over placebo and a conservative SD estimate, as these varied widely in previous studies [1].

**Reference**

[1] Salaffi F, Stancati A, Silvestri CA, Ciapetti A, Grassi W. Minimal clinically important changes in chronic musculoskeletal pain intensity measured on a numerical rating scale. Eur J Pain. 2004;8(4):283-91. Epub 2004/06/23. doi: 10.1016/j.ejpain.2003.09.004.
