# Supplementary material for: Efficacy and safety of rimegepant 75 mg orally disintegrating tablet for the acute treatment of chronic rhinosinusitis in adults: Results from a multicenter, randomized, placebo-controlled, phase 2/3 trial
Source: PLoS One. 2026 Mar 4;21(3):e0342907. doi: 10.1371/journal.pone.0342907 (PMC12959675; doi:10.1371/journal.pone.0342907)
Supplement: S2 File — (PDF) [file pone.0342907.s004.pdf]

090177e19e292258\Approved\Approved On: 28-Jul-2023 15:57 (GMT)

**DRUG:**	BHV-3000 (PF-07899801) (rimegepant)
**STUDY NUMBER(S):**	BHV3000-316 (C4951015)
**PROTOCOL TITLE:**	A Phase 2/3, Double-Blind, Randomized, Placebo-Controlled, Safety and Efficacy Trial of BHV-3000 (rimegepant) Orally Disintegrating Tablet (ODT) for the Acute Treatment of Chronic Rhinosinusitis (CRS) With or Without Nasal Polyps
**IND NUMBER:**	IND 158957
**SPONSOR:**	Pfizer Inc.<br>66 Hudson Boulevard East<br>New York, NY 10001
**ORIGINAL PROTOCOL DATE:**	21 October 2021
**VERSION NUMBER:**	4.0
**VERSION DATE:**	14 July 2023

This amendment incorporates all revisions to date, including amendments made at the request of country health authorities and IRBs/ECs, and any protocol administrative change letter(s).

090177e19e292258\Approved\Approved On: 28-Jul-2023 15:57 (GMT)

PROTOCOL AMENDMENT SUMMARY OF CHANGES TABLE

Version 4.0 – Amendment 3 (07 July 2023)

Overall Rationale for the Amendment: Change in sponsorship from Biohaven to Pfizer with alignment of the protocol template.

Description of Change	Brief Rationale	Section # and Name
**Substantial Modification(s)**		
Referenced study number BHV3000-316 to C4951015 and compound name BHV-3000 to PF-07899801 to reflect identification changes by sponsor.	Reflects change in sponsorship protocol and compound identification numbers	Study Synopsis<br>1.1 Background<br>1.3 Study Rationale<br>7.1.1 Investigational Product Headers<br>Title page
Sponsor name changed	Reflects transfer of sponsorship from Biohaven Pharmaceuticals Holding Company Limited to Pfizer Inc.	Throughout the document
Referenced study number BHV3000-316 to C4951015 and compound name BHV3000 to PF-07899801 to reflect identification changes by sponsor	To reflect sponsorship change, protocol and compound identification numbers	Protocol cover page<br>Study schematic<br>1.1 Background<br>1.3 Study rationale<br>4.2 Schedule of Assessments
Planned number of subjects randomized increased (from approximately 250 to approximately 286)	To account for the percentage of subjects not having a facial pain/pressure/fullness which reaches pain intensity of ≥ 6 on the NRS (0-10)	Study Summary (Synopsis)<br>1.3.1 Study design rationale<br>5.1 Number of Subjects<br>4.2.2 Acute treatment (Randomization) phase<br>9.2 Sample size

Inclusion criterion #1 "Signed writtern Informed Consent" deleted	To align with Pfizer's standards and processes	5.2. Inclusion Criteria
Inclusion criterion #2.b and Exclusion criterion #4.a for reproductive status updated	To align with Pfizer's standards and processes	5.2. Inclusion Criteria<br>5.3. Exclusion Criteria
Addition of an exclusion criterion #7.i for site staff, sponsor employees and families	To align with Pfizer's standards and processes	5.3. Exclusion Criteria
Addition of an exclusion criterion #7.j "Subject unable to complete eDiary independently in the opinion of the Investigator."	To ensure eDiary data is collected appropriately	5.3. Exclusion Criteria
Exclusion criterion #2.a for body mass index changed to > 35.0 kg/m2 (instead of ≥ 33.0 kg/m2)	To align with the definition of all Class 1 Obesity subjects	5.3 Exclusion Criteria
Exclusion criterion #2.b for "HIV disease" deleted	To align with rimegepant prescribing information where there is no contraindication for use	5.3 Exclusion Criteria
Exclusion criterion #2.j for "History of hematologic or solid malignancy diagnosis within 5 years prior to screening" deleted	Exclusion criterion already covered in another Exclusion criterion and to align with rimegepant prescribing information where there is no contraindication for use	5.3 Exclusion Criteria
Exclusion criterion #5.a for eGFR criteria changed to < 30 ml/min/1.73m2	Allowed for subjects with moderate renal impairment to be included in the trial consistent with the prescribing information	5.3 Exclusion Criteria
Exclusion criteria #5.b-e for "QTcF, Left Bundle Branch Block, Right Bundle Branch Block and Intra Conduction defect" deleted	To align with rimegepant prescribing information where there is no contraindication for use	5.3 Exclusion Criteria

090177e19e292258\Approved\Approved On: 28-Jul-2023 15:57 (GMT)

Exclusion criterion #5.b for "ECG abnormalities that, in the Investigator's opinion, makes the subject unsuitable for participation in the study" added	To increase study inclusivity and to align with rimegepant prescribing information where there is no contraindication for use	5.3 Exclusion Criteria
Exclusion criterion #5.f for liver enzymes (ALT and AST) changed to >2 x ULN (instead >1xULN) and #5.c total bilirubin changed to >1.5 x ULN (instead >1x ULN). For Gilbert's syndrome, direct bilirubin >ULN is exclusionary.	To align with rimegepant prescribing information where there is no contraindication for use	5.3 Exclusion Criteria
Exclusion criterion #5.e for HbA1c changed to > 7.5 % (instead of ≥ 6.5%)	To allow the inclusion of well controlled diabetics, aligned with rimgepant prescribing information where there is no exclusion for subjects with diabetes	5.3 Exclusion Criteria
Exclusion criterion #6.f for prior monoclonal antibody use changed from 6 months to 3 months prior to screening	To increase study inclusivity and to align with rimegepant prescribing information where there is no contraindication for use	5.3 Exclusion Criteria<br>5.4 Prohibited Medications
Addition of an exclusion criterion #6.j "Use of any prohibited concomitant medication(s) as specified in Section 5.4."	To align with Section 5.4 Prohibited and Restricted Concomitant Medications	5.3. Exclusion Criteria
Aspirin dose allowed changed from 81 mg to up to 100 mg	To take into account commonly prescribed dose in cardiovascular prophylaxis	5.4 Prohibited and Restricted Concomitant Medications
Guidance relative to Women of Chilbearing Potential replaced with Contraception section and Appendix III	To align with Pfizer's standards and processes	5.6 Contraception<br>16.3 Appendix III: Contraceptive and Barrier Guidance
Addition of guidance relative to abnormal laboratory tests	To align with Pfizer's standards and processes	6.2.4 Laboratory assessments

090177e19e292258\Approved\Approved On: 28-Jul-2023 15:57 (GMT)

Addition of guidance relative to ECG management and addition of a new appendix relative to ECG findings of Potential Clinical Concern	To align with Pfizer's standards and processes	6.2.2 ECG<br>16.2: APPENDIX II: ECG findings of Potential Clinical Concern
Statement regarding any "yes" responses on the C-SSRS questionnaire updated	To align with Exclusion criterion #7.f	6.2.5 Columbia-Suicide Severity Rating Scale (C-SSRS)
Removed Non-Investigational Product section	Not applicable for this study	7.1.2. Non-Investigational Product
Potential DILI cases identification and management update	To align with Pfizer's standards and processes	8.5 Potential Drug Induced Liver Injury (DILI)
Updated Overdose section	To clarify that only overdose associated with an SAE is reportable to Pfizer Safety	8.3 Overdose
Re-screening policy updated	To allow and define rescreening criteria for previously screen failed subjects	4.2.1 Screening phase
Updated the information about the known and expected benefits and risks and reasonably expected AEs of rimegepant and referenced to the Investigator Brochure	To align with Investigator brochure	1.2 Product development background<br>1.3.4 Benefit/Risk assessment
Description of primary estimand added	Primary estimand description was not included in earlier protocol versions	9.3 Populations for Analysis and Estimands
Deleted "*subject as random effect*" and changed "*generalized linear mixed effect model*" to "*linear model*" from the model specification of primary and secondary analysis	A repeated measure within subject is already considered in MMRM analysis to address within subject correlation. Additional specification of random effect for subject is misleading to the context.	9.4. Statistical methods
Addition of "Sensitivity analysis"	Robustness of primary estimand (MAR assumption) will be verified through additional sensitivity analysis	9.4.2 Primary Endpoint(s)

090177e19e292258\Approved\Approved On: 28-Jul-2023 15:57 (GMT)

Replaced "*stratified Cochran-Mantel-Haenszel (CMH) test ... stratification factor as...*" with "*Mantel-Haenszel risk estimation .... stratification by ...*"	Correct reference to test procedure	9.4.3. Secondary Endpoints
Addition of the section regarding Sponsor's Medically Qualified Individual	To align with Pfizer's standards and processes	10.6 Sponsor's Medically Qualified Individual
Deletion of tacrolimus	To align with nonclinical and clinical drug to drug interaction information available on rimegepant	16.1 APPENDIX I: Strong Inhibitors and Strong and Moderate Inducers of CYP3A4 Protein (Not all-inclusive)
Addition of moderate CYP3A4 inducers	To align with nonclinical and clinical drug to drug interaction information available on rimegepant	16.1 APPENDIX I: Strong Inhibitors and Strong and Moderate Inducers of CYP3A4 Protein (Not all-inclusive)
Addition of Contraceptive and Barrier Guidance	To align with Pfizer's standards and processes	16.3 APPENDIX III: Contraceptive and Barrier Guidance
Defined End of Study	To align with Pfizer's standards and processes	4.1 Study Design and Duration
**Non-substantial Modification(s)**		
Clarified IP administration	To align with Section 7.2.2 Selection and Timing of Dose and Administration	1.3.1 Study Design Rationale<br>4.1 Study Design and Duration
Updated the Serious Adverse Event (SAE) and pregnancy reporting destination and electronic reporting system; updated SAE reporting requirements	To incorporate the non-substantial changes described in previous PACL dated 28Apr2023	8.1.2 Collection and reporting Serious Adverse Events<br>8.4 Pregnancy
Addition of Pregnant Partner Release of Information Form	To align with Pfizer's standards and processes	8.4 Pregnancy

090177e19e292258\Approved\Approved On: 28-Jul-2023 15:57 (GMT)

Updated the non-Serious Adverse Events reporting starting point	To align with Pfizer's standards and processes regarding AE reporting starting point	8.2.1 Collection and reporting of non-Serious Adverse Events
Reporting requirements added for:<br>Environmental Exposure, Exposure During Breastfeeding, Occupational Exposure;<br>Lack of Efficacy;<br>Medication Errors;	To align with Pfizer's standards and processes	8.7 Environmental Exposure, Exposure During Breastfeeding and Occupational Exposure<br>8.8 Lack of Efficacy<br>8.9 Medication Errors
Approximative number of sites and study duration deleted	To align with Pfizer's standards	5.1 Number of subjects
Clarified that daily medication doses must be stable for at least one month prior to the Screening Visit and throughout the study	Correction	5.5 Daily and Rescue Medications
Addition of reference to Quality Tolerance Limits and renamed the section	To align with Pfizer's standards and processes	5.8 Protocol deviations and Quality Tolerance Limits
Addition of Investigational Product manual and Pregnant Partner Release of Information Form; Updated forms and removed reference to eCRF for SAE reporting	To align with Pfizer's processes	6.1 Study materials
Replacement of "Pharmacy Manual" with "Investigational Product Manual" and removal of "humidity" from environmental condition requirements	To align with Pfizer's standards and processes	7.1.2 Packaging, Shipment and Storage

090177e19e292258\Approved\Approved On: 28-Jul-2023 15:57 (GMT)

Statement regarding public registration of Phase 1 trials in publicly accessible database deleted. Statement regarding Principal Investigator and the sponsor's representative signatory deleted. Sponsor's regulatory and ethics responsibilities added	To align with Pfizer's standards and processes	10.1 Good Clinical Practice
New section regarding Dissemination of Clinical Study Data	To align with Pfizer's standards regarding dissemination of Clinical Study Data	10.2 Dissemination of Clinical Study Data
Clarified that subject must sign and date an IRB/IEC approved written informed consent form for study	To align with Pfizer's standards and processes	10.4 Informed Consent
Removed statement that IRB/IEC has to be notified at least 5 days prior to implementing protocol change	To align with Pfizer's standards and processes	12 Amendments
Section 13 "Study report and Publications policy" renamed to "Publication policy"; sentence regarding CSR deleted	To account for the addition of Section 10.2 Dissemination of Clinical Study Data	13 Publication policy
Section 15 "Confidentiality" renamed to "Data Protection" and updated	To align with Pfizer's standards and processes	15 Data protection
Moved past protocol amendments section "Summary of Changes" to Appendix	To align with Pfizer's editorial standards	16.3 APPENDIX IV: Protocol Amendment History
Sections "Confidentiality and Investigator statement" and "Clinical Protocol Approval form" removed	To align with Pfizer's standards	Confidentiality and Investigator statement<br>Clinical Protocol Approval form

090177e19e292258\Approved\Approved On: 28-Jul-2023 15:57 (GMT)

Replacement of "participant" with "subject"	For consistency throughout the protocol	Throughout the protocol
Renumbering of Inclusion and Exclusion criteria	Editorial change	5.2 Inclusion criteria<br>5.3 Exclusion criteria
List of abbreviations updated	To reflect new abbreviations added to the Protocol amendment	List of abbreviations
Minor grammatical and spelling corrections; Section renumbering	Corrections	Throughout the document

090177e19e292258\Approved\Approved On: 28-Jul-2023 15:57 (GMT)

STUDY SUMMARY (SYNOPSIS)

**Title:**	A Phase 2/3, Double-Blind, Randomized, Placebo-Controlled, Safety and Efficacy Trial of BHV-3000 (PF-07899801) (rimegepant) Orally Disintegrating Tablet (ODT) for the Acute Treatment of Chronic Rhinosinusitis (CRS) With or Without Nasal Polyps
**Rationale:**	Chronic rhinosinusitis (CRS) is a symptomatic inflammation of the paranasal sinuses and nasal cavity lasting more than 12 weeks. Broadly, it can be divided into two phenotypes: CRS with and without nasal polyps. Typically, it presents with facial pain/pressure/fullness, nasal obstruction (congestion), nasal discharge, and/or decreased sense of smell. CRS affects approximately 5-12% of the US population and is associated with reduced quality of life and significant health care burden.[1-3]<br><br>The mainstay of treatment are topical nasal corticosteroids and saline irrigation; however, the efficacy is very limited leaving many patients without achieving desirable relief. In contrast, oral corticosteroids, long-term non-steroidal anti-inflammatory drugs (NSAIDs), or antibiotics may provide more benefits but are associated with significant side effects. With limited treatment options and inadequate disease management, CRS remains an area of unmet medical need prompting development of disease-specific interventions.[4,5]<br><br>The proposed study is based on the evolving preclinical and clinical evidence suggesting a role for calcitonin gene-related peptide (CGRP) in the pathophysiology of CRS. BHV-3000 (PF-07899801) (rimegepant) is a small molecule CGRP receptor antagonist that is being developed for the potential treatment of CRS. The data from this study will allow characterization of the safety and efficacy of orally disintegrating tablet (ODT) of rimegepant versus placebo in the treatment of chronic rhinosinusitis with or without polyps.
**Target Population:**	The study will recruit male and female subjects 18 years of age and older with a history of chronic rhinosinusitis with or without nasal polyps, diagnosed by a healthcare provider, that have had at least a twelve week history of facial pain/pressure/fullness and one or more of the following signs and symptoms: nasal discharge (anterior, posterior, or both), nasal obstruction (congestion), and/or decreased sense of smell. Subjects must have had inflammation documented by one or more of the following findings: purulent (not clear) mucus or edema in the middle meatus or anterior ethmoid region; polyps in nasal cavity or the middle meatus; and/or radiographic imaging showing inflammation of the paranasal sinuses.<br><br>Subjects must have had at least two instances of facial pain/pressure/fullness that had reached a current intensity of moderate or severe on a 4-point rating

090177e19e292258\Approved\Approved On: 28-Jul-2023 15:57 (GMT)

	scale (0 = None, 1 = Mild, 2 = Moderate, 3 = Severe) within 30 days prior to the Screening Visit.
**Number of Subjects:**	Approximately 286 subjects will be randomized to result in approximately 200 (100 per arm) evaluable subjects. The subjects will be randomized in a 1:1 ratio to the rimegepant or placebo treatment groups. The randomization will be stratified by the presence of nasal polyps (yes or no).
**Objectives (Primary):**	To evaluate the efficacy of rimegepant compared with placebo in the acute treatment of chronic rhinosinusitis with or without polyps on mean change from baseline of facial pain/pressure/fullness on a Numerical Rating Scale (NRS) (0-10) score at 2 hours post-dose.
**Objectives (Secondary):**	1. To evaluate rimegepant compared to placebo on change from baseline in Total Nasal Symptom Score (TNSS) at 2 hours post-dose.<br>2. To evaluate rimegepant compared to placebo on change from baseline in nasal obstruction (congestion) score on NRS (0-10) score at 2 hours post-dose.<br>3. To evaluate rimegepant compared to placebo on change from baseline in nasal discharge score on NRS (0-10) score at 2 hours post-dose.<br>4. To evaluate rimegepant compared to placebo on headache pain relief on a 4-point Likert scale at 2 hours post-dose compared to baseline.<br>5. To evaluate rimegepant compared to placebo on the probability of requiring rescue medication within 24 hours of initial treatment.<br>**Exploratory Objectives**<br>1. To evaluate rimegepant compared to placebo on ≥ 30% reduction from baseline of facial pain/pressure/fullness NRS (0-10) score at 2 hours post-dose.<br>2. To evaluate rimegepant compared to placebo on ≥ 50% reduction from baseline of facial pain/pressure/fullness NRS (0-10) score at 2 hours post-dose.<br>3. To evaluate the effect of rimegepant compared to placebo on change from baseline of facial pain/pressure/fullness NRS (0-10) score at 15, 30, 45, 60, 90 minutes and 2, 4, 8 and 24 hours post-dose.<br>4. To evaluate the effect of rimegepant compared to placebo on change from baseline nasal obstruction (congestion) NRS (0-10) score at 15, 30, 45, 60, 90 minutes and 2, 4, 8 and 24 hours post-dose.

090177e19e292258\Approved\Approved On: 28-Jul-2023 15:57 (GMT)

	5. To evaluate the effect of rimegepant compared to placebo on change from baseline of nasal discharge NRS (0-10) score at 15, 30, 45, 60, 90 minutes and 2, 4, 8 and 24 hours post-dose.<br><br>6. To evaluate the effect of rimegepant compared to placebo headache pain relief at 15, 30, 45, 60, 90 minutes and 2, 4, 8 and 24 hours post-dose compared to baseline.<br><br>7. To evaluate rimegepant compared to placebo on the PGI-C questionnaire at 24 hours post-dose.<br><br>8. To evaluate rimegepant compared to placebo on ≥ 30% reduction of facial pain/pressure/fullness NRS (0-10) score at 24 hours post-dose.<br><br>9. To evaluate rimegepant compared to placebo on ≥ 50% reduction of facial pain/pressure/fullness NRS (0-10) score at 24 hours post-dose.<br><br>10. To evaluate rimegepant compared to placebo on sustained relief of facial pain/pressure/fullness, as defined by each of the following: 30% reduction from baseline on NRS (0-10), 1.5-point reduction on NRS (0-10), and a 2-point reduction on NRS (0-10) through 24 hours post-dose.<br><br>11. To explore the distribution of baseline disease severity using the of Sino-Nasal Outcome Test (SNOT-22).<br><br>12. To evaluate rimegepant compared to placebo on the Sino-Nasal Outcome Test (SNOT-22) score at 24 hours post-dose.<br><br>13. To evaluate the safety and tolerability of rimegepant in the acute treatment of chronic rhinosinusitis as measured by the frequency of adverse events of moderate or severe intensity, serious adverse events (SAEs), clinically relevant laboratory abnormalities, and nasal inspection abnormalities.<br><br>14. To evaluate rimegepant compared to placebo for the Columbia-Suicide Severity Rating Scale (C-SSRS).
**Study Design:**	This is a double-blind, randomized, multicenter, outpatient evaluation of the safety and efficacy of rimegepant as compared to placebo for the acute treatment of chronic rhinosinusitis (CRS) with or without nasal polyps. The study drug will be rimegepant presented in a 75 mg ODT or matching placebo.<br><br>Subjects who have consented to study participation will first participate in the screening phase (3 to 14 day period). Subjects must remain on the same dose of daily use of medications to treat CRS symptoms from screening through the duration of the study and may not start any new daily medication. Subjects meeting initial eligibility criteria will be asked to continue to a Baseline Visit.

090177e19e292258\Approved\Approved On: 28-Jul-2023 15:57 (GMT)

	At the Baseline Visit, eligibility for continued participation in the study will be assessed before randomization occurs and before study medication is dispensed. The subject will be dispensed a single dose of the double-blind study medication. The subject will be instructed to take their study medication, as an outpatient, when (if) they have a facial pain/pressure/fullness which reaches a current intensity of ≥ 6 on the NRS (0-10). The subject will complete questionnaires on an eDiary for 24 hours after taking study medication. The subject will be instructed to telephone the study center immediately if a severe or serious adverse event occurs.<br><br>Subjects will record efficacy data in their eDiary. This includes the following: intensity of the facial pain/pressure/fullness prior to and at time of taking study medication. Subject should not dose with study medication until facial pain/pressure/fullness reaches a current intensity of ≥ 6 on the NRS (0-10). Subjects will complete Sino-Nasal Outcome Test (SNOT-22) questionnaire at the Baseline Visit (on a paper scale), and at 24 hours post-dose, subjects will complete SNOT-22 (on the eDiary). Facial pain/pressure/fullness severity, nasal congestion (obstruction) severity, and nasal discharge severity will be recorded using a NRS (0-10) just prior to taking study medication and at 15, 30, 45, 60, and 90 minutes and 2, 4, 8 and 24 hours after dosing. Total Nasal Symptom Score (TNSS) will be calculated as the sum of 3 symptom scores: (1) facial pain/pressure/fullness; (2) nasal obstruction (congestion); and (3) nasal discharge; recorded just prior to taking study medication and after dosing at time points of 15, 30, 45, 60, and 90 minutes and 2, 4, 8 and 24 hours. Subjects who experience current headache pain of moderate or severe pain intensity at the time of qualifying facial pain/pressure/fullness will record their headache pain severity using a 4-point Likert scale (0 = None; 1 = Mild; 2 = Moderate; 3 = Severe) at time of taking study medication and after dosing at time points of 15, 30, 45, 60, and 90 minutes and 2, 4, 8 and 24 hours. Subjects will complete the Patient Global Impression of Change (PGI-C) questionnaire 24 hours after dosing.<br><br>At the end of 2 hours after dosing with study medication (and after the 2-hour assessments have been completed on the eDiary), subjects will be permitted to use the following rescue medication: acetaminophen (up to 1000 mg/day), or aspirin, ibuprofen, naproxen (or any other type of non-steroidal anti-inflammatory drug [NSAID]), oral antihistamines (non-sedating), oral decongestants, topical nasal decongestants, topical nasal anticholinergics (all up to the daily recommended dose indicated on the drug packaging). These are the only medications allowed for rescue treatment after 2 hours post dose of study medication. At the end of 24 hours after dosing with study medication (but before the End of Treatment Visit), if needed, they may take their prescribed standard of care medications provided all of the assessments have been completed on the eDiary. Exclusionary rescue medication such as opioids

090177e19e292258\Approved\Approved On: 28-Jul-2023 15:57 (GMT)

	and butalbital compounds are not allowed on this study.<br><br>Similarly, if the facial pain/pressure/fullness is relieved by study medication at 2 hours after dosing but then recurs to a intensity of ≥ 6 on the NRS (0-10) between 2 and 24 hours, the subject will be permitted to take the same rescue therapy as outlined above. In all circumstances, the subject will always continue to complete his or her eDiary for up to 24 hours after consuming the study medication. During the 45-day treatment phase, if the subject has a **non-qualifying** instance of facial pain/pressure/fullness (i.e., facial pain/pressure/fullness < **6 on a NRS [0-10]**), the subject is permitted to use only the following medications to treat the symptoms: acetaminophen (up to 1000 mg/day), or aspirin, ibuprofen, naproxen (or any other type of non-steroidal anti-inflammatory drug [NSAID]), oral antihistamines (non-sedating), oral decongestants, topical nasal decongestants, topical nasal anticholinergics (all up to the daily recommended dose indicated on the drug packaging), that are not otherwise prohibited by the study, see Section 5.4.<br><br>Subjects should have access to systemic corticosteroids and antibiotics during the trial for treatment of symptoms, if indicated. However, subjects should discontinue the study if they receive those therapies.<br><br>Subjects will return to the study site within 7 days (+2) after taking study treatment for review of the eDiary, assessment of medication compliance, and monitoring of tolerability and safety. If a subject has NOT experienced a facial pain/pressure/fullness of sufficient severity within 45 days after randomization, they still are required to complete all EOT visit procedures. All subjects must return empty medication packaging, any unused study medication, and the eDiary to the study center.

090177e19e292258\Approved\Approved On: 28-Jul-2023 15:57 (GMT)

STUDY SCHEMATIC

BHV3000-316 (C4951015) Acute Treatment of Chronic Rhinosinusitis With or Without Nasal Polyps Study

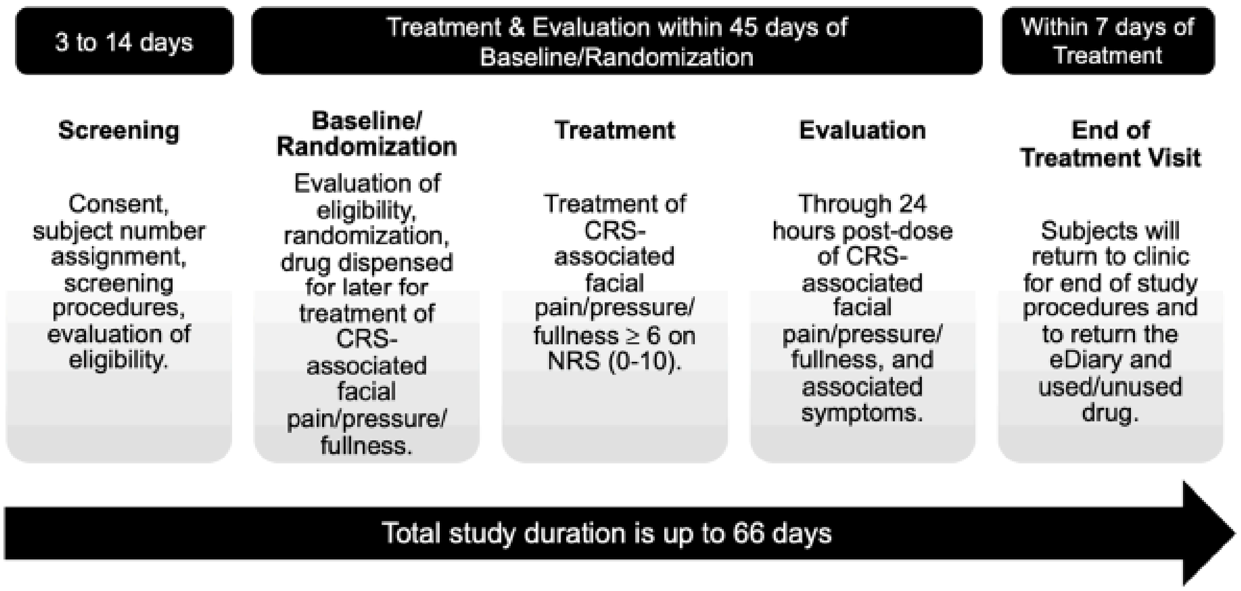

090177e19e292258\Approved\Approved On: 28-Jul-2023 15:57 (GMT)

TABLE OF CONTENTS

PROTOCOL AMENDMENT SUMMARY OF CHANGES TABLE 2
STUDY SUMMARY (SYNOPSIS) 10
STUDY SCHEMATIC 15
TABLE OF CONTENTS 16
LIST OF TABLES 19
LIST OF ABBREVIATIONS 20
1 INTRODUCTION AND RATIONALE 24
1.1 Background 24
1.2 Product Development Background 25
1.3 Study Rationale 25
1.3.1 Study Design Rationale 26
1.3.2 Dose Selection Rationale 27
1.3.3 Research Hypothesis 27
1.3.4 Benefit/Risk 27
2 STUDY OBJECTIVES 29
2.1 Primary Objective(s) 29
2.2 Secondary Objective(s) 29
2.3 Exploratory Objective(s) 29
3 STUDY ENDPOINTS 31
3.1 Primary Endpoint(s) 31
3.2 Secondary Endpoint(s) 31
3.3 Measures of interest 31
4 STUDY PLAN 32
4.1 Study Design and Duration 32
4.2 Schedule of Assessments 33
4.2.1 Screening Phase (3 to 14 days) 42
4.2.2 Acute Treatment (Randomization) Phase 42
4.2.2.1 eDiary Data Collection 42
4.2.3 Extension Phase 44
4.2.4 Washout Phase 44
4.2.5 End of Treatment 44
4.3 Post Study Access to Therapy 44
5 POPULATION 45
5.1 Number of Subjects 45
5.2 Inclusion Criteria 45
5.3 Exclusion Criteria 46
5.4 Prohibited and Restricted Concomitant Medications 51
5.5 Daily and Rescue Medications 52
5.6 Contraception 54
5.7 Other Restrictions and Precautions 54
5.8 Protocol Deviations and Quality Tolerance Limits 54
6 STUDY CONDUCT AND DESCRIPTION OF STUDY PROCEDURES 55

090177e19e292258\Approved\Approved On: 28-Jul-2023 15:57 (GMT)

6.1 Study Materials 55
6.2 Safety Assessments 56
6.2.1 Vital Signs and Physical Measurements (Height and Weight) 56
6.2.2 Electrocardiogram (ECG) 56
6.2.3 Physical Exam 56
6.2.4 Laboratory Assessments 56
6.2.4.1 Safety Laboratory Testing 57
6.2.4.2 Pregnancy Testing 58
6.2.5 Columbia-Suicide Severity Rating Scale (C-SSRS) 58
6.2.6 Weekly Contact 58
6.3 Efficacy Assessments 58
6.3.1 Facial pain/pressure/fullness 58
6.3.2 Nasal obstruction (congestion) 58
6.3.3 Nasal discharge 59
6.3.4 Headache pain 59
6.3.5 Rescue Medication 59
6.3.6 Patient Global Impression of Change (PGI-C) Questionnaire 59
6.3.7 Sino-Nasal Outcome Test (SNOT-22) 59
6.4 Early Discontinuation from the Study 59
6.5 Lost to Follow Up 60
7 STUDY DRUG MANAGEMENT 61
7.1 Description of Study Drug 61
7.1.1 Investigational Product 61
7.1.2 Packaging, Shipment and Storage 61
7.2 Dose and Administration 61
7.2.1 Method of Assigning Subject Identification 61
7.2.2 Selection and Timing of Dose and Administration 62
7.2.3 Dose Modifications 62
7.3 Blinding and Unblinding 62
7.4 Treatment Compliance 63
7.5 Destruction and Return of Study Drug 63
8 ADVERSE EVENTS 64
8.1 Serious Adverse Events 65
8.1.1 Definition of Serious Adverse Event (SAE) 65
8.1.2 Collection and Reporting Serious Adverse Events 67
8.2 Non-serious Adverse Events 68
8.2.1 Collection and Reporting of Non-Serious Adverse Events 68
8.2.2 Laboratory Test Abnormalities 68
8.3 Overdose 68
8.4 Pregnancy 68
8.5 Potential Drug Induced Liver Injury (DILI) 69
8.6 Adverse Events of Special Interest 70
8.7 Environmental Exposure, Exposure During Breastfeeding, and Occupational Exposure 71
8.7.1 Exposure During Breastfeeding 71

090177e19e292258\Approved\Approved On: 28-Jul-2023 15:57 (GMT)

8.7.2 Occupational Exposure 71
8.8 Lack of Efficacy 72
8.9 Medication Errors 72
9 STATISTICS 73
9.1 General Procedures 73
9.2 Sample Size 73
9.3 Populations for Analysis and Estimands 73
9.4 Statistical Methods 74
9.4.1 Demographic and Baseline Characteristics 74
9.4.2 Primary Endpoint(s) 75
9.4.3 Secondary Endpoint(s) 75
9.4.4 Adjustment for Multiplicity 76
9.4.5 Missing Data 77
9.4.6 Rescue Medication 77
9.4.7 Analysis of Safety 78
9.5 Schedule of Analyses 78
10 ETHICS AND RESPONSIBILITIES 79
10.1 Good Clinical Practice 79
10.2 Dissemination of Clinical Study Data 80
10.3 Institutional Review Board/Independent Ethics Committee 81
10.4 Informed Consent 81
10.5 Case Report Forms 82
10.6 Sponsor's Medically Qualified Individual 82
11 RECORDS MANAGEMENT 84
11.1 Source Documentation 84
11.2 Study Files and Record Retention 85
12 AMENDMENTS 86
13 PUBLICATION POLICY 87
14 STUDY DISCONTINUATION 88
15 DATA PROTECTION 89
16 APPENDICES 90
16.1 APPENDIX I: Strong Inhibitors and Strong and Moderate Inducers of CYP3A4 Protein (Not all-inclusive) 90
16.2 APPENDIX II: ECG Findings of Potential Clinical Concern 92
16.3 APPENDIX III: Contraceptive and Barrier Guidance 94
16.3.1 Male Subject Reproductive Inclusion Criteria 94
16.3.2 Female Subject Reproductive Inclusion Criteria 94
16.3.3 Woman of Childbearing Potential 94
16.3.4 Contraception Methods 95
16.4 APPENDIX IV: Protocol Amendment History 97
17 REFERENCES 103

090177e19e292258\Approved\Approved On: 28-Jul-2023 15:57 (GMT)

LIST OF TABLES

Table 1 BHV3000-316 (C4951015) Schedule of Assessments 33

090177e19e292258\Approved\Approved On: 28-Jul-2023 15:57 (GMT)

LIST OF ABBREVIATIONS

ACS	Acute Coronary Syndrome
ADHD	Attention-Deficit/Hyperactivity Disorder
AERD	Aspirin Exacerbated Respiratory Disease
AE	Adverse Event
ALT	Alanine Aminotransferase
AST	Aspartate Aminotransferase
AV	Atrioventricular
bpm	Beats Per Minute
BUN	Blood Urea Nitrogen
CBD	Canabidiol
CGRP	Calcitonin Gene Related Peptide
CI	Confidence Interval
CK	Creatine Kinase
CMH	Cochran-Mantel-Haenszel
COPD	Chronic Obstructive Pulmonary Disease
CRF	Case Report Form
CRO	Clinical Research Organization
CRS	Chronic Rhinosinusitis
CRSwNP	Chronic Rhinosinusitis With Nasal Polyps
CSR	Clinical Study Report
C-SSRS	Columbia-Suicide Severity Rating Scale
CT	Clinical Trial
CTCAE	Common Terminology Criteria for Adverse Events
CTIS	Clinical Trial Information System
CYP	Cytochrome P-450
DAIDS	Division of AIDS
DILI	Drug-Induced Liver Injury
DSM-V	Diagnostic and Statistical manual of Mental Disorders, Fifth edition
DSU	Drug Safety Unit

090177e19e292258\Approved\Approved On: 28-Jul-2023 15:57 (GMT)

EC	Ethics Committee
ECC	Emergency Contact Card
ECG	Electrocardiogram
eCRF	Electronic Case Report Form
EDB	Exposure During Breastfeeding
EDC	Electronic Data Capture
eDiary	Electronic Diary
EDP	Exposure During Pregnancy
EFD	Embryo-Fetal Development
eGFR	Estimated Glomerular Filtration Rate
EOD	Every Other Day
EOT	End of Treatment
ePRO	Electronic Patient Reported Outcomes
eTMF	Electronic Trial Master File
EU	European Union
EudraCT	European Union Drug Regulating Authorities Clinical Trials (European Clinical Trials Database)
FDA	Food and Drug Administration
FSH	Follicle-Stimulating Hormone
GCP	Good Clinical Practice
GGT	Gamma-Glutamyl Transferase
GLP	Good Laboratory Practice
HbA1c	Hemoglobin A1C
HCP	Healthcare Provider
HDL	High-Density Lipoprotein
HIV	Human Immunodeficiency Virus
HR	Hearth Rate
HRT	Hormone Replacement Therapy
IB	Investigator's Brochure
ICF	Informed Consent Form
ICH	International Conference on Harmonization of Technical Requirements

090177e19e292258\Approved\Approved On: 28-Jul-2023 15:57 (GMT)

	for Pharmaceuticals for Human Use
ICHD-3	The International Classification of Headache Disorders, 3rd edition
ID	Identification
IEC	Independent Ethics Committee
IND	Investigational New Drug
INR	International Normalized Ratio
IRB	Institutional Review Board
IWRS	Interactive Web Response System
LBBB	Left Bundle Branch Block
LDH	Lactate Dehydrogenase
LDL	Low-Density Lipoprotein
LFT	Liver Function Test
MAR	Missing at Random
MDRD	Modification of Diet in Renal Disease
MedDRA	Medical Dictionary for Regulatory Activities
mg	Milligram
mITT	Modified Intent To Treat
mm	Millimeter
MMRM	Mixed Model Repeated Measures
min	Minute
MI	Myocardial Infarction
MQI	Medically Qualified Individual
NOAEL	No Observed Adverse Effect Level
NRS	Numerical Rating Scale
NSAID	Non-Steroidal Anti-Inflammatory Drug
ODT	Orally Disintegrating Tablet
PACL	Protocol Administrative Change Letter
PCI	Percutaneous Coronary Intervention
PCP	Phencyclidine
PGI-C	Patient Global Impression of Change
PRN	Pro Re Nata

090177e19e292258\Approved\Approved On: 28-Jul-2023 15:57 (GMT)

PSSA	Pfizer SAE Submission Assistant
PVC	Premature Ventricular Contraction
RBC	Red Blood Cell
QTcF	QTc corrected using Fridericia's formula
QTL	Quality Tolerance Limit
SAE	Serious Adverse Event
SAP	Statistical Analysis Plan
SGOT	Serum Glutamic-Oxaloacetic Transaminase
SGPT	Serum Glutamic-Pyruvic Transaminase
SNOT-22	Sino-Nasal Outcome Test – 22
SRSD	Single Reference Safety Document
T Bili	Total Bilirubin
TIA	Transient Ischemic Attack
TNF-α	Tumor Necrosis Factor - Alpha
TNSS	Total Nasal Symptom Score
TMF	Trial Master File
ULN	Upper Limit of Normal
UK	United Kingdom
US	United States
WBC	White Blood Cell
WOCBP	Women of Child-Bearing Potential

090177e19e292258\Approved\Approved On: 28-Jul-2023 15:57 (GMT)

1 INTRODUCTION AND RATIONALE

1.1 Background

Chronic rhinosinusitis (CRS) is an inflammatory disease of the paranasal sinuses and nasal cavity lasting more than 12 weeks. Broadly, it can be divided into two phenotypes, CRS with and without nasal polyps, with approximately 20% of patients having nasal polyposis.[6]

It is estimated that CRS affects between 5-12% of the general population, however, when this is combined with documented inflammation, the prevalence is reduced to 3-6%. It predominantly affects the adult population and has significant impact on the quality of life. CRS is considered one of the top 10 most costly health conditions in the US with estimated indirect costs exceeding $22 billion per year.[7]

The exact underlying mechanisms in CRS pathophysiology are unknown and are most likely multifactorial. The disease is believed to arise from chronic inflammation of the nasal cavity and paranasal sinuses. Predisposing factors include environmental (e.g, pollution, allergens, viruses, bacteria), systemic (e.g., genetic, cystic fibrosis, ciliary defects), and local (e.g., anatomic, polyps, tumors) factors.[8] CRS is often accompanied by other morbidities, such as allergic rhinitis, asthma, and aspirin exacerbated respiratory disease (AERD).[3,9]

The diagnosis requires presence of at least two of the four cardinal symptoms (i.e., facial pain/pressure/fullness, nasal obstruction (congestion), nasal discharge, decreased sense of smell) and confirmed presence of inflammation by radiographic imaging or nasal endoscopy.[1] Cardinal symptoms vary in prevalence, with nasal obstruction or congestion reported as the most common in both patients with or without nasal polyps.[7]

The goal of treatment is to reduce the inflammation, restore mucociliary clearance and sinus drainage, and prevent infections.8 The first-line treatment typically includes topical intranasal steroids and nasal saline irrigation, however, the efficacy is limited. More effective therapies include systemic corticosteroids and long-term antibiotics, though they are associated with significant side effects.[4,5] One study found that in patients with CRSwNP and several oral corticosteroid bursts per year, 43% of patients developed osteoporosis and 49% developed secondary adrenal insufficiency.[10] Approximately half of the CRS patients will eventually undergo endoscopic surgery in order to control their symptoms, however, the majority will still have inadequate symptom relief and ~37% will need at least one revision surgery.[2,4]

BHV-3000 (PF-07899801) (rimegepant) is a calcitonin gene-related peptide (CGRP) receptor antagonist in development for the acute treatment of CRS. The CGRP receptor is located within pain-signaling pathways, intracranial arteries and the trigeminal ganglion, and its activation is thought to play a causal role in CRS pathophysiology.

090177e19e292258\Approved\Approved On: 28-Jul-2023 15:57 (GMT)

Treatment with a CGRP receptor antagonist is believed to exert multiple downstream effects that may provide benefits in treatment of CRS through the following possible mechanisms:

Blocking Neurogenic Inflammation: Binding of CGRP receptor antagonists to CGRP receptors located on satellite glial cells would inhibit inflammation caused by trigeminal nerve release of CGRP onto satellite glial cells and cell bodies of a-delta fibers in the trigeminal ganglion.

Decreasing Artery Dilation: By blocking the CGRP receptors located in smooth muscle cells within vessel walls, CGRP receptor antagonists would inhibit the pathologic dilation of arteries without the unwanted effect of active vasoconstriction.

Inhibiting Pain Transmission: Binding of CGRP receptor antagonists to CGRP receptors would suppress the transmission of pain by inhibiting the central relay of pain signals from the trigeminal nerve to the caudal trigeminal nucleus.

1.2 Product Development Background

Details of the clinical and preclinical studies are provided in the most current Investigator Brochure. A summary of the relevant data to the study are presented below.

Rimegepant is approved for the acute treatment and prevention of episodic migraine in the United States (US), United Kingdom (UK), and European Union (EU) and is well tolerated in humans when given as a single oral dose of 75 mg for the acute treatment of migraine and at a dose of 75 mg every other day (EOD) for the prevention of episodic migraine. The efficacy, safety, and tolerability of rimegepant for the acute and preventive treatment of migraine in the pediatric population is currently being evaluated.

As of 26 August 2022, more than 8900 unique subjects have participated in Phase 1 studies in healthy subjects or Phase 2 and 3 studies in subjects with migraine or refractory trigeminal neuralgia, chronic rhinosinusitis, or temporomandibular disorders; of these, approximately 6036 unique subjects have received rimegepant at any dose. Collectively, the current data demonstrates a favorable benefit-risk profile for rimegepant in the acute and preventive treatment of migraine.

Please refer to the most up-to-date Investigator's Brochure for additional information.

1.3 Study Rationale

Chronic rhinosinusitis (CRS) is a symptomatic inflammation of the paranasal sinuses and nasal cavity lasting more than 12 weeks. Typically, it manifests as facial pain/pressure/fullness, nasal obstruction (congestion), nasal discharge, and/or decreased sense of smell.[1-3]

The nasal cavity and paranasal sinuses receive sensory and nociceptive innervation from distal branches of the trigeminal nerve. Although the underlying mechanisms of CRS have not been

090177e19e292258\Approved\Approved On: 28-Jul-2023 15:57 (GMT)

fully elucidated, numerous studies have implicated trigeminal nerve and CGRP in the pathophysiology of the disease and suggested therapeutic benefits of CGRP antagonism. CGRP+ fibers are predominantly found in the maxillary sinuses and to lesser extent in other sinuses. Previous studies have suggested that CGRP contributes to nasal secretion by inducing sustained arterial vasodilation, increasing nasal blood flow, and possibly by increasing plasma flux across fenestrated capillaries.[13-18] In a clinical study, intranasal administration of CGRP in healthy volunteers resulted in increases in blood flow, the sensations of obstruction, headache, and mild sneezing.[13]

Another study found that in patients with chronic rhinosinusitis, nasal mucosa CGRP levels correlated with symptom intensity and the number of inflammatory cells.[19] Moreover, in a study with allergic rhinosinusitis, the baseline salivary CGRP levels were elevated in between attacks.[20] Intranasal capsaicin application has been shown to reduce polyp recurrence following surgery and decrease the intensity of nasal obstruction in CRS.[21] In a preclinical study in pig nasal mucosa, CGRP antagonists attenuated capsaicin-, histamine-, and bradykinin-induced vasodilation.[22] In another study, $CGRP_{8-37}$ attenuated the capsaicin-induced vasodilation and decrease in nasal cavity volume which was unaffected by NK1-antagonists. This suggests that CGRP, but not substance P, is an important mediator of nasal vascular changes resulting from activation of C-fiber afferents.[23]

BHV-3000 (PF-07899801) (rimegepant) is a calcitonin gene-related peptide (CGRP) receptor antagonist in development for the acute treatment of CRS. The CGRP receptor is located within pain-signaling pathways, intracranial arteries and the trigeminal ganglion, and its activation is thought to play a causal role in CRS pathophysiology.

This study is being conducted to evaluate the efficacy, safety, and tolerability of rimegepant for the acute treatment of CRS. It will also further define the safety profile of rimegepant administration in this patient population, separately from the approved indication of acute and preventive treatment of migraine.

1.3.1 Study Design Rationale

This is a double-blind, randomized, multicenter, outpatient evaluation of the safety and efficacy of rimegepant as compared to placebo in the acute treatment of chronic rhinosinusitis (CRS) with or without polyps. The study drug will be rimegepant formulated in a 75 mg ODT (placed on or under the tongue) or a matching placebo. The subjects will be instructed to take their study medication, as an outpatient, when (if) they have a facial pain/pressure/fullness which reaches intensity of ≥ 6 on the NRS (0-10).

The study will randomize approximately 286 subjects to result in approximately 200 evaluable subjects. The subjects will be randomized in a 1:1 ratio to the rimegepant or placebo treatment groups. The randomization will be stratified by the presence of nasal polyps (yes or no).

090177e19e292258\Approved\Approved On: 28-Jul-2023 15:57 (GMT)

1.3.2 Dose Selection Rationale

The Phase 2b dose-ranging study, CN170003, established that rimegepant 75 mg is the minimum effective dose for the acute treatment of migraine. The three Phase 3 studies, BHV3000-301, BHV3000-302, and BHV3000-303, confirmed this efficacy using the current registrational endpoints for acute treatment of migraine. This observation and the flat dose-response with rimegepant and other CGRP receptor antagonists suggest that rimegepant 75 mg may be an effective dose for the acute treatment of chronic rhinosinusitis with or without polyps. The pharmacokinetic profile of rimegepant supports this study's administration of a single dose. Please refer to the current version of Investigator's Brochure for a summary of the clinical safety profile.

1.3.3 Research Hypothesis

Rimegepant will have efficacy superior to placebo in the acute treatment of chronic rhinosinusitis (CRS) with or without polyps with a favorable safety profile.

1.3.4 Benefit/Risk

Rimegepant represents an advancement in migraine therapeutics, providing the first CGRP antagonist to demonstrate benefit for both the acute treatment and prophylaxis of migraine.

Rimegepant appears to be generally safe and well tolerated in humans when given as single oral doses from 75 mg up to the maximum dose of 1500 mg and multiple oral doses up to the maximum daily dose of 600 mg for 14 days.

Broad and sustained efficacy of rimegepant 75 mg was demonstrated in 3 previously completed Phase 3 studies (BHV3000-301, BHV3000-302, and BHV3000-303). Statistically significant efficacy was demonstrated on the co-primary endpoints of freedom from pain, and freedom from most bothersome symptom at 2 hours post-dose. Similar results were demonstrated in the BHV3000-310 study recently completed in China and Korea. In the Phase 2/3 placebo-controlled study (BHV3000-305) for the preventive treatment of migraine, rimegepant at a dose of 75 mg every other day (EOD) demonstrated statistically significant superiority to placebo on the primary endpoint of change from the observation period in the mean number of migraine days per month on treatment in the last month of the double-blind treatment phase.

A multicenter open-label, long-term study (BHV3000-201) was conducted to evaluate the safety and tolerability of rimegepant 75 mg tablet taken as needed (up to one tablet per day upon onset of a migraine of mild, moderate, or severe intensity) for the acute treatment of migraine for up to 52 weeks. This multiple-dose, long-term study of rimegepant 75 mg administered for up to 52 weeks confirmed the favorable safety profile across a variety of safety endpoints, including AE assessments, clinical laboratory testing including liver function tests (LFTs), vital signs and electrocardiograms (ECGs). Safety data from the double-blind treatment and the open-label extension phases of the pivotal Phase 2/3, randomized, double-blind, placebo-controlled preventive treatment of migraine study (BHV3000-305) support a favorable safety profile of rimegepant 75 mg administered EOD for the preventive treatment of migraine. Rimegepant 75

090177e19e292258\Approved\Approved On: 28-Jul-2023 15:57 (GMT)

mg administered EOD + PRN for up to 52 weeks in the open-label phase is well tolerated, with no new safety signals observed in the open-label-extension phase.

Across rimegepant clinical development program, low frequency of events of hypersensitivity (including urticaria, angioedema, anaphylactic reaction and rash) were observed. No AEs representing serious cutaneous manifestation of hypersensitivity (e.g., Stevens-Johnson syndrome) were observed.

Across the rimegepant clinical development program, no cases of Hy's Law were identified, and there was no signal of DILI due to rimegepant when administered up to once daily PRN for up to 52 weeks of treatment.

There are no adequate data on the developmental risk associated with the use of rimegepant in pregnant women. Both women of childbearing potential, as well as those who are of non-childbearing potential, may be enrolled given the availability of embryo-fetal development (EFD) nonclinical toxicity studies with rimegepant. Contraception method is required, and measures will be taken to limit the risk of pregnancy in the female population of childbearing potential enrolled (See Section 16.3). The potential risk of exposure to rimegepant in a sexual partner of a male subjects in this study via ejaculate is low, and therefore no contraception (condom) use in male subjects is warranted. The calculated safety margin is ≥100-fold between the estimated partner exposure due to seminal transfer and the NOAEL for serious manifestations of developmental toxicity in nonclinical studies. The safety margin of 100-fold is based on applying a 10-fold safety factor for interspecies extrapolation and a 10-fold safety factor for susceptible populations.[28]

Subjects undergo regular pregnancy testing throughout the duration of the study. Although no safety issues in clinical trials of rimegepant were observed, cardiovascular events, cerebrovascular events, hypertensive events, and serious gastrointestinal events associated with constipation are reviewed in each aggregate report per The United States Food and Drug Administration (FDA) request. None of these reviews have detected any safety signal associated with these events. Subjects are excluded if there is uncontrolled, unstable, or recently diagnosed cardiovascular disease or hypertension. Subjects are monitored through multiple safety endpoints, including AE assessments, clinical laboratory testing, vital signs and ECGs.

Review of all data available, including post-marketing information, nonclinical, clinical, and scientific literature data, demonstrates a favorable benefit-risk profile for the use of rimegepant in this study. More detailed information about the known and expected benefits and risks and reasonably expected AEs of rimegepant may be found in the Investigator Brochure, which is the SRSD for this study.

Investigators are to monitor changes in hematology, chemistry, and other laboratory measures (see Section 8.5). In addition, Investigators are required to monitor all reported AEs, changes on physical examination, ECG, and emergent lab abnormalities, etc. (see Table 1).

090177e19e292258\Approved\Approved On: 28-Jul-2023 15:57 (GMT)

2 STUDY OBJECTIVES

2.1 Primary Objective(s)

To evaluate the efficacy of rimegepant compared with placebo in the acute treatment of chronic rhinosinusitis with or without polyps on mean change from baseline of facial pain/pressure/fullness on NRS (0-10) score at 2 hours post dose.

2.2 Secondary Objective(s)

1. To evaluate rimegepant compared to placebo on change from baseline in Total Nasal Symptom Score (TNSS) at 2 hours post-dose.
2. To evaluate rimegepant compared to placebo on change from baseline in nasal obstruction (congestion) score on NRS (0-10) score at 2 hours post-dose.
3. To evaluate rimegepant compared to placebo on change from baseline in nasal discharge score on NRS (0-10) score at 2 hours post-dose.
4. To evaluate rimegepant compared to placebo on headache pain relief on a 4-point Likert scale at 2 hours post-dose compared to baseline.
5. To evaluate rimegepant compared to placebo on the probability of requiring rescue medication within 24 hours of initial treatment.

2.3 Exploratory Objective(s)

1. To evaluate rimegepant compared to placebo on ≥ 30% reduction from baseline of facial pain/pressure/fullness NRS (0-10) score at 2 hours.
2. To evaluate rimegepant compared to placebo on ≥ 50% reduction from baseline of facial pain/pressure/fullness NRS (0-10) score at 2 hours.
3. To evaluate the effect of rimegepant compared to placebo on change from baseline of facial pain/pressure/fullness NRS (0-10) score at 15, 30, 45, 60, 90 minutes and 2, 4, 8 and 24 hours post-dose.
4. To evaluate the effect of rimegepant compared to placebo on change from baseline of nasal obstruction (congestion) NRS (0-10) score at 15, 30, 45, 60, 90 minutes and 2, 4, 8 and 24 hours post-dose.
5. To evaluate the effect of rimegepant compared to placebo on change from baseline of nasal discharge NRS (0-10) score at 15, 30, 45, 60, 90 minutes and 2, 4, 8 and 24 hours post-dose.

090177e19e292258\Approved\Approved On: 28-Jul-2023 15:57 (GMT)

6. To evaluate the effect of rimegepant compared to placebo on headache pain relief using a 4-point Likert scale at 15, 30, 45, 60, 90 minutes and 2, 4, 8 and 24 hours post-dose compared to baseline.

7. To evaluate rimegepant compared to placebo on the PGI-C questionnaire at 24 hours post-dose.

8. To evaluate rimegepant compared to placebo on ≥ 30% reduction from baseline of facial pain/pressure/fullness NRS (0-10) score at 24 hours.

9. To evaluate rimegepant compared to placebo on ≥ 50% reduction from baseline of facial pain/pressure/fullness NRS (0-10) score at 24 hours.

10. To evaluate rimegepant compared to placebo on sustained relief of facial pain/pressure/fullness, as defined by each of the following: 30% reduction from baseline on NRS (0-10), 1.5-point reduction on NRS (0-10), and a 2-point reduction on NRS (0-10) through 24 hours post-dose.

11. To explore the distribution of baseline disease severity using the of Sino-Nasal Outcome Test (SNOT-22).

12. To evaluate rimegepant compared to placebo on the Sino-Nasal Outcome Test (SNOT-22) score at 24 hours post-dose.

13. To evaluate the safety and tolerability of rimegepant in the acute treatment of chronic rhinosinusitis as measured by the frequency of adverse events of moderate or severe intensity, serious adverse events (SAEs), clinically relevant laboratory abnormalities, and nasal inspection abnormalities.

14. To evaluate rimegepant compared to placebo for the Columbia-Suicide Severity Rating Scale (C-SSRS).

090177e19e292258\Approved\Approved On: 28-Jul-2023 15:57 (GMT)

3 STUDY ENDPOINTS

3.1 Primary Endpoint(s)

Change from baseline facial pain/pressure/fullness will be assessed using the number of evaluable subjects that report facial pain/pressure/fullness ≥ 6 on NRS (0-10) at baseline and who also report pain level at 2 hours post-dose recorded in the eDiary.

3.2 Secondary Endpoint(s)

1. Change from baseline of **Total Nasal Symptom Score (TNSS)** will be assessed using the number of evaluable subjects that report facial pain/pressure/fullness, nasal obstruction (congestion), and nasal discharge scores at baseline and at 2 hours post-dose in the eDiary.

2. Change from baseline of **nasal obstruction (congestion)** will be assessed using the number of evaluable subjects that report nasal obstruction (congestion) score on NRS (0-10) score at baseline and 2 hours post-dose in the eDiary.

3. Change from baseline of **nasal discharge** will be assessed using the number of evaluable subjects that report nasal discharge score on NRS (0-10) score at baseline and 2 hours post-dose in the eDiary.

4. **Headache pain relief** will be assessed using the number of evaluable subjects that report a headache pain level of moderate or severe intensity at baseline and then report a pain level of none or mild at 2 hours post-dose in the eDiary using a 4-point Likert scale.

5. The **probability of requiring rescue medication** will be assessed using the number of subjects that take rescue medication within 24 hours after administration of study medication (rimegepant or placebo).

3.3 Measures of interest

Safety and Other assessments:

- Adverse Events
- ECG assessments
- Vital Signs and Physical Measurements
- Routine Laboratory Tests
- Columbia-Suicide Severity Rating Scale (C-SSRS)
- Assessment of CRS Pain and Symptoms
- Patient Global Impression of Change (PGI-C)
- Sino-Nasal Outcome Test (SNOT-22)

090177e19e292258\Approved\Approved On: 28-Jul-2023 15:57 (GMT)

4 STUDY PLAN

4.1 Study Design and Duration

This is a double-blind, randomized, multicenter, outpatient evaluation of the safety and efficacy of rimegepant as compared to placebo in the acute treatment of chronic rhinosinusitis with or without polyps. Subjects will be dispensed one dose of study medication consisting of a rimegepant 75 mg ODT (placed on or under the tongue) or a matching placebo. The total duration of subject's study participation will be up to approximately 66 days. This includes a 3 to 14 day (+2 days for scheduling purposes, if needed) Screening Period, an Acute Treatment Phase that can last up to 45 days or until the subject has a facial pain/pressure/fullness that reaches a current intensity of ≥ 6 on the NRS (0-10), followed by an End of Treatment Visit (EOT) within 7 days (+2 days for scheduling purposes, if needed) after the administration of the study medication.

If a subject has NOT experienced a facial pain/pressure/fullness of sufficient severity within 45 days after randomization, they still are required to complete all EOT visit procedures.

All subjects must return empty medication containers or unused study medication and the eDiary to the study center.

The end of the study is defined as the date of the last visit of the last subject.

090177e19e292258\Approved\Approved On: 28-Jul-2023 15:57 (GMT)

090177e19e292258\Approved\Approved On: 28-Jul-2023 15:57 (GMT)

4.2 Schedule of Assessments

Table 1 BHV3000-316 (C4951015) Schedule of Assessments

Procedure	Screening Visit (3-14 days)	Baseline Visit (Randomization)	Weekly between Baseline Visit and Dosing	Facial pain/pressure/fullness ≥ 6 on NRS (0-10)	During Treatment 15, 30, 45, 60 and 90 minutes Post-Dose	During Treatment 2, 4, 8 hours Post-Dose	During Treatment 24 hours Post-Dose	End of Treatment Visit (within 7 (+2) days of treating facial pain/pressure/fullness)	Comments
**Eligibility Assessments**									
Informed Consent	X								
Inclusion/ Exclusion Criteria	X	X							
Medical History	X								
Collect Daily Use of Medications to Reduce Intensity of CRS Symptoms /Concomitant Medications	X	X						X	Subjects will be given paper diaries to use to keep track of their concomitant medications and rescue medications throughout the study. All medications taken for any reason should be documented. Site staff should instruct subjects on proper use of the paper diaries and instruct subjects that the diaries will be collected, reviewed, and discussed with the subject at each

090177e19e292258\Approved\Approved On: 28-Jul-2023 15:57 (GMT)

Table 1 BHV3000-316 (C4951015) Schedule of Assessments

Procedure	Screening Visit (3-14 days)	Baseline Visit (Randomization)	Weekly between Baseline Visit and Dosing	Facial pain/pressure/fullness ≥ 6 on NRS (0-10)	During Treatment 15, 30, 45, 60 and 90 minutes Post-Dose	During Treatment 2, 4, 8 hours Post-Dose	During Treatment 24 hours Post-Dose	End of Treatment Visit (within 7 (+2) days of treating facial pain/pressure/fullness)	Comments
									visit.
Assessment of CRS History)	X								**Paper source document** will be used to capture CRS History and entered in eCRF
**Safety Assessments**									
Physical Examination	X							X	
Vital Signs/Physical Measurements	X	X						X	Vital Signs consist of sitting arterial systolic and diastolic blood pressure, heart rate, oral body temperature, body weight and height. Height and weight will only be measured at Screening.
Clinical Safety Laboratory Testing	X							X	All Screening visit laboratory test results must be received prior to Baseline Visit (randomization)

090177e19e292258\Approved\Approved On: 28-Jul-2023 15:57 (GMT)

Table 1 BHV3000-316 (C4951015) Schedule of Assessments

Procedure	Screening Visit (3-14 days)	Baseline Visit (Randomization)	Weekly between Baseline Visit and Dosing	Facial pain/pressure/fullness ≥ 6 on NRS (0-10)	During Treatment 15, 30, 45, 60 and 90 minutes Post-Dose	During Treatment 2, 4, 8 hours Post-Dose	During Treatment 24 hours Post-Dose	End of Treatment Visit (within 7 (+2) days of treating facial pain/pressure/fullness)	Comments
FSH	X								FSH levels will be measured on post-menopausal women to confirm post-menopausal status. FSH level testing is not required for women greater than or equal to 62 years old with amenorrhea of greater than or equal to 1 year.
HbA1c	X								
Lipid Panel	X								Triglycerides, total cholesterol, HDL, and LDL
Urine drug screen for drugs of abuse	X							X	
ECG	X							X	
Pregnancy Test	X	X		X				X	A serum pregnancy test will be completed at Screening and End of Treatment Visits as part of the standard laboratory tests (if appropriate). Confirmatory urine pregnancy test for

090177e19e292258\Approved\Approved On: 28-Jul-2023 15:57 (GMT)

Table 1 BHV3000-316 (C4951015) Schedule of Assessments

Procedure	Screening Visit (3-14 days)	Baseline Visit (Randomization)	Weekly between Baseline Visit and Dosing	Facial pain/pressure/fullness ≥ 6 on NRS (0-10)	During Treatment 15, 30, 45, 60 and 90 minutes Post-Dose	During Treatment 2, 4, 8 hours Post-Dose	During Treatment 24 hours Post-Dose	End of Treatment Visit (within 7 (+2) days of treating facial pain/pressure/fullness)	Comments
									WOCBP should be completed on site at the Baseline Visit and any subsequent visits for confirmation at the Investigator's discretion. Home pregnancy test will be provided to WOCBP after completion of baseline visit. WOCBP subjects must complete the urine pregnancy test at home, and that test result must be negative prior to taking study medication.
Adverse Event and Serious Adverse Event Assessment	X	X		X	X	X	X	X	SAEs and non-serious AEs are reported from the time of informed consent. See Section 8 of the protocol for further information.

Table 1 BHV3000-316 (C4951015) Schedule of Assessments

Procedure	Screening Visit (3-14 days)	Baseline Visit (Randomization)	Weekly between Baseline Visit and Dosing	Facial pain/pressure/fullness ≥ 6 on NRS (0-10)	During Treatment 15, 30, 45, 60 and 90 minutes Post-Dose	During Treatment 2, 4, 8 hours Post-Dose	During Treatment 24 hours Post-Dose	End of Treatment Visit (within 7 (+2) days of treating facial pain/pressure/fullness)	Comments
Columbia-Suicide Severity Rating Scale (C-SSRS)	X	X						X	This scale will be clinician administered, completed on site, and will be in paper. The source document will be provided by Pfizer. See Section 6.2.5 for further details.
**Clinical Drug Supplies/Study Supplies**									
Randomize		X							Subjects will be randomized in the IWRS system at the Baseline Visit (randomization)
Dispense Study Medication		X							
Weekly Contact (e.g. via phone, text, email)			X						Subjects should be contacted at least weekly and questioned about their systemic corticosteroid and antibiotics (other than topical nasal antibiotics) use and removed from the study according to

090177e19e292258\Approved\Approved On: 28-Jul-2023 15:57 (GMT)

090177e19e292258\Approved\Approved On: 28-Jul-2023 15:57 (GMT)

Table 1 BHV3000-316 (C4951015) Schedule of Assessments

Procedure	Screening Visit (3-14 days)	Baseline Visit (Randomization)	Weekly between Baseline Visit and Dosing	Facial pain/pressure/fullness ≥ 6 on NRS (0-10)	During Treatment 15, 30, 45, 60 and 90 minutes Post-Dose	During Treatment 2, 4, 8 hours Post-Dose	During Treatment 24 hours Post-Dose	End of Treatment Visit (within 7 (+2) days of treating facial pain/pressure/fullness)	Comments
									the requirements in Section 6.2.6.
Administer 1 dose of study medication				X					Subjects will use eDiary to answer questions about their CRS symptoms upon experiencing a facial pain/pressure/fullness of ≥ 6 on NRS (0-10). The subject will administer pre-dispensed study drug if 1) the facial pain/pressure/fullness remains ≥ 6 on NRS (0-10); 2) the subject has completed all required CRS assessment questions in the eDiary, and 3) *the subject has not already taken prohibited medications (see protocol Section 5.4)*.

Table 1 BHV3000-316 (C4951015) Schedule of Assessments

Procedure	Screening Visit (3-14 days)	Baseline Visit (Randomization)	Weekly between Baseline Visit and Dosing	Facial pain/pressure/fullness ≥ 6 on NRS (0-10)	During Treatment 15, 30, 45, 60 and 90 minutes Post-Dose	During Treatment 2, 4, 8 hours Post-Dose	During Treatment 24 hours Post-Dose	End of Treatment Visit (within 7 (+2) days of treating facial pain/pressure/fullness)	Comments
									**The eDiary will prompt the subject to take study medication.**
Return unused study medication								X	
eDiary returned/ reviewed for completeness								X	Staff to review and confirm eDiary entries with subjects, confirm all data points are transferred to the system, and reset eDiary for future subject use, PRIOR to the subject leaving the clinic

090177e19e292258\Approved\Approved On: 28-Jul-2023 15:57 (GMT)

090177e19e292258\Approved\Approved On: 28-Jul-2023 15:57 (GMT)

Table 1 BHV3000-316 (C4951015) Schedule of Assessments

Procedure	Screening Visit (3-14 days)	Baseline Visit (Randomization)	Weekly between Baseline Visit and Dosing	Facial pain/pressure/fullness ≥ 6 on NRS (0-10)	During Treatment 15, 30, 45, 60 and 90 minutes Post-Dose	During Treatment 2, 4, 8 hours Post-Dose	During Treatment 24 hours Post-Dose	End of Treatment Visit (within 7 (+2) days of treating facial pain/pressure/fullness)	Comments
**Efficacy Assessments**									±windows for timeframe around efficacy assessments (15, 30, 45, 60, 90 min, 2, 4, 8, and 24 hours) will be automated and captured in the eDiary.
Assessment of facial pain/pressure/ fullness				X	X	X	X		This scale is captured in the eDiary
Assessment of nasal obstruction (congestion)				X	X	X	X		This scale is captured in the eDiary.
Assessment of nasal discharge				X	X	X	X		This scale is captured in the eDiary
Headache Pain				X	X	X	X		This scale is captured in the eDiary for subjects who report current headache pain of moderate or severe at the time of qualifying facial

Table 1 BHV3000-316 (C4951015) Schedule of Assessments

Procedure	Screening Visit (3-14 days)	Baseline Visit (Randomization)	Weekly between Baseline Visit and Dosing	Facial pain/pressure/fullness ≥ 6 on NRS (0-10)	During Treatment 15, 30, 45, 60 and 90 minutes Post-Dose	During Treatment 2, 4, 8 hours Post-Dose	During Treatment 24 hours Post-Dose	End of Treatment Visit (within 7 (+2) days of treating facial pain/pressure/fullness)	Comments
									pain/pressure/fullness
Patient Global Impression of Change (PGI-C)							X		This scale is captured in the eDiary
Sino-Nasal Outcome Test (SNOT-22)		X					X		This scale is captured on paper at the Baseline Visit and in the eDiary at 24-hours post-dose.

090177e19e292258\Approved\Approved On: 28-Jul-2023 15:57 (GMT)

4.2.1 Screening Phase (3 to 14 days)

The Screening Phase will be 3 to 14 days (+2 days if needed for scheduling purposes). All subjects who signed Informed Consent will be entered into the Interactive Web Response System (IWRS) and assigned a subject identifier. After obtaining informed consent, subjects will undergo all screening procedures as detailed in Table 1. After all screening procedures are complete, the subject meets the Inclusion/Exclusion Criteria and lab test results have been reviewed by the Investigator, the Baseline Visit will be scheduled.

Subjects who were considered screen failures may be considered for re-screening provided the ineligibility was due to one of the eligibility criteria that may have changed due to medical intervention or one of the eligibility criteria modified in a protocol amendment. Adequate documentation in source records must support the previously failed criteria. In all possible re-screening circumstances, the situation must be discussed with the sponsor prior to re-screening, with approval in writing from the sponsor prior to re-screening. If a subject is approved for re-screening, a new subject number must be obtained from the appropriate study-related system. Re-screening will only be permitted one time.

4.2.2 Acute Treatment (Randomization) Phase

It is estimated that approximately 286 subjects will be randomized to result in approximately 200 evaluable subjects. If the subject meets all eligibility criteria, they will be randomized at the Baseline Visit via the IWRS. Subjects will be provided with an eDiary. Study personnel will educate the subject on the proper use of the eDiary, accurate reporting and placebo response prior to the subject leaving the office.

After randomization via the IWRS, the subject will be dispensed a single dose of the double-blind study medication to take home. The subject will be instructed to take their study medication within 45 days, as an outpatient, when (if) they have facial pain/pressure/fullness which reaches a current intensity of ≥ 6 on the NRS (0-10) ***and after*** they answer eDiary questions about their current pain and symptoms. The subject will complete questionnaires in their eDiary, at various timepoints, for 24 hours after taking study medication to record efficacy and other quality of life measures.

Subjects in this study may be randomized only once. Under no circumstances may a subject be re-randomized.

4.2.2.1 eDiary Data Collection

Subjects will record the date and time of dosing, pre-dose and post-dose efficacy assessments, and other quality of life measures in their eDiary. Subjects should not dose with study medication until the facial pain/pressure/fullness reaches a current intensity of ≥ 6 on the NRS (0-10) **and** they have completed the pre-dose assessments in the eDiary. Efficacy and quality of life measurements include the following: onset time of facial pain/pressure/fullness at time of taking study medication and after dosing at times listed in Table 1. Nasal obstruction

090177e19e292258\Approved\Approved On: 28-Jul-2023 15:57 (GMT)

(congestion) severity and Nasal discharge severity and will be recorded using a NRS (0-10) at the onset of the facial pain/pressure/fullness ≥ 6 on the NRS (0-10) and after dosing at times listed in Table 1. Headache pain severity will be recorded using a 4-point Likert scale (0 = None; 1 = Mild; 2 = Moderate; 3 = Severe) at the onset of the facial pain/pressure/fullness ≥ 6 on the NRS (0-10) and after dosing at times listed in Table 1.

The Patient Global Impression of Change (PGI-C) will be completed by the subject 24 hours after dosing. The Sino-Nasal Outcome Test (SNOT-22) questionnaire will be completed on paper at the Baseline Visit and on the eDiary at 24 hours after dosing.

After dosing with study medication, all other medication to treat CRS symptoms is prohibited during the 2 hours post-dose. At the end of 2 hours after dosing with study medication (and after the 2-hour assessments have been completed on the eDiary) subjects will be permitted to use the following rescue medication: acetaminophen (up to 1000 mg/day), or aspirin, ibuprofen, naproxen (or any other type of nonsteroidal anti-inflammatory drug [NSAID]), oral antihistamines (non-sedating), oral decongestants, topical nasal decongestants, topical nasal anticholinergics (all up to the daily recommended dose indicated on the drug packaging). These are the only medications allowed for rescue treatment after 2 hours post-dose of study medication. However, if needed, after 24 hours of administering the one dose of study medication (and before returning for the End of Treatment Visit) subject may take their prescribed standard of care medications for treatment of CRS symptoms provided all the assessments have been completed on the eDiary. Exclusionary rescue medication such as, opioids and butalbital compounds, are not allowed on this study. Similarly, if the facial pain/pressure/fullness is relieved by study medication at 2 hours after dosing but then recurs to intensity level of ≥ 6 on the NRS (0-10) between 2 and 24 hours, the subject will be permitted to take the same rescue therapy as outlined above.

Subjects should be encouraged to treat the first qualifying facial pain/pressure/fullness (intensity level of ≥ 6 on the NRS (0-10)) that occurs during the treatment phase. If subjects are unable to treat their first qualifying facial pain/pressure/fullness due to scheduling, etc., the same medication restrictions would still apply (i.e., subjects would only be permitted to take acetaminophen (up to 1000 mg/day), or aspirin, ibuprofen, naproxen (or any other type of nonsteroidal anti-inflammatory drug [NSAID]), oral antihistamines (non-sedating), oral decongestants, topical nasal decongestants, topical nasal anticholinergics [all up to the daily recommended dose indicated on the drug packaging]).

Similarly, for treatment of non-qualifying facial pain/pressure/fullness (i.e., intensity level of < 6 on the NRS (0-10)) that occur during the randomization period before a qualifying facial pain/pressure/fullness is reported, subject will only be permitted to use the medications listed above. Acetaminophen (over 1000 mg/day) is prohibited after randomization except as rescue medication as described in Section 5.5. In all circumstances, the subject will continue to complete his or her eDiary for up to 24 hours after consuming the study medication.

090177e19e292258\Approved\Approved On: 28-Jul-2023 15:57 (GMT)

Subjects should have access to systemic corticosteroids and antibiotics during the trial for treatment of symptoms if indicated. However, subjects should discontinue the study if they receive those therapies.

4.2.3 Extension Phase

Not applicable.

4.2.4 Washout Phase

Not applicable.

4.2.5 End of Treatment

Subjects will return to the study site within 7 days of study treatment (+2 days) for review of the eDiary assessment, of medication compliance, and monitoring of tolerability and safety (including vital signs, laboratory tests, and electrocardiography). If a subject has NOT experienced a facial pain/pressure/fullness of sufficient severity within 45 days after randomization, they still are required to complete all EOT visit procedures. All subjects must return unused study medication or empty packaging and the eDiary to the study center.

4.3 Post Study Access to Therapy

At the end of the study the sponsor will not continue to supply study drug to subjects/Investigators. The Investigator should ensure that the subject receives the appropriate standard of care to treat the condition under study.

090177e19e292258\Approved\Approved On: 28-Jul-2023 15:57 (GMT)

5 POPULATION

Individuals entered in this trial will be subjects who suffer from chronic rhinosinusitis with or without nasal polyps as specified under Section 5.2. The treatment setting for these subjects may include clinics, institutions, or private office practices. Subjects may be recruited through a variety of sources, including referral from physicians and other health care professionals.

5.1 Number of Subjects

The study will randomize approximately 286 subjects to result in approximately 200 evaluable subjects.. The subjects will be randomized in a 1:1 ratio to the rimegepant or placebo treatment groups.

5.2 Inclusion Criteria

1. Target population

   At the Screening Visit, the subject has a **minimum 3-month history** of chronic rhinosinusitis (CRS) with or without polyps, diagnosed by a healthcare provider, including all of the following:

   a. **Facial pain/pressure/fullness AND** one or more of the following signs and symptoms: nasal discharge (anterior, posterior, or both), nasal obstruction (congestion), or decreased sense of smell.

   b. **Inflammation is documented** by at least one of these three findings:

      i. Endoscopic findings of mucopurulent drainage (not clear) or edema in the middle or superior meatus, or anterior ethmoid region

      ii. Polyps in nasal cavity or the middle or superior meatus

      iii Radiographic imaging showing inflammation of the paranasal sinuses with partial opacification of at least two sinuses or complete opacification of at least one sinus.

      Results from historic imaging, diagnostic or historic endoscopy before the Screening Visit, but not older than 12 months, can be considered for confirmation of inflammation.

   c. At least two episodes of facial pain/pressure/fullness of moderate or severe intensity on a 4-point rating scale (0 = None, 1 = Mild, 2 = Moderate, 3 = Severe) in the past 30 days prior to the Screening Visit.

   d. Subject with daily use of medications listed in Section 5.5 is permitted to remain on therapy if they have been on a stable dose for at least 1 month prior to the Screening Visit, and the dose is not expected to change during the course of the study.

090177e19e292258\Approved\Approved On: 28-Jul-2023 15:57 (GMT)

e. Subject agrees to not commence new therapy or any pain management techniques to treat their CRS during the course of the study.

2. Age and Reproductive Status

   a. Male and Female subjects ≥ 18 years and older.

   b. Subject meets reproductive criteria. Refer to Section 16.3 for reproductive criteria for male (Section 16.3.1) and female (Section 16.3.2) subjects.

   c. At the Baseline Visit prior to dispensing Investigational Study Medication, WOCBP must have a negative urine pregnancy test.

3. No clinically significant abnormality identified on the medical or laboratory evaluation. A subject with a clinical abnormality or laboratory parameters outside the reference range may be included only if the Investigator considers that the finding is not clinically significant and will not introduce additional risk factors and will not interfere with the study procedures.

5.3 Exclusion Criteria

1. Disease Target Exclusion

   a. Subject has a history of primary headache disorder (e.g., migraine, tension-type headache, cluster headache), or MOH, diagnosed by a healthcare provider, consistent with diagnostic criteria according to the International Classification of Headache Disorders, 3rd Edition (ICHD-3). Infrequent tension-type headache consistent with diagnostic criteria according to the ICHD-3 is not exclusionary.

   b. Subject has a history of nasal or facial surgery within the 6 months prior to the Screening Visit.

   c. Subject has ongoing rhinitis medicamentosa (rebound congestion).

   d. Subject has a history of nasal or upper respiratory infection within the 2 weeks prior to the Screening Visit.

   e. Subject has a history of invasive fungal rhinosinusitis.

   f. Subject has diagnosed acute bacterial/viral rhinosinusitis or has symptoms suggestive of an acute exacerbation of their chronic rhinosinusitis (e.g., coughing, fever, purulent discharge) within the 2 weeks prior to the Screening Visit. If the subject has diagnosed acute bacterial/viral rhinosinusitis or has symptoms suggestive of an acute exacerbation of their chronic rhinosinusitis between screening and dosing with study drug, the subject should be discontinued from the study.

   g. Subject has a diagnosis of cystic fibrosis.

090177e19e292258\Approved\Approved On: 28-Jul-2023 15:57 (GMT)

h. Subject has a diagnosis of primary ciliary dyskinesia (immotile-cilia syndrome).

i. Subject is currently receiving aspirin desensitization or maintenance therapy for Samter's Triad.

j. Subject has a history of hypogammaglobulinemia.

k. Subject has a history of immunodeficiency syndrome.

l. Subject has a history of granulomatous diseases.

m. Subject has a history of recurrent acute sinusitis (four or more episodes per year of acute bacterial rhinosinusitis (ABRS) without signs or symptoms of rhinosinusitis between episodes).

n. Subject has a history of sinus mucocele within 1 year prior to the Screening Visit.

o. Subject has a history of malignant nasal or paranasal tumor.

p. Subject has a history of odontogenic sinusitis within 1 year prior to the Screening Visit.

2. Medical History and Concurrent Diseases

a. Body Mass Index > 35.0 kg/m^2

b. Subject history with current evidence of uncontrolled, unstable or recently diagnosed cardiovascular disease, such as ischemic heart disease, coronary artery vasospasm, and cerebral ischemia. Subjects with Myocardial Infarction (MI), Acute Coronary Syndrome (ACS), Percutaneous Coronary Intervention (PCI), cardiac surgery, stroke or transient ischemic attack (TIA) during the 6 months prior to the Screening Visit.

c. Subject has uncontrolled hypertension (high blood pressure), or uncontrolled diabetes (however subjects can be included who have stable hypertension and/or diabetes for at least 3 months prior to the Screening Visit). A single blood pressure measurement of greater than 150 mm Hg systolic or 100 mm Hg diastolic after 10 minutes of rest is exclusionary.

d. Subject has a current diagnosis of major depression, other pain syndromes, psychiatric conditions (e.g., schizophrenia), dementia, or significant neurological disorders that, in the Investigator's opinion, might interfere with study assessments.

e. Subject has a history of gastric, or small intestinal surgery (including Gastric Bypass, Gastric Banding, Gastric Sleeve, Gastric Balloon, etc.), or has a disease that causes malabsorption.

f. Subject has a history or current evidence of any significant and/or unstable medical conditions (e.g., history of congenital heart disease or arrhythmia, known suspected

090177e19e292258\Approved\Approved On: 28-Jul-2023 15:57 (GMT)

infection, hepatitis B or C, or cancer) that, in the Investigator's opinion, would expose them to undue risk of a significant adverse event (AE) or interfere with assessments of safety or efficacy during the course of the trial. Severe hepatic impairment (Child-Pugh C) is exclusionary in all cases.

g. Subject has a history of, treatment for, or evidence of, alcohol or drug abuse or who have met DSM-V criteria[24] for any significant substance use disorder within the 12 months prior to the Screening Visit.

h. Subject should be excluded if they have a positive drug screen for drugs of abuse that in the Investigator's judgment is medically significant, in that it would impact the safety of the subject or the interpretation of the study results. In addition:

   i. Detectable levels of cocaine, amphetamine, and phencyclidine (PCP) in the drug screen are exclusionary. Subjects who are positive for amphetamines, and who are on a prescribed amphetamine medication for an approved indication (e.g., ADHD) will be allowed into the study at the Investigator's discretion. This determination by the Investigator must be well documented in the subject's source medical records. The stimulant dose must be stable from 3 months prior to the Screening Visit until the end of treatment visit occurs.

   ii. Detectable levels of cannabinoids (e.g., marijuana, CBD) in the drug screen at the Screening Visit are not exclusionary, if in the Investigator's documented opinion the subject does not meet DSM-V criteria[24] for substance use disorder, and the positive test does not signal a clinical condition that would impact the safety of the subject or interpretation of the study results.

3. Allergies and Adverse Drug Reactions

   a. Subject has a history of drug or other allergy which, in the opinion of the Investigator, makes the subject unsuitable for participation in the study.

4. Sex and Reproductive Status

   a. Females of child-bearing potential who are unwilling or unable to use an acceptable contraceptive method or abstinence to avoid pregnancy for the entire study period and for 28 days after dosing with study drug.

   b. Women who are pregnant, lactating or breastfeeding.

   c. Women with a positive pregnancy test on enrollment or prior to study drug administration.

090177e19e292258\Approved\Approved On: 28-Jul-2023 15:57 (GMT)

5. ECG and Laboratory Test Findings

    a. Estimated glomerular filtration rate (eGFR) according to the re-expressed abbreviated (four-variable) Modification of Diet in Renal Disease (MDRD) Study equation < 30 ml/min/1.73m^2

    b. ECG abnormalities that, in the Investigator's opinion, makes the subject unsuitable for participation in the study.

    c. Total serum bilirubin > 1.5 x ULN (Only abnormal values with clinical justification ***may be repeated once for confirmation during the screening period)***. For Gilbert's syndrome, direct bilirubin > 1 x ULN is exclusionary.

    d. Neutrophil count ≤ 1000/μL (or equivalent).

    e. HbA1c > 7.5%

    f. AST (SGOT) or ALT (SGPT) > 2 x ULN (Only abnormal values with clinical justification ***may be repeated once for confirmation during the screening period***).

6. Excluded Current or Recent Treatments

    a. Subject has a history of antibiotics (other than topical nasal antibiotics) use within the 14 days prior to Screening Visit.

    b. Subject has a history of systemic corticosteroids use within the 30 days prior to Screening Visit.

    c. Subject has a history of corticosteroid eluting sinus implants within the 3 months of date of implant (PROPEL®) or 6 months of date of implant (SINUVA®) prior to Screening Visit.

    d. Subject has a history of non-steroid immunosuppressants (i.e., calcineurin inhibitors, interleukin inhibitors, selective immunosuppressants, TNF-α inhibitors, other immunosuppressants) use within the 60 days prior to Screening Visit.

    e. Subject has a history of new or recently modified allergen immunotherapy within the 3 months prior to Screening Visit.

    f. Subject has a history of monoclonal antibody therapy (e.g., dupilumab, mepolizumab, omalizumab, benralizumab, reslizumab) within the 3 months prior to Screening Visit.

    g. Subject has a history of new or recently modified leukotriene modifiers therapy within the 1 month prior to Screening Visit.

    h. Subject has a history of new or recently modified β-adrenoceptor agonists therapy within the 1 month prior to Screening Visit.

090177e19e292258\Approved\Approved On: 28-Jul-2023 15:57 (GMT)

   i. Subject has had asthma exacerbation requiring systemic corticosteroid treatment, emergency department visit, or hospitalization within the 3 months prior to Screening Visit.

   j. Use of any prohibited concomitant medication(s) as specified in Section 5.4.

7. Other Exclusion Criteria

   a. Prisoners or subjects who are involuntary incarcerated.

   b. Subjects who are compulsorily detained for treatment of either a psychiatric or physical (e.g., infectious disease) illness.

   c. Subject participation in clinical trial with non-biological investigational agents or investigational interventional treatments within the 30 days prior to Screening Visit.

   d. Subjects who have previously participated in any BHV-3000/ BMS-927711/ rimegepant study.

   e. Subject participation in clinical trial with biological investigational agents within the 3 months prior to the Screening Visit.

   f. Subjects who meet criteria for C-SSRS Suicidal Ideation Items 4 or 5 within the last 12 months prior to screening, OR subjects who endorse any of the five C-SSRS Suicidal Behavior Items (actual attempt, interrupted attempt, aborted attempt, preparatory acts, or behavior) within the last 10 years prior to screening, OR subjects who, in the opinion of the Investigator, present a serious risk of suicide (See Section 6.2.5).

   g. Planned participation in any other investigational clinical trial while participating in this clinical trial.

   h. Subjects engaged in, or with plans to engage in, litigation or Worker's Compensation in which monetary gain or loss (or other compensation) may affect their objective participation in the trial.

   i. Investigator site staff directly involved in the conduct of the study and their family members, site staff otherwise supervised by the investigator, and sponsor and sponsor delegate employees directly involved in the conduct of the study and their family members.

   j. Subject unable to complete eDiary independently in the opinion of the Investigator.

Please see Section 5.4 for Prohibited and Restricted Concomitant Medications and Section 5.5 for Daily and Rescue Medications.

090177e19e292258\Approved\Approved On: 28-Jul-2023 15:57 (GMT)

5.4 Prohibited and Restricted Concomitant Medications

The below medications are prohibited prior to randomization and during the course of this study or as specified.

1. History of non-narcotic analgesic intake on ≥ 15 days per month for greater ≥ 3 month (e.g., acetaminophen, NSAIDs, gabapentin etc.). (Please refer to Section 5.5 for rescue medications).

2. Use of all acetaminophen or acetaminophen containing products must be discontinued at least 2 days prior to randomization (acetaminophen ≤ 1000 mg/day is allowed as rescue medication, see Section 5.5). During the screening phase (3 to 14 days) and throughout the study, the use of acetaminophen or acetaminophen containing products at daily dosing levels of greater than 1000 mg/day is prohibited.

3. Systemic corticosteroids (e.g., prednisone, prednisolone, methylprednisolone, betamethasone, dexamethasone, triamcinolone, hydrocortisone, cortisone) should not be taken 30 days prior to the Screening Visit and throughout the study. Subjects should have access to systemic corticosteroids during the trial for treatment of symptoms, if indicated. However, subjects should discontinue the study if they receive this therapy.

4. Antibiotics, other than topical nasal antibiotics, should not be taken 14 days prior to the Screening Visit and throughout the study. Subjects should have access to antibiotics during the trial for treatment of symptoms, if indicated. However, subjects should discontinue the study if they receive this therapy.

5. Monoclonal antibody therapy (e.g., dupilumab, mepolizumab, omalizumab, benralizumab, reslizumab) should not be taken 3 months prior to the Screening Visit and throughout the study.

6. St. John's Wort should not be taken 14 days prior to the Baseline Visit and throughout the study.

7. Barbiturate-containing products (e.g., Fioricet, Fiorinal, butalbital, phenobarbital) should not be taken 14 days prior to the Baseline Visit and throughout the study.

8. Modafinil (PROVIGIL®) should not be taken 14 days prior to the Baseline Visit and throughout the study.

9. The use of CGRP antagonists (biologic [e.g., Aimovig® (erenumab-aooe), Ajovy® (fremanezumab-vfrm), Emgality® (galcanezumab-gnlm), Vyepti® (eptinezumab-jjmr)] or small molecule) other than rimegepant is prohibited during the study. CGRP antagonist biologics must be discontinued 3 months prior to the Baseline Visit. CGRP small molecule antagonists must be discontinued 14 days prior to the Baseline Visit and throughout the study.

090177e19e292258\Approved\Approved On: 28-Jul-2023 15:57 (GMT)

10. Use of narcotic medication, such as opioids (e.g., morphine, codeine, oxycodone and hydrocodone) should not be taken 2 days prior to the Baseline Visit and throughout the study.

11. Use of cannabinoids (e.g., marijuana, CBD) is prohibited from the Baseline Visit and throughout the study.

12. Concomitant use of strong CYP3A4 inhibitors, such as HIV Protease Inhibitors, Hepatitis C protease inhibitors, certain azole antifungals, or clarithromycin, is prohibited during the study. Strong CYP3A4 inhibitors must be discontinued 14 days prior to the Baseline Visit and throughout the course of the study. Please see Section 16.1 for additional resources.

13. Concomitant use of moderate to strong CYP3A4 inducers, such as carbamazepine, phenytoin, or rifampin, is prohibited during the study. Moderate or strong CYP3A4 inducers must be discontinued 14 days prior to the Baseline Visit and throughout the course of the study. Please see Section 16.1 for additional resources.

14. Atypical antipsychotics such as Abilify (aripiprazole), Zyprexa (olanzapine), Seroquel (quetiapine), Geodon (ziprasidone), or Risperdal (risperidone) or Depakote/Depakene (valproic acid/valproate) should not be taken 90 days prior to the Baseline Visit and throughout the study.

15. LAMICTAL (lamotrigine) should not be taken 90 days prior to the Baseline Visit and throughout the study.

16. Topical nasal decongestants should not be taken more than 3 times per week.

Subjects with daily use of medications listed in Section 5.5 are permitted to remain on therapy provided they have been on a stable dose for at least 1 month prior to the Screening Visit.

Low dose aspirin (e.g., up to 100 mg) for documented cardiovascular prophylaxis is allowed.

5.5 Daily and Rescue Medications

Medication doses must be **stable for at least one month prior to the Screening Visit** and throughout the study. Subjects may use:

- Saline and other non-medicated spray/rinse (additives such as detergents, moisturizers, mucolytics are permitted) – maintain dosing regimen established prior to the Screening Visit

AND **up to two of the topical nasal medications (stable dosing, not PRN, any indication)**:

- Topical nasal corticosteroids (spray or rinse)
- Topical nasal antibiotics and antifungal medications (spray or rinse)
- Topical nasal antihistamines (non-sedating)

090177e19e292258\Approved\Approved On: 28-Jul-2023 15:57 (GMT)

- Topical nasal cromolyn

AND any of the following medications (**stable dosing, not PRN**):

- Leukotriene modifiers
- Cromolyn (oral or inhalation)
- Methylxantines (e.g., theophylline, aminophyllines)
- Mucoactive agents (e.g., guaifenesin, acetylcysteine)

Subjects with concomitant asthma or COPD may remain on their baseline SoC treatment if they have been on a stable dose for at least 1 month prior to the Screening Visit and if not specifically listed in Section 5.4 Prohibited and Restricted Concomitant Medications, and the dose is not expected to change during the course of the study. Subjects may use their rescue inhalers and/or nebulizers as directed by their healthcare provider (HCP).

After dosing with study medication, all other CRS medication is prohibited during the 2 hours post-dose. At the end of 2 hours after dosing with study medication (and after the 2-hour assessments have been completed on the eDiary), subjects will be permitted to use the following rescue medication: acetaminophen (up to 1000 mg/day), or aspirin, ibuprofen, naproxen (or any other type of non-steroidal anti-inflammatory drug (NSAID)), oral antihistamines (non-sedating), oral decongestants, topical nasal decongestants, topical nasal anticholinergics (all up to the daily recommended dose indicated on the drug packaging). These are the only medications allowed for rescue treatment after 2 hours post dose of study medication.

However, if needed, after 24 hours of administering the one dose of study medication (and before coming in for the End of Treatment Visit) subject may take their prescribed standard of care medications for treatment of CRS symptoms provided all the assessments have been completed on the eDiary. Exclusionary rescue medication such as opioids, butalbital compounds, are not allowed on this study. Similarly, if the facial pain/pressure/fullness is relieved by study medication at 2 hours after dosing but then recurs to intensity level of ≥ 6 on NRS (0-10) between 2 and 24 hours, the subject will be permitted to take the same rescue therapy as outlined above. In all circumstances, the subject will always continue to complete his or her eDiary entries through the 24-hour assessment after consuming the study medication.

Use of concomitant medication after randomization, including rescue medication, will be recorded by the subject on a paper diary and reported to the site. The site will record all medications that were taken from 14 days before the Screening Visit through the end of the End of Treatment Visit.

During the 45-day Treatment Phase, if the subject has a **non-qualifying** facial pain/pressure/fullness (i.e., facial pain/pressure/fullness < 6 on a NRS (0-10)), the subject is permitted to use only the following medications: acetaminophen (up to 1000 mg/day), or aspirin, ibuprofen, naproxen (or any other type of nonsteroidal anti-inflammatory drug [NSAID]), oral

090177e19e292258\Approved\Approved On: 28-Jul-2023 15:57 (GMT)

antihistamines (non-sedating), oral decongestants, topical nasal decongestants, topical nasal anticholinergics (all up to the daily recommended dose indicated on the drug packaging).

Subjects should have access to systemic corticosteroids and antibiotics during the trial for treatment of symptoms, if indicated. However, subjects should discontinue the study if they receive those therapies.

5.6 Contraception

The investigator or their designee, in consultation with the subject, will confirm that the subject is utilizing an appropriate method of contraception for the individual subject from the permitted list of contraception methods (see Section 16.3.4) and will confirm that the subject has been instructed in its consistent and correct use.

The investigator or designee will inform the subject of the need to use acceptable effective contraception consistently and correctly and document the conversation and the subject's affirmation in the subject's chart. Subjects need to affirm their consistent and correct use of at least 1 of the selected methods of contraception, considering that their risk for pregnancy may have changed since the last visit.

In addition, the investigator or designee will instruct the subject to call immediately if the selected contraception method is discontinued and document the requirement to use an alternate protocol-specified method, including if the subject will no longer use abstinence as the selected contraception method, or if pregnancy is known or suspected in the subject or partner.

5.7 Other Restrictions and Precautions

Not applicable.

5.8 Protocol Deviations and Quality Tolerance Limits

Any significant event that does not comply with the inclusion/exclusion criteria, study conduct, or study procedures will be documented as a deviation. Deviations will be documented and reported through the clinical monitoring of the trial. Deviations will be reported to the IRB/EC at the frequency required by your IRB/EC. Prospective approval of protocol deviations to recruitment and enrollment criteria, also known as protocol waivers or exemptions, is not permitted. See Section 10.1 for more information on protocol deviations.

In addition, Quality Tolerance Limits (QTLs) are predefined parameters that are monitored during the study. Important deviations from the QTLs and any remedial actions taken will be summarized in the CSR.

090177e19e292258\Approved\Approved On: 28-Jul-2023 15:57 (GMT)

090177e19e292258\Approved\Approved On: 28-Jul-2023 15:57 (GMT)

6 STUDY CONDUCT AND DESCRIPTION OF STUDY PROCEDURES

6.1 Study Materials

The following study materials may be provided:

- Investigator File/Regulatory Binder
- Pharmacy Binder
- Investigational Product Manual
- Drug Accountability Logs
- Sample source documents, where applicable
- Paper diary to record Concomitant and Rescue Medication (take home for subject)
- Investigator's Brochure
- Interactive Web-based Response System (IWRS) Manual
- Electronic Diary (eDiary): 1 will be given to each randomized subject
- Instructions for the eDiary device and access to the portal
- Sino-Nasal Outcome Test (SNOT-22)
- Laboratory Kits and Laboratory Manual
- Home Pregnancy Test
- ECG Machine and Instructions
- Columbia-Suicide Severity Rating Scale
- Serious Adverse Event (SAE) forms (to be used only if PSSA is not available)
- Exposure during Pregnancy Supplemental Forms
- Pregnant Partner Release of Information Form

All sites will use an Electronic Data Capture (EDC) tool to submit study data to the sponsor's CRO. Electronic Patient Reported Outcomes (ePRO) will be used for all patient-rated scales and will be captured on an eDiary. Any assessment completed by the subject in the eDiary will be transferred from the site/subject to the vendor and from the vendor to the CRO and/or sponsor.

No additional source documents are required for scales and assessments completed by the subject in eDiary.

Safety laboratory, plasma, serum, instructions for all specimens collected will be provided by a designated central laboratory. ECG equipment, supplies, instructions, and training materials will be supplied by a centralized ECG vendor.

6.2 Safety Assessments

6.2.1 Vital Signs and Physical Measurements (Height and Weight)

Sitting arterial systolic and diastolic blood pressure and pulse rate, height, weight and oral body temperature will be measured at Screening and End of Treatment. Height and weight will be measured at Screening only see Table 1.

6.2.2 Electrocardiogram (ECG)

A standard 12-lead ECG will be recorded during the Screening Phase and at the scheduled visits as outlined in Table 1. ECG data will be submitted to a central ECG vendor for measurement and interpretation. The final ECG report from the central vendor should be maintained in the subject's source documentation and be used for the final interpretation of the ECG recording.

Based on the central ECG vendor report, the Investigator will determine if subject is eligible and if any abnormalities are clinically significant or not. Any clinically significant changes from the Screening ECG may potentially be AEs (Section 16.2) and should be evaluated further, as clinically warranted.

6.2.3 Physical Exam

Subjects will undergo a routine physical examination during the Screening Phase and at the scheduled visits as outlined in Table 1. Physical examinations include examination of the heart, abdomen, lungs, and any other body system to be guided by symptoms.

6.2.4 Laboratory Assessments

All protocol-required laboratory assessments must be conducted in accordance with the laboratory manual and the Schedule of Assessments (Table 1). Unscheduled clinical laboratory measurements may be obtained at any time during the study to assess any perceived safety issues.

The investigator must review the laboratory report, document this review, and record any clinically relevant changes occurring during the study in the AE section of the CRF. The laboratory reports must be filed with the source documents. Clinically significant abnormal laboratory findings are those which are not associated with the underlying disease, unless judged by the investigator to be more severe than expected for the subject's condition.

090177e19e292258\Approved\Approved On: 28-Jul-2023 15:57 (GMT)

6.2.4.1 Safety Laboratory Testing

Blood and urine samples will be obtained as outlined in Table 1 for clinical laboratory evaluations. A central laboratory vendor will be utilized for this study and a laboratory manual will be provided to each site. **If possible, subjects should be fasting for a minimum of 8 hours prior to all blood draws.** However, if a subject is not fasting at a given visit, the blood draw should still be performed, and the non-fasting status should be documented.

Hematology: Hemoglobin, hematocrit, red blood cell (RBC) count, white blood cell (WBC) count with differential, and platelets.

Blood chemistry/electrolyte: Sodium, potassium, chloride, bicarbonate, calcium; glucose, BUN (urea), serum creatinine, uric acid, ALT, AST, alkaline phosphatase, LDH, total protein, albumin, total bilirubin, direct bilirubin, indirect bilirubin, CK. End of Treatment Visit – elevations in CK (>5x ULN) may have further CK fractionation tests performed through the central lab.

Estimated glomerular filtration rate: eGFR using the estimated MDRD formula will be calculated and reported by the central lab at each visit that clinical laboratory tests are collected as outlined in Table 1.

Urinalysis: pH, specific gravity, ketones, nitrites, urobilinogen, leukocyte esterase, protein, glucose and blood. If blood, protein or leukocytes are positive, reflex to microscopic examination.

Urine Drug Screen: For drugs of abuse.

FSH: FSH levels will be measured at the Screening Visit on post-menopausal women to confirm post-menopausal status. FSH level testing is not required for women greater than or equal to 62 years old with amenorrhea of greater than or equal to 1 year.

HbA1c at screening.

Lipid Panel (triglycerides, total cholesterol, HDL, and LDL) will be measured at the Screening Visit.

Reflex tests:
If ALT or AST ≥ 3 x ULN OR total bilirubin ≥ 2 x ULN at any visit after the baseline visit, the central laboratory will perform reflex tests that may include: CK, GGT, and anti-viral serologies. Subjects may have to return to the study site to provide additional blood samples for these laboratory tests.

Additional laboratory tests may be obtained to evaluate laboratory abnormalities and/or adverse events.

090177e19e292258\Approved\Approved On: 28-Jul-2023 15:57 (GMT)

6.2.4.2 Pregnancy Testing

Pregnancy tests will be conducted (serum, urine, or home pregnancy test), if appropriate prior to randomization, and as outlined in Table 1.

6.2.5 Columbia-Suicide Severity Rating Scale (C-SSRS)

The Columbia-Suicide Severity Rating Scale (C-SSRS) is a questionnaire used for suicide Assessment.[25] The C-SSRS "Screening version" will be used at the Screening Visit and the "Since Last Visit version"[26] will be used at subsequent visits in this study.

The C-SSRS Assessment is intended to help establish a person's immediate risk of suicide. The C-SSRS is a clinician administered scale that should be administered by a certified rater. This scale will be collected on site with a paper form. The C-SSRS should be reviewed by the Investigator or designee before the subject is allowed to leave clinic.

At the Screening Visit, the recall period for completing is 12 months for suicidal ideation and 10 years for suicidal behavior; at all other visits, the recall period for completing the C-SSRS is since the last visit (Table 1).

Any "Yes" responses must be immediately evaluated by the investigator. If the Investigator determines that a subject is at risk of suicide, self-harm, appropriate measures to ensure the subject's safety and obtain mental health evaluation must be implemented. In such circumstances, the subject must immediately be discontinued from the study. The event should be recorded as either an AE or SAE as determined by the Investigator and reported within 24 hours to the sponsor.

6.2.6 Weekly Contact

Subjects should be contacted (e.g., via phone, text, email) at least weekly by study staff to monitor (and remind them to report) any systemic corticosteroid and/or antibiotics (other than topical nasal antibiotics) use. If a subject reports taking one of these therapies before dosing, study staff should withdraw the subject from the study, inform the subject that they should not dose with study medication, and schedule an End of Treatment visit.

6.3 Efficacy Assessments

6.3.1 Facial pain/pressure/fullness

Subjects will record in their eDiary their current facial pain/pressure/fullness score, on a NRS (0-10) at the time points indicated in Table 1.

6.3.2 Nasal obstruction (congestion)

Subjects will record in their eDiary their current nasal obstruction (congestion) score, on a NRS (0-10) at the time points indicated in Table 1.

090177e19e292258\Approved\Approved On: 28-Jul-2023 15:57 (GMT)

6.3.3 Nasal discharge

Subjects will record in their eDiary their current nasal discharge (anterior, posterior, or both) score, on a NRS (0-10) at the time points indicated in Table 1.

6.3.4 Headache pain

Subjects who report moderate or severe headache pain at the time of qualifying facial pain/pressure/fullness will record in their eDiary their current headache pain score on a 4-point Likert scale (0 = None; 1 = Mild; 2 = Moderate; 3 = Severe) at the time points indicated in Table 1.

6.3.5 Rescue Medication

The subject's use of rescue medication is recorded by the subject on a paper diary and reviewed by site staff.

6.3.6 Patient Global Impression of Change (PGI-C) Questionnaire

The PGI-C is a patient-rated scale which assesses how the subject's current illness state has changed relative to the baseline visit. The subject is asked to rate a change in their overall disease condition on a 7-point Likert scale, with the following response options: 1 = very much improved, 2 = much improved, 3 = minimally improved, 4 = no change, 5 = minimally worse, 6 = much worse, and 7 = very much worse. The PGI-C is a global index scale that may be used to rate the response of a condition to a therapy. The eDiary is used to evaluate the PGI-C Questionnaire at the time points indicated in Table 1.

6.3.7 Sino-Nasal Outcome Test (SNOT-22)

SNOT-22 is a validated symptom-based outcome measure to assess the burden of CRS symptomatology. It is a 22-item instrument that includes both nasal and extra-nasal symptoms such as poor sleep and mood disturbance. The SNOT-22 will be collected on paper at the Baseline Visit and on the eDiary at 24 hours post-dose (see Table 1).

6.4 Early Discontinuation from the Study

Subjects MUST discontinue investigational product (and non-investigational product at the discretion of the Investigator) for any of the following reasons:

- Withdrawal of informed consent (subject's decision to withdraw for any reason).
- Any clinical adverse event (AE), laboratory abnormality or intercurrent illness which, in the opinion of the Investigator or sponsor, indicates that continued participation in the study is not in the best interest of the subject.
- Pregnancy

090177e19e292258\Approved\Approved On: 28-Jul-2023 15:57 (GMT)

- Termination of the study by Pfizer Inc.
- Loss of ability to freely provide consent through imprisonment or involuntary incarceration for treatment of either a psychiatric or physical (e.g., infectious disease) illness.
- Use of systemic corticosteroids.
- Use of antibiotics (other than topical nasal antibiotics).

All subjects who discontinue should comply with protocol specified End of Treatment procedures as outlined in Table 1. The only exception to this requirement is when a subject withdraws consent for all study procedures or loses the ability to consent freely (i.e., is incapacitated, imprisoned, or involuntarily incarcerated for the treatment of either a psychiatric or physical illness).

6.5 Lost to Follow Up

A subject will be considered lost to follow-up if he or she repeatedly fails to return for scheduled visits and is unable to be contacted by the study site.

The following actions must be taken and documented if a subject fails to return to the clinic for a required study visit:

The site must attempt to contact the subject and reschedule the missed visit as soon as possible and counsel the subject on the importance of maintaining the assigned visit schedule and ascertain whether or not the subject wishes to and/or should continue in the study.

Before a subject is deemed lost to follow up, the Investigator or designee must make every effort to regain contact with the subject (where possible, 3 telephone calls and, if necessary, a certified letter to the subject's last known mailing address or local equivalent methods). These contact attempts should be documented in the subject's medical record.

Should the subject continue to be unreachable, he/she will be considered to have withdrawn from the study.

090177e19e292258\Approved\Approved On: 28-Jul-2023 15:57 (GMT)

7 STUDY DRUG MANAGEMENT

7.1 Description of Study Drug

7.1.1 Investigational Product

An investigational product, also known as investigational medicinal product in some regions, is defined as follows:

A pharmaceutical form of an active substance or placebo being tested or used as a reference in a clinical study, including products already with a marketing authorization but used or assembled (formulated or packaged) in a way different from the authorized form, or used for an unauthorized indication, or when used to gain further information about the authorized form.

The investigational product should be stored in a secure area according to local regulations. It is the responsibility of the Investigator to ensure that investigational product is only dispensed to study subjects. The investigational product must be dispensed only from official study sites by authorized personnel according to local regulations.

In this protocol, investigational product is/are:

Rimegepant ODT and matching placebo. The rimegepant ODT and the matching placebo appear identical visually, via touch, smell and taste.

7.1.2 Packaging, Shipment and Storage

The product storage manager should ensure that the study drug is stored in accordance with the environmental conditions (temperature and light) as determined by the sponsor. Please see the Investigational Product Manual/Investigator's Brochure for specific conditions. If concerns regarding the quality or appearance of the study drug arise, do not dispense the study drug and contact the sponsor/CRO immediately.

7.2 Dose and Administration

One dose of rimegepant ODT (75 mg) or matching placebo will be dispensed to the subject and will be taken at the time the subject has a facial pain/pressure/fullness that reaches intensity of ≥ 6 on the NRS (0-10) ***and after*** completing the eDiary questions.

7.2.1 Method of Assigning Subject Identification

At the time of enrollment, immediately after written informed consent is obtained and before performing any study-related procedures, each subject will be assigned, through an interactive web response system (IWRS), a unique subject number for identification throughout the study. This subject number must not be reused for any other subject in the study. The physician/coordinator must contact the IWRS to enroll each subject into a centralized database at the time of signing consent.

090177e19e292258\Approved\Approved On: 28-Jul-2023 15:57 (GMT)

After completion of all screening evaluations all eligible subjects will be randomized in a 1:1 ratio to the rimegepant or matching placebo treatment groups. The randomization will be stratified by the presence of nasal polyps (yes or no). It is important to correctly enter subjects who have nasal polyps in the IWRS system. Once a subject is stratified in the IWRS, this cannot be changed and will be considered a deviation.

Randomization schedules will be generated and kept by the IWRS vendor in a secure network folder with access limited to only unblinded team members. Each subject who is qualified for treatment will be randomized via the IWRS randomization option. Subjects will maintain their subject number assigned at screening throughout the trial. The IWRS will provide the double-blind treatment assignments.

The randomization will trigger dispensation for the subject and assign a bottle number for the appropriate randomized treatment type. The drug is dispensed at the time of randomization.

7.2.2 Selection and Timing of Dose and Administration

Study medication (one 75 mg ODT or matching placebo) will be packaged in a single blister unit in a subject-specific bottle. There are no dose adjustments in this study and subjects will receive one dose to treat one facial pain/pressure/fullness that reaches intensity of ≥ 6 on the NRS (0-10) within 45 days of randomization (Baseline Visit). Subjects will be dispensed the study medication at randomization (Baseline Visit) and will take the ODT from the blister/bottle at the time of onset of facial pain/pressure/fullness ≥ 6 on the NRS (0-10) **ONLY** after answering questions regarding their CRS symptoms in the eDiary device. The ODT should be placed on or under the tongue until fully dissolved then swallowed. Subjects should be instructed to use dry hands when handling the study medication.

7.2.3 Dose Modifications

There will be no dose adjustments in this study.

7.3 Blinding and Unblinding

Blinding is critical to the integrity of this clinical study. However, in the event of a medical emergency or pregnancy in an individual subject, in which knowledge of the investigational product is critical to the subject's management, the blind for that subject may be broken by the treating physician.

Before breaking the blind of an individual subject's treatment, the Investigator should have determined that the information is necessary, (i.e., that it will alter the subject's immediate management). In many cases, particularly when the emergency is clearly not investigational product related, the problem may be properly managed by assuming that the subject is receiving active product without the need for unblinding.

In cases of accidental unblinding, contact the Pfizer Medical Monitor and ensure every attempt to preserve the blind is made.

090177e19e292258\Approved\Approved On: 28-Jul-2023 15:57 (GMT)

7.4 Treatment Compliance

Responsible study personnel will dispense the study drug. Accountability and compliance verification should be documented in the subject's study records.

Subjects have to be counseled on the importance of taking the study drug as directed when a facial pain/pressure/fullness occurs and reaches intensity of ≥ 6 on the NRS (0-10). If the subject does not have a facial pain/pressure/fullness ≥ 6 on the NRS (0-10) or take their study medication within 45 days of the Baseline Visit, they should return to the clinic for their End of Study Visit and return their study medication.

7.5 Destruction and Return of Study Drug

All unused study drug can be sent back to the drug depot for destruction only after being inspected and reconciled by the responsible Study monitor or the sponsor's designee. If it is site policy to destroy study drug on site, it is the Investigator's responsibility to ensure that arrangements have been made for the disposal, procedures for proper disposal have been established according to the applicable regulations, guidelines and institutional procedures, and appropriate records of the disposal have been documented. The unused study drugs can only be destroyed after being inspected and reconciled by the responsible Study Monitor or the sponsor's designee.

090177e19e292258\Approved\Approved On: 28-Jul-2023 15:57 (GMT)

8 ADVERSE EVENTS

An Adverse Event (AE) is defined as any new untoward medical occurrence or worsening of a pre-existing medical condition in a subject or clinical investigation subject administered an investigational (medicinal) product and that does not necessarily have a causal relationship with this treatment. An AE can therefore be any unfavorable and unintended sign (including an abnormal laboratory finding for example) symptom, or disease temporally associated with the use of the investigational product, whether or not considered relate to the investigational product.

Adverse events can be spontaneously reported or elicited during an open-ended questioning, examination, or evaluation of a subject. In order to prevent reporting bias, subjects should not be questioned regarding the specific occurrence of one or more AEs.

If a specific diagnosis or syndrome is identified by the Investigator, this should be recorded as the AE, rather than recording (as separate AEs) the individual signs/symptoms or clinically significant laboratory abnormalities known to be associated with, and considered by the Investigator to be a component of, the disease/syndrome.

Definition of terms related to all Adverse Events (serious and non-serious):

Mild: Is usually transient and may require only minimal treatment or therapeutic intervention. The event does not generally interfere with usual activities of daily living.

Moderate: Is usually alleviated with additional specific therapeutic intervention. The event interferes with usual activities of daily living, causing discomfort but poses no significant or permanent risk of harm to the subject.

Severe: Interrupts usual activities of daily living, significantly affects clinical status, or may require intensive therapeutic intervention.

Life threatening AEs that are considered life threatening should be considered SAEs. An AE is life threatening if the subject was at immediate risk of death from the event as it occurred, i.e., it does not include a reaction that if it had occurred in a more serious form might have caused death. For example, drug induced hepatitis that resolved without evidence of hepatic failure would not be considered life threatening even though drug induced hepatitis can be fatal.

Hospitalization: AEs requiring hospitalization should be considered SAEs. Hospitalization for elective surgery or routine clinical procedures that are not the result of AE (e.g., elective surgery for a pre-existing condition that has not worsened) need not be considered AEs or SAEs. If anything untoward is reported during the procedure, that occurrence must be reported as an AE, either 'serious' or 'non-serious' according to the usual criteria.

090177e19e292258\Approved\Approved On: 28-Jul-2023 15:57 (GMT)

090177e19e292258\Approved\Approved On: 28-Jul-2023 15:57 (GMT)

Assessment for Determining Relationship of AE to Study Drug:

The relatedness of each AE to study drug must be classified based on medical judgement and according to the following categories. The definitions are as follows:

Related: This category applies to AEs that are considered, with a high degree of certainty, to be related to the study drug. An AE may be considered related when it follows a temporal sequence from the administration of study drug, it cannot reasonably be explained by the known characteristics of the subject's clinical state, environment, or toxic factors, or other modes of therapy administered to the subject. An AE may be considered related when it follows a known pattern of response to the study drug, or if the AE reappears upon re-challenge. This category also applies to AEs that are considered to have an unlikely connection to study drug, but a relationship cannot be ruled out with certainty.

Unrelated: This category applies to AEs that are considered with a high degree of certainty to be due only to extraneous causes (e.g., subject's clinical state, environment, toxic factors, disease under study, etc.) and does not meet the criteria of other categories above.

There are two types of adverse events, Serious Adverse Events (SAE) and Non-Serious Adverse Events (AEs).

8.1 Serious Adverse Events

8.1.1 Definition of Serious Adverse Event (SAE)

An SAE is any event that meets any of the following criteria at any dose:

- Death
- Life-threatening
- Inpatient hospitalization or prolongation of existing hospitalization
- Persistent or significant disability/incapacity
- Congenital anomaly/birth defect in the offspring of a subject who received rimegepant
- Other: Important medical events that may not result in death, be life-threatening, or require hospitalization, may be considered an SAE when, based upon appropriate medical judgment, they may jeopardize the subject and may require medical or surgical intervention to prevent one of the outcomes listed in this definition. Examples of such events are (but not limited to):
  - Intensive treatment in an emergency room or at home for allergic bronchospasm
  - Blood dyscrasias or convulsions that do not result in inpatient hospitalization

- Development of drug dependency or drug abuse
- Potential drug induced liver injury (see Section 8.5)
- Abuse or Overdose of medication
    - Potential study medication abuse (including cases of excessive non-compliance with study medication dosing instructions or subjects who discontinue treatment without returning study medication) should be documented in the source record and reported as an AE or SAE as appropriate. Investigators must monitor subjects for possible cases of abuse of study medication (subjects taking study drug for non-therapeutic purposes, e.g., for psychoactive effects such as high or euphoria). Investigators should obtain more information and explanation from subjects when there are study drug accountability discrepancies
    - Potential study medication overdose is defined in Section 8.3

In general, hospitalization signifies that the subject has been detained (usually involving at least an overnight stay) at the hospital or emergency ward for observation and/or treatment that would not have been appropriate in the physician's office or outpatient setting. When in doubt as to whether 'hospitalization' occurred or was necessary, the AE should be considered serious.

The following hospitalizations are not considered SAEs in Pfizer clinical studies (but may be considered non-serious AEs):

- A visit to the emergency room or other hospital department <24 hours that does not result in an admission (unless considered "important medical event" or event that is life threatening).
- Elective surgery planned prior to signing consent.
- Admissions as per protocol for a planned medical/surgical procedure.
- Routine health assessment requiring admission (i.e., routine colonoscopy).
- Admission encountered for another life circumstance that carries no bearing on health and requires no medical intervention (i.e., lack of housing, care-giver respite, family circumstances).

Disability/incapacitating: An AE is incapacitating or disabling if the experience results in a substantial and/or permanent disruption of the subject's ability to carry out normal life functions.

090177e19e292258\Approved\Approved On: 28-Jul-2023 15:57 (GMT)

090177e19e292258\Approved\Approved On: 28-Jul-2023 15:57 (GMT)

8.1.2 Collection and Reporting Serious Adverse Events

Following the subject's written consent to participate in the study, all SAEs, whether related or not related to study drug, must be collected, including those thought to be associated with protocol-specific procedures. All SAEs must be collected that occur from the time the subject provides informed consent, throughout the course of the study, and up to 30 days after last dose. The Investigator should report any SAE occurring after this time period that is believed to be related to study drug or protocol-specific procedures.

All SAEs should be followed to resolution or stabilization.

An SAE report should be completed for any event where doubt exists regarding its status of seriousness.

If the Investigator believes that an SAE is not related to the study drug, but is potentially related to the conditions of the study (such as a withdrawal of previous therapy or a complication related to study procedure), the relationship should be specified in the narrative section of the SAE Report.

SAEs, whether related or not related to study drug, overdose when associated with an SAE (see Section 8.3), potential drug induced liver injury (see Section 8.5) and pregnancies (see Section 8.4) must be reported within 24 hours of the Investigator becoming aware of the event. The Investigator is responsible for submitting all applicable events to the Independent Review Board (IRB) as per the IRB's reporting requirements. Additionally, the Investigator, or designated staff, is responsible for entering the SAE information into the Case Report Form (CRF) and safety reporting system (i.e., event term, start/stop dates, causality, and severity).

Any serious adverse event must be reported immediately or no later than 24 hours after awareness of the event to Pfizer DSU either via the Pfizer SAE Submission Assistant (PSSA) tool or as a written description, using the Pfizer CT SAE report form, that must be sent by facsimile (fax or eFax) to the Pfizer DSU at 1-866-997-8322.

If only limited information is initially available, follow-up reports are required. If an ongoing SAE changes in its intensity or relationship to study drug or if new information becomes available, a follow-up SAE report should be sent within 24 hours of the Investigator becoming aware of the updated information using the same procedure used for the transmission of the initial SAE and the same event term should be used.

The minimum information required for an initial SAE report is:

- Sender of report (Site number, Investigator name)
- Subject identification (subject number)
- Protocol number

- SAE term (if an SAE is being reported)

8.2 Non-serious Adverse Events

A ***non-serious adverse event*** is an AE not classified as serious.

8.2.1 Collection and Reporting of Non-Serious Adverse Events

The collection of non-serious AE information should begin at the time the subject provides informed consent through the End of Treatment Visit.

Non-serious adverse events should be followed until conclusion or stabilization, or reported as SAEs if they become serious. Follow-up is also required for non-serious AEs that cause interruption or discontinuation of study drug or those that are present at the end of study treatment.

8.2.2 Laboratory Test Abnormalities

The following laboratory test abnormalities should be captured on the non-serious AE CRF page or SAE Report Form (paper or electronic) as appropriate:

- Any laboratory test result that is clinically significant or meets the definition of an SAE.
- Any laboratory abnormality that required the subject to have the study drug discontinued or interrupted.
- Any laboratory abnormality that required the subject to receive specific corrective therapy.

8.3 Overdose

An overdose is defined as the accidental or intentional administration of any dose of a product that is considered both **excessive** and **medically important**.

Overdose is reportable to Pfizer Safety only when associated with an SAE. Details of any signs or symptoms and their management should be recorded including details of any treatments administered.

Asymptomatic dosing errors (e.g., accidentally taking two tablets instead of prescribed dose of one tablet in one calendar day) should be reported as deviations.

8.4 Pregnancy

If, following the Baseline Visit, it is subsequently discovered that a study subject is pregnant or may have been pregnant at the time of the investigational product exposure, including during at least 6 half-lives after the product administration, the investigational product will be permanently discontinued in an appropriate manner (i.e., dose tapering if necessary for subject safety). Protocol-required procedures for study discontinuation and follow-up must be performed on the

090177e19e292258\Approved\Approved On: 28-Jul-2023 15:57 (GMT)

subject unless contraindicated by the pregnancy (i.e., x-ray studies). Other appropriate pregnancy follow-up procedures should be considered if indicated.

Sites should instruct patients to contact the Investigator if they become pregnant during the course of the study. The Investigator must immediately notify the Pfizer Medical Monitor (or designee) and report the event by either using the PSSA tool or by completing an Exposure During Pregnancy (EDP) Supplemental Form following the SAE reporting procedures as described in Section 8.1.2.

Follow-up information regarding the course of the pregnancy, including perinatal and neonatal outcome and, where applicable offspring information must also be reported on an EDP Supplemental Form.

Any pregnancy that occurs in a female partner of a male study subject should be reported to the Pfizer DSU. Information on this pregnancy will be collected on an EDP Supplemental Form, as appropriate. In the case of paternal exposure, the investigator will provide the subject with the Pregnant Partner Release of Information Form to deliver to his partner. The investigator must document in the source documents that the subject was given the Pregnant Partner Release of Information Form to provide to his partner.

8.5 Potential Drug Induced Liver Injury (DILI)

Humans exposed to a drug who show no sign of liver injury (as determined by elevations in transaminases) are termed "tolerators," while those who show transient liver injury but adapt are termed "adaptors." In some subjects, transaminase elevations are a harbinger of a more serious potential outcome. These subjects fail to adapt and therefore are "susceptible" to progressive and serious liver injury, commonly referred to as DILI. Subjects who experience a transaminase elevation above 3 × ULN should be monitored more frequently to determine if they are "adaptors" or are "susceptible."

In the majority of DILI cases, elevations in AST and/or ALT precede T bili elevations (>2 × ULN) by several days or weeks. The increase in T bili typically occurs while AST/ALT is/are still elevated above 3 × ULN (ie, AST/ALT and T bili values will be elevated within the same laboratory sample). In rare instances, by the time T bili elevations are detected, AST/ALT values might have decreased. This occurrence is still regarded as a potential DILI. Therefore, abnormal elevations in either AST OR ALT in addition to T bili that meet the criteria outlined below are considered potential DILI (assessed per Hy's law criteria) cases and should always be considered important medical events, even before all other possible causes of liver injury have been excluded.

090177e19e292258\Approved\Approved On: 28-Jul-2023 15:57 (GMT)

The threshold of laboratory abnormalities for a potential DILI case depends on the subject's individual baseline values and underlying conditions. Subjects who present with the following laboratory abnormalities should be evaluated further as potential DILI (Hy's law) cases to definitively determine the etiology of the abnormal laboratory values:

- Subjects with AST/ALT and T bili baseline values within the normal range who subsequently present with AST OR ALT values ≥3 × ULN AND a T bili value ≥2 × ULN with no evidence of hemolysis and an alkaline phosphatase value <2 × ULN or not available.

- For subjects with baseline AST **OR** ALT **OR** T bili values above the ULN, the following threshold values are used in the definition mentioned above, as needed, depending on which values are above the ULN at baseline:

    - Preexisting AST or ALT baseline values above the normal range: AST or ALT values ≥2 times the baseline values AND ≥3 × ULN; or ≥8 × ULN (whichever is smaller).

    - Preexisting values of T bili above the normal range: T bili level increased from baseline value by an amount of ≥1 × ULN or if the value reaches ≥3 × ULN (whichever is smaller).

Rises in AST/ALT and T bili separated by more than a few weeks should be assessed individually based on clinical judgment; any case where uncertainty remains as to whether it represents a potential Hy's law case should be reviewed with the sponsor. The subject should return to the investigator site and be evaluated as soon as possible, preferably within 48 hours from awareness of the abnormal results. This evaluation should include laboratory tests, detailed history, and physical assessment.

If any potential DILI is identified and meets the criteria above, the Pfizer Medical Monitor (or designee) should immediately be contacted for further instruction on whether the subject must discontinue from the trial and appropriate follow-up requirements.

All cases demonstrated on repeat testing as meeting the laboratory criteria of AST/ALT and T bili elevation defined above should be considered potential DILI (Hy's law) cases if no other reason for the LFT abnormalities has yet been found. **Such potential DILI (Hy's law) cases are to be reported as SAEs, irrespective of availability of all the results of the investigations performed to determine etiology of the LFT abnormalities.**

A potential DILI (Hy's law) case becomes a confirmed case only after all results of reasonable investigations have been received and have excluded an alternative etiology.

8.6 Adverse Events of Special Interest

None

090177e19e292258\Approved\Approved On: 28-Jul-2023 15:57 (GMT)

8.7 Environmental Exposure, Exposure During Breastfeeding, and Occupational Exposure

Environmental exposure occurs when a person not enrolled in the study as a subject receives unplanned direct contact with or exposure to the study intervention. Such exposure may or may not lead to the occurrence of an AE or SAE. Persons at risk for environmental exposure include healthcare providers, family members, and others who may be exposed. An environmental exposure may include EDP, exposure during breastfeeding (EDB), and occupational exposure.

Any such exposures to the study intervention under study are reportable to Pfizer Safety within 24 hours of investigator awareness.

8.7.1 Exposure During Breastfeeding

An EDB occurs if:

- A female subject is found to be breastfeeding while receiving or after discontinuing study intervention.
- A female nonsubject is found to be breastfeeding while being exposed or having been exposed to study intervention (i.e., environmental exposure). An example of environmental EDB is a female family member or healthcare provider who reports that she is breastfeeding after having been exposed to the study intervention by ingestion.

The investigator must report EDB to Pfizer Safety within 24 hours of the investigator's awareness, irrespective of whether an SAE has occurred. The information must be reported via PSSA tool or by completing the CT SAE Report Form. When EDB occurs in the setting of environmental exposure, the exposure information does not pertain to the subject enrolled in the study, so the information is not recorded on a CRF. However, a copy of the completed report is maintained in the investigator site file.

An EDB report is not created when a Pfizer drug specifically approved for use in breastfeeding women (e.g., vitamins) is administered in accordance with authorized use. However, if the infant experiences an SAE associated with such a drug, the SAE is reported together with the EDB.

8.7.2 Occupational Exposure

The investigator must report any instance of occupational exposure to Pfizer Safety within 24 hours of the investigator's awareness via PSSA tool or by completing the CT SAE Report Form regardless of whether there is an associated SAE. Since the information about the occupational exposure does not pertain to a subject enrolled in the study, the information is not recorded on a CRF; however, a copy of the completed report is maintained in the investigator site file.

090177e19e292258\Approved\Approved On: 28-Jul-2023 15:57 (GMT)

8.8 Lack of Efficacy

The investigator must report signs, symptoms, and/or clinical sequelae resulting from lack of efficacy. Lack of efficacy or failure of expected pharmacological action is reportable to Pfizer Safety **only if associated with an SAE**.

8.9 Medication Errors

Medication errors may result from the administration or consumption of the study intervention by the wrong subject, or at the wrong time, or at the wrong dosage strength.
Medication errors are recorded and reported as follows:

Recorded on the Medication Error Page of the CRF	Recorded on the Adverse Event Page of the CRF	Reported on CT SAE Report Form/ via PSSA to Pfizer Safety Within 24 Hours of Awareness
All (regardless of whether associated with an AE)	Any AE or SAE associated with the medication error	Only if associated with an SAE

Medication errors include:

- Medication errors involving subject exposure to the study intervention
- Potential medication errors or uses outside of what is foreseen in the protocol that do or do not involve the study subject.

Whether or not the medication error is accompanied by an AE, as determined by the investigator, such medication errors occurring to a study subject are recorded on the medication error page of the CRF, which is a specific version of the AE page and, if applicable, any associated serious and nonserious AE(s) are recorded on the AE page of the CRF.

In the event of a medication dosing error, the sponsor should be notified within 24 hours. Medication errors should be reported to Pfizer Safety within 24 hours via PSSA tool or by completing the CT SAE Report Form **only when associated with an SAE.**

090177e19e292258\Approved\Approved On: 28-Jul-2023 15:57 (GMT)

9 STATISTICS

Complete details on the statistical methods for this study may be found the Statistical Analysis Plan (SAP).

9.1 General Procedures

Categorical variables are tabulated with counts and percentages. Continuous variables are summarized with univariate statistics (e.g., n, mean, standard error, median, minimum and maximum).

For the calculation of descriptive statistics of observed data, subjects must have a baseline value to be evaluable for endpoints based on values and changes from baseline over time.

Tabulations of the following endpoints present the number of unique subjects with an event: protocol deviations; interruptions of study therapy; nonstudy medications; adverse events; and laboratory abnormalities. Thus, for these endpoints, multiple occurrences of the same event are counted only once per subject.

9.2 Sample Size

If 70% of the 286 randomized (143 per treatment arm) have a facial pain/pressure/fullness which reaches pain intensity of ≥ 6 on the NRS (0-10) in the allotted time period, and complete their eDiary in the 24 hours following, we expect roughly 200 total or 100 per treatment group in the modified intent to treat (mITT) population for analysis.

Assuming rimegepant provides a 2-point reduction in NRS pain, and a 1.35-point advantage over placebo on the primary endpoint, and a common standard deviation of 3.0, then the study will have roughly 88% power on the primary endpoint. The estimates for change from baseline in NRS and common standard deviation are consistent with a modest reduction in pain over placebo and a conservative standard deviation estimate, as these varied widely in previous studies examining CRS.[27]

9.3 Populations for Analysis and Estimands

The set of enrolled subjects consists of all subjects who signed the informed consent form and were assigned a subject identification number.

The set of randomized subjects consists of enrolled subjects who were assigned a randomized treatment group.

The set of treated subjects consists of enrolled subjects who take study therapy (rimegepant or placebo).

090177e19e292258\Approved\Approved On: 28-Jul-2023 15:57 (GMT)

The Modified Intent to Treat (mITT) set consists of randomized subjects that take study therapy, have a facial pain/pressure/fullness which reaches pain intensity of ≥ 6 on the NRS (0-10) prior to administration of treatment, and provide at least one post-baseline efficacy data point.

Estimands

The estimand is a systematic description of the treatment effect to be quantified in order to answer the trial's research objective. The estimand consists of the following 5 attributes: Treatment, Population, Variable, Population-Level Summary, and Handling of Intercurrent Events (ICEs). Estimand for primary objective is discussed below.

Primary Estimand:

- Treatment: Rimegepant formulated in a 75 mg ODT (placed on or under the tongue) or a matching placebo
- Population: Subjects as defined by inclusion/ exclusion criteria of the study.
- Variable: Change from baseline of facial pain/pressure/fullness on NRS (0-10) score at 2 hours post-dose.
- Population-Level Summary: Difference in means and 95% CI, in change from baseline of facial pain/pressure/fullness on NRS (0-10) score at 2 hours post-dose, between Rimegepant and placebo.
- Intercurrent Event:
    - Use of nonstudy rescue medications on or before 2 hours post-dose
      Hypothetical strategy: In the event a subject uses a nonstudy rescue medication on or before 2 hours post-dose, all data points after rescue medication was administered are set to missing. It is assumed that, if the intercurrent event had not occurred and subject had followed the pattern of other subjects who continued follow-up without nonstudy rescue medication, their efficacy would have been like the efficacy of subjects from the same treatment group who did not use rescue medication. This is like missing at random (MAR) strategy.
    - All other intercurrent events
      Treatment policy strategy: All available assessments on the subject are used regardless of other intercurrent events.

Similarly, estimands for other objectives will be discussed in detail in the statistical analysis plan.

9.4 Statistical Methods

9.4.1 Demographic and Baseline Characteristics

Tabulations of demographic and baseline characteristics are made for: subjects randomized but not treated; subjects randomized and treated; and overall. A separate set of tabulations are made for subjects enrolled but not randomized.

090177e19e292258\Approved\Approved On: 28-Jul-2023 15:57 (GMT)

9.4.2 Primary Endpoint(s)

Primary endpoint will be analyzed according to primary estimand. The change from baseline efficacy endpoint is analyzed on the mITT population using a linear model that includes the baseline NRS (0-10) value for facial pain/pressure/fullness as a covariate, and fixed effects for treatment group, stratification factor (presence of nasal polyps; yes or no), scheduled time point, and time point by-treatment group interaction. Time points included in the model are nominally at 15, 30, 45, 60, 90 and 120 minutes post-dose.

Repeated measures within subject will be modeled using the unstructured covariance structure for within subject error. In the case the model fails to converge, a Huynh-Feldt error structure will be utilized, followed by an AR(1) structure. Error degrees of freedom will be calculated using the Kenward-Rogers approximation if an unstructured covariance structure fits appropriately; otherwise, a sandwich estimator will be utilized to estimate the covariance structure, and the degrees of freedom will be calculated using the between-within method.

The difference estimate (rimegepant - placebo), standard error, 95% confidence interval, and p-value will be reported for 2 hours post-dose.

In the event a subject uses a nonstudy rescue medication on or before 2 hours post-dose, all data points after rescue medication was administered will be set to missing (RM = M: Rescue medication use = Missing). Likewise, if a subject fails to log their pain score after study drug is administered, through 2 hours post-dose, this data will be considered missing (NC = M: Non-completers = Missing).

Sensitivity Analysis: The analysis of the primary endpoint assumes a hypothetical strategy for intercurrent event of prohibited rescue medication use on or before 2 hours post-dose. It further assumes that had the subjects not used a rescue medication, their efficacy would have been similar to the efficacy of subjects from the same treatment group who did not. As a result, the primary analysis uses a missing at random (MAR) strategy. The purpose of these sensitivity analyses is to investigate departures from these assumptions.

The following sensitivity analyses are planned:

- Jump to reference
- Tipping Point

All sensitivity analyses are conducted on the mITT population. Details of these analyses will be discussed in statistical analysis plan.

9.4.3 Secondary Endpoint(s)

Estimands for secondary endpoints will be defined in SAP. Analysis of secondary endpoints would follow the respective estimand strategy.

If the primary endpoint test is significant, then the secondary endpoints are evaluated using a hierarchical gate-keeping procedure, with each test in the hierarchy conducted at p=0.05. These

090177e19e292258\Approved\Approved On: 28-Jul-2023 15:57 (GMT)

secondary endpoints will be tested in the order shown in the Study Objectives section of this protocol. Secondary endpoints will be analyzed on the mITT population.

The analysis to evaluate change from baseline in the TNSS will be done using a linear model that includes the baseline TNSS value as a covariate, and fixed effects for treatment group, scheduled time point, and time point by-treatment group interaction. Time points included in the model are nominally at 15, 30, 45, 60, 90 and 120 minutes post-dose. The difference estimate (rimegepant - placebo), standard error, 95% confidence interval, and p-value will be reported for 2 hours post-dose.

The analysis to evaluate change from baseline in the nasal obstruction (congestion) score on the NRS (0-10) will be done in the same manner as the first secondary endpoint, with baseline nasal obstruction (congestion) score from the NRS as a covariate instead of baseline TNSS.

The analysis to evaluate change from baseline in the nasal discharge score on the NRS (0-10) will be done in the same manner as the first secondary endpoint, with baseline nasal discharge score from the NRS as a covariate instead of baseline TNSS.

The number of subjects that experience headache pain relief at 2 hours post-dose will be analyzed after first imputing missing data at 2 hours to be failure (i.e., NC = F: Non-Completers = Failure). Additionally, subjects who use rescue medication on or before assessment of pain at 2 hours will also be assigned as failures (RM = F: Rescue medication use = Failure). The analysis will be done using Mantel-Haenszel risk estimation after imputation is done, with stratification by presence of nasal polyps (yes or no). Headache pain is defined using a 4-point scale of none, mild, moderate, and severe. Headache pain relief is defined as a headache pain level of none or mild at 2 hours post-dose, with a headache pain level of moderate or severe at baseline. Note that only subjects with a headache pain level of moderate or severe at baseline will be included in this analysis.

The number of subjects that use rescue medication at 24 post-dose will be analyzed after first imputing missing data at 24 hours to be failure (NC = F). The analysis will be done using Mantel-Haenszel risk estimation after the missing data are imputed as failures, with stratification by presence of nasal polyps (yes or no).

9.4.4 Adjustment for Multiplicity

Type 1 error is controlled through the use of hierarchical testing. The significance of the primary endpoint is evaluated at the two-sided alpha level of 0.05. If the primary endpoint is significant, then the following secondary endpoints will be tested hierarchically in the order specified in the secondary objectives section (Section 2.2), each at the 0.05 level.

Thus, a secondary endpoint will be tested only if the preceding secondary endpoint in the hierarchy is determined to be significant. Descriptive p-values will be provided for any non-significant secondary endpoints and comparative exploratory endpoints.

090177e19e292258\Approved\Approved On: 28-Jul-2023 15:57 (GMT)

No attempt will be made to adjust for multiplicity when testing the exploratory endpoints. Any exploratory endpoints subjected to significance testing are evaluated at an unadjusted two-sided alpha level of 0.05.

9.4.5 Missing Data

For the primary endpoint of change in NRS on sinus pain/pressure/fullness from baseline, subjects who fail to record their pain at 2 hours post-dose will be considered missing (NC = M); the MMRM analysis model will be used, which assumes missing data are missing at random (MAR).

This is the approach for handling missing data that will also be used for the first, second, and third secondary endpoints of change from baseline in TNSS, in NRS for nasal obstruction (congestion), and NRS for nasal discharge, respectively.

For the secondary endpoint of proportion of subjects with headache pain relief from baseline at 2 hours post-dose, missing data at 2 hours post-dose will be imputed as a failure (NC = F).

For the secondary endpoint of proportion of subjects who use rescue medication at 24 hours post-dose, missing data at 24 hours post-dose will be imputed as a failure (NC = F).

Additional methods for handling of missing data, including sensitivity analyses, will be described in detail in the SAP.

9.4.6 Rescue Medication

For the primary endpoint of change in NRS on sinus pain/pressure/fullness from baseline, the intercurrent event of use of nonstudy rescue medications on or before the time point of interest (2 hours post-dose) will be handled with a hypothetical strategy. Specifically, the assumption will be that had the subject not used a rescue medication, their efficacy would have been similar to the efficacy of subjects from the same treatment group who did not use rescue medication. These data points after rescue medication was administered will be set to missing (RM = M).

This is the approach that will also be used for handling rescue medication use on or before 2 hours post-dose for the first, second, and third secondary endpoints of change from baseline in TNSS, in NRS for nasal obstruction (congestion), and NRS for nasal discharge, respectively.

For the secondary endpoint of proportion of subjects with headache pain relief from baseline at 2 hours post-dose, subjects that take rescue medication on or before 2 hours post-dose will be considered as failures (RM = F).

Rescue medications are described in more detail in Section 5.5.

090177e19e292258\Approved\Approved On: 28-Jul-2023 15:57 (GMT)

9.4.7 Analysis of Safety

The Investigators determine the intensity of AEs and the relationship of AEs to study therapy. The Investigators' terms are coded and grouped by system organ class using the latest version of the Medical Dictionary for Regulatory Activities (MedDRA) available. AEs are presented by system organ class and preferred term, ordered by the overall frequency of events. If a subject had an adverse event with different intensities over time, then only the greatest intensity is reported.

AEs are tabulated in all treated subjects. SAEs occurring in subjects enrolled but not treated are listed. Deaths are listed for enrolled subjects without regard to onset.

The frequencies of the following safety events are summarized by treatment regimen, and overall, for treated subjects: SAEs; all AEs, AEs by intensity; and AEs by relatedness.

Clinically significant laboratory test abnormalities will be identified as Grade 3 to 4 laboratory test results graded according to numeric laboratory test criteria in Common Terminology Criteria for Adverse Events (CTCAE) Version 5.0 (2017), if available. Otherwise, if CTCAE grades are not available, then results will be graded according to numeric laboratory test criteria in Division of AIDS (DAIDS) Table for Grading the Severity of Adult and Pediatric Adverse Events Corrected Version 2.1 (2017). If a subject has a laboratory test abnormality with different toxicity grades over time, then only the highest toxicity grade will be reported.

Further safety analyses will be described in the statistical analysis plan.

9.5 Schedule of Analyses

The data from this study may be locked and analyzed at any point after the last subject completes their end of treatment visit and adequate time has been allowed for follow-up.

090177e19e292258\Approved\Approved On: 28-Jul-2023 15:57 (GMT)

090177e19e292258\Approved\Approved On: 28-Jul-2023 15:57 (GMT)

10 ETHICS AND RESPONSIBILITIES

10.1 Good Clinical Practice

This study will be conducted in compliance with the protocol, Good Clinical Practice (GCP), Good Laboratory Practice (GLP), International Conference on Harmonization guidelines, and all applicable regulations, including the Federal Food, Drug and Cosmetic Act, U.S. applicable Code of Federal Regulations (title 21), any Independent Ethics Committee (IEC) requirements relative to clinical studies. The study will also be conducted in compliance with the recommendations laid down in the most recent version of the Declaration of Helsinki.

This study will be conducted in compliance with the protocol. The protocol and any amendments and the subject informed consent will receive Institutional Review Board/Independent Ethics Committee (IRB/IEC) approval/favorable opinion prior to initiation of the study.

The sponsor is responsible for ensuring that all updated relevant information related to the protocol be submitted to regulatory authorities and Independent Ethics Committees in accordance with local laws and regulations. This includes expedited reporting of suspected unexpected serious adverse reactions per regulatory guidelines.

All serious breaches must be reported to Pfizer (or designee) immediately. A Serious breach is a breach of the conditions and principles of GCP in connection with the study or protocol, which is likely to affect, to a significant degree, the safety or physical or mental integrity of the subjects of the study or the scientific value of the study. Examples include (but are not limited to):

- Missing, inadequate, or delinquent informed consent.
- Randomization of a subject that does not meet key eligibility criteria.
- Failure to withdraw a subject meeting discontinuation criteria.
- Unreported serious adverse events.
- Improper breaking of the blind.
- Conducting the study without IRB approval.
- Working under an expired medical license, or a debarred or disqualified status.
- Falsifying research or medical records.

Study personnel involved in conducting this study will be qualified by education, training, and experience to perform their respective task(s).

This study will not use the services of study personnel where sanctions have been invoked or where there has been scientific misconduct or fraud (e.g., loss of medical licensure, debarment).

It is the sponsor's responsibility to submit the protocol and its amendments (if any), and the ICFs to regulatory authorities when necessary.

10.2 Dissemination of Clinical Study Data

Pfizer fulfills its commitment to publicly disclose clinical study results through posting the results of studies on www.clinicaltrials.gov (ClinicalTrials.gov), the EudraCT/CTIS, and/or www.pfizer.com, and other public registries and websites in accordance with applicable local laws/regulations. In addition, Pfizer reports study results outside of the requirements of local laws/regulations pursuant to its SOPs.

In all cases, study results are reported by Pfizer in an objective, accurate, balanced, and complete manner and are reported regardless of the outcome of the study or the country in which the study was conducted.

www.clinicaltrials.gov

Pfizer posts clinical trial results on www.clinicaltrials.gov for Pfizer-sponsored interventional studies (conducted in patients) that evaluate the safety and/or efficacy of a product, regardless of the geographical location in which the study is conducted. These results are submitted for posting in accordance with the format and timelines set forth by US law.

EudraCT/CTIS

Pfizer posts clinical trial results on EudraCT/CTIS for Pfizer-sponsored interventional studies in accordance with the format and timelines set forth by EU requirements.

www.pfizer.com

Pfizer posts CSR synopses and plain-language study results summaries on www.pfizer.com for Pfizer-sponsored interventional studies at the same time the corresponding study results are posted to www.clinicaltrials.gov. CSR synopses will have personally identifiable information anonymized.

Documents within marketing applications

Pfizer complies with applicable local laws/regulations to publish clinical documents included in marketing applications. Clinical documents include summary documents and CSRs including the protocol and protocol amendments, sample CRFs, and SAPs. Clinical documents will have personally identifiable information anonymized.

Data sharing

090177e19e292258\Approved\Approved On: 28-Jul-2023 15:57 (GMT)

Pfizer provides researchers secure access to subject-level data or full CSRs for the purposes of “bona-fide scientific research” that contributes to the scientific understanding of the disease, target, or compound class. Pfizer will make data from these trials available 18 months after study completion. Subject-level data will be anonymized in accordance with applicable privacy laws and regulations. CSRs will have personally identifiable information anonymized.

Data requests are considered from qualified researchers with the appropriate competencies to perform the proposed analyses. Research teams must include a biostatistician. Data will not be provided to applicants with significant conflicts of interest, including individuals requesting access for commercial/competitive or legal purposes.

10.3 Institutional Review Board/Independent Ethics Committee

The Investigators agree to provide the IRB/IEC with all appropriate documents, including a copy of the protocol/amendments, ICFs, advertising text (if any), Investigator’s brochure (if any) and any other written information provided to study subjects. The trial will not begin until the Investigators have obtained the IRB/IEC favorable written approvals for the above-mentioned study documents.

In the event that the protocol is amended, the revised protocol must be approved by the IRB/IEC prior to its implementation, unless the changes involve only logistical or administrative aspects of the trial.

10.4 Informed Consent

Investigators must ensure that subjects, or, in those situations where consent cannot be given by subjects, their legally acceptable representatives, are clearly and fully informed about the purpose, potential risks, and other critical issues regarding clinical studies in which they volunteer to participate.

Pfizer (or designee) will provide the Investigator with an appropriate (i.e., Global or Local) sample informed consent form which will include all elements required by ICH, GCP and applicable regulatory requirements. The sample informed consent form will adhere to the ethical principles that have their origin in the Declaration of Helsinki.

Before the potential subject has undergone any study-related screening procedures, the nature of the study and the potential risks associated with it will be explained to the subject, and the subject will be given an opportunity to ask questions to his or her satisfaction. After the questions are answered, but before proceeding further, the subject must sign and date an IRB/IEC approved written informed consent form for study. The signed and dated ICF will be retained at the Investigator’s site, with a copy provided to the study subject and date will be entered in his or her CRF or appropriate system. The IRB/IEC must review and approve all protocol versions and informed consent form versions and a copy of each version of the IRB/IEC approved protocol and informed consent form is to be retained in the Study Master file. Any revisions to the protocol or ICF will be reviewed and approved by the IRB/IEC and subjects will

090177e19e292258\Approved\Approved On: 28-Jul-2023 15:57 (GMT)

be informed of ICF changes and document continuing consent by signing and dating the revised version of the ICF.

If a revised ICF is introduced during the study, each subject's further consent must be obtained. The new version of the ICF must be approved by the IRB/IEC, prior to subsequently obtaining each subject's consent.

If informed consent is initially given by a subject's legal guardian or legally acceptable representative, and the subject subsequently becomes capable of making and communicating their informed consent during the study, then the consent must additionally be obtained from the subject.

The informed consent form must also include a statement that Pfizer and its representatives and regulatory authorities may have direct access to subject records.

10.5 Case Report Forms

An Investigator is required to prepare and maintain adequate and accurate case histories designed to record all observations and other data pertinent to the investigation of each study patient. Data reported on the CRF that are derived from source documents must be consistent with the source documents or the discrepancies must be explained.

Electronic CRFs will be prepared for all data collection fields when EDC is being used.

The confidentiality of records that could identify patients must be protected, respecting the privacy and confidentiality rules in accordance with the applicable regulatory requirement(s).

The Investigator must retain a copy of the CRFs including records of changes and corrections. If EDC is being used, signatures will be obtained electronically and a copy of the electronic CRFs will be provided (or the data from the CRFs) for future reference.

10.6 Sponsor's Medically Qualified Individual

The contact information for the sponsor's MQI for the study is documented in the study contact list located in the Investigator Site File.

To facilitate access to their investigator and the sponsor's MQI for study related medical questions or problems from nonstudy healthcare professionals, subjects are provided with an ECC at the time of informed consent. The ECC contains, at a minimum, (a) protocol and study intervention identifiers, (b) subject's study identification number, (c) site emergency phone number active 24 hours/day, 7 days per week, and (d) Pfizer Call Center number.The ECC is intended to augment, not replace, the established communication pathways between the subject and their investigator and site staff, and between the investigator and sponsor study team. The ECC is only to be used by healthcare professionals not involved in the research study, as a means of reaching the investigator or site staff related to the care of a subject. The Pfizer Call Center number is to be used when the investigator and site staff are unavailable. The Pfizer Call Center

090177e19e292258\Approved\Approved On: 28-Jul-2023 15:57 (GMT)

number is not for use by the subject directly; if a subject calls that number directly, they will be directed back to the investigator site.

090177e19e292258\Approved\Approved On: 28-Jul-2023 15:57 (GMT)

11 RECORDS MANAGEMENT

In accordance with the principles of GCP and GLP, the study may be inspected by regulatory authorities, the sponsor and CRO. The sponsor is entitled to access information about the status of the study and to review the original documents of the study.

The Investigator must retain all study records and source documents for the maximum time period required by the applicable regulations and guidelines, or institution procedures or for the period of time specified by the sponsor, whichever is longer. The Investigator must contact the sponsor prior to destroying any records associated with this study.

Pfizer will notify the investigators when the study files for this study are no longer needed.

If the Investigator withdraws from the study (i.e., retirement, relocation), the records shall be transferred to a mutually agreed upon designee. Notice of such transfer will be given in writing to Pfizer.

It is the responsibility of the Investigator to ensure that the current disposition record of investigational product (may be supplied by the sponsor) is maintained at each study site where the study drug is inventoried and dispensed. Records or logs must comply with applicable regulations and guidelines and should include:

- amount of study drug received and placed in storage area
- label ID number or batch number or Kit number as specified for the protocol
- amount dispensed to and returned from each patient
- amount transferred to another area or site for dispensing or storage if applicable
- amount of drug lost or wasted
- amount destroyed at the site if applicable
- amount returned to the sponsor, if applicable
- retain samples for bioavailability/bioequivalence, if applicable
- record of dates and initials of personnel responsible for IP dispensing and accountability

11.1 Source Documentation

An Investigator is required to prepare and maintain adequate and accurate case histories designed to record all observations and other data pertinent for all subjects on study.

090177e19e292258\Approved\Approved On: 28-Jul-2023 15:57 (GMT)

If source documents are created to support the collection of study information, this must be retained with the other pertinent medical records for each subject for verification of data points, unless otherwise instructed by the sponsor or designee to enter data directly on the eCRF.

Study monitors will perform ongoing source data verification to confirm that data entered into the CRF by authorized site personnel are accurate, complete, and verifiable from source documents; that the safety and rights of subjects are being protected; and that the study is being conducted in accordance with the currently approved protocol and any other study agreements, ICH GCP, and all applicable regulatory requirements.

The Investigator must permit study-related monitoring, audits, IRB/IEC review, and regulatory agency inspections and provide direct access to source data documents.

11.2 Study Files and Record Retention

The sponsor does not require original documents that have already been scanned and entered into the eTMF system to be forwarded to the sponsor. Any original documents (i.e., 1572, signed financial disclosure, signed ICF, etc.) will be retained in the regulatory binder at the study site. The CRO will conduct a final TMF reconciliation to ensure all study files and regulatory documents have been correctly uploaded to the TMF prior to the close or termination of the study. Any materials or documents to support the clinical trial outside of the eTMF (i.e., rater training tapes) should be maintained by the CRO. The sponsor will be contacted to determine whether the study documents/materials that are retained outside of the TMF will be forwarded to the sponsor, destroyed or kept at the CRO or at another facility for a longer period of time at the sponsor's expense.

The CRO will maintain adequate study records after completion or termination of study. After that period, the sponsor will be contacted to determine whether the study records will be forwarded to the sponsor, destroyed or kept at CRO or at another facility for a longer period of time at the sponsor's expense.

090177e19e292258\Approved\Approved On: 28-Jul-2023 15:57 (GMT)

12 AMENDMENTS

Protocol modifications, except those intended to reduce immediate risk to study subjects, may be made only by Pfizer (or specified designee). A protocol change intended to eliminate an apparent immediate hazard to subjects may be implemented immediately.

Any permanent change to the protocol must be handled as a protocol amendment. The written amendment must be submitted to the IRB/IEC and the Investigator must await approval before implementing the changes. Pfizer or specified designee will submit protocol amendments to the appropriate regulatory authorities for approval.

If in the judgment of the IRB/IEC, the Investigator, and/or Pfizer, the amendment to the protocol substantially changes the study design and/or increases the potential risk to the subject and/or has an impact on the subject's involvement as a study subject, the currently approved written informed consent form will require similar modification. In such cases, informed consent will be renewed for subjects enrolled in the study before continued participation.

090177e19e292258\Approved\Approved On: 28-Jul-2023 15:57 (GMT)

13 PUBLICATION POLICY

The publication policy of Pfizer is discussed in the Investigator's Clinical Research Agreement.

090177e19e292258\Approved\Approved On: 28-Jul-2023 15:57 (GMT)

14 STUDY DISCONTINUATION

Both Pfizer and the Principal Investigator reserve the right to terminate the study at the Investigator's site at any time. Should this be necessary, Pfizer or a specified designee will inform the appropriate regulatory authorities of the termination of the study and the reasons for its termination, and the Principal Investigator will inform the IRB/IEC of the same. In terminating the study, Pfizer and the Principal Investigator will assure that adequate consideration is given to the protection of the subjects' interests.

090177e19e292258\Approved\Approved On: 28-Jul-2023 15:57 (GMT)

15 DATA PROTECTION

All parties will comply with all applicable laws, including laws regarding the implementation of organizational and technical measures to ensure protection of subject data.

Subjects' personal data will be stored at the study site in encrypted electronic and/or paper form and will be password protected or secured in a locked room to ensure that only authorized study staff have access. The study site will implement appropriate technical and organizational measures to ensure that the personal data can be recovered in the event of disaster. In the event of a potential personal data breach, the study site will be responsible for determining whether a personal data breach has in fact occurred and, if so, providing breach notifications as required by law.

To protect the rights and freedoms of subjects with regard to the processing of personal data, subjects will be assigned a single, subject-specific numerical code. Any subject records or data sets that are transferred to the sponsor will contain the numerical code; subject names will not be transferred. All other identifiable data transferred to the sponsor will be identified by this single, subject-specific code. The study site will maintain a confidential list of subjects who participated in the study, linking each subject's numerical code to their actual identity and medical record ID. In case of data transfer, the sponsor will protect the confidentiality of subjects' personal data consistent with the clinical study agreement and applicable privacy laws.

Information technology systems used to collect, process, and store study-related data are secured by technical and organizational security measures designed to protect such data against accidental or unlawful loss, alteration, or unauthorized disclosure or access.

The sponsor maintains SOPs on how to respond in the event of unauthorized access, use, or disclosure of sponsor information or systems.

When subject data are to be deleted, the investigator will ensure that all copies of such data are promptly and irrevocably deleted from all systems.

090177e19e292258\Approved\Approved On: 28-Jul-2023 15:57 (GMT)

16 APPENDICES

16.1 APPENDIX I: Strong Inhibitors and Strong and Moderate Inducers of CYP3A4 Protein (Not all-inclusive)

The following medications and medication combinations are some of the strong inhibitors of CYP3A4). This list should not be considered all-inclusive. As described in the study protocol, concomitant use of strong CYP3A4 inhibitors is prohibited. Individual drug labels should be reviewed for specific information on propensity to inhibit CYP3A4 enzymes for a specific compound.

**Strong CYP3A4 inhibitors**
boceprevir, cobicistat, conivaptan, danoprevir and ritonavir, elvitegravir and ritonavir, indinavir and ritonavir, itraconazole, ketoconazole, lopinavir and ritonavir, paritaprevir and ritonavir and (ombitasvir and/or dasabuvir), posaconazole, ritonavir, saquinavir and ritonavir, telaprevir, tipranavir and ritonavir, troleandomycin, voriconazole, clarithromycin, nefazodone, nelfinavir, mifepristone, mibefradil.

The following medications and supplements are moderate to strong inducers of CYP3A4. As described in the study protocol, concomitant use of moderate to strong CYP3A4 inducers is prohibited. This list should not be considered all-inclusive. Individual product labels should be reviewed for specific information on propensity to cause moderate to strong induction of the CYP3A4 enzyme for a specific compound.

**Strong CYP3A4 inducers**
apalutamide, carbamazepine, phenobarbital, phenytoin, rifampin, rifapentine, St. John's Wort

**Moderate CYP3A4 inducers**
Bosentan, rifabutin, modafinil, nafcillin, efavirenz, etravirine, lopinavir

Resources:

https://www.fda.gov/Drugs/DevelopmentApprovalProcess/DevelopmentResources/DrugInteractionsLabeling/ucm093664.htm#table3-2

https://www.fda.gov/Drugs/DevelopmentApprovalProcess/DevelopmentResources/DrugInteractionsLabeling/ucm093664.htm#table3-3

090177e19e292258\Approved\Approved On: 28-Jul-2023 15:57 (GMT)

Hachad H, Ragueneau-Majlessi I, Levy RH. A useful tool for drug interaction evaluation: the University of Washington Metabolism and Transport Drug Interaction Database. Hum Genomics. 2010 Oct;5(1):61-72.

University of Washington Metabolism and Transport Drug Interaction Database accessible at https://www.druginteractioninfo.org/

090177e19e292258\Approved\Approved On: 28-Jul-2023 15:57 (GMT)

16.2 APPENDIX II: ECG Findings of Potential Clinical Concern

ECG Findings That May Qualify as AEs
• Marked sinus bradycardia (rate <40 bpm) lasting minutes.<br>• New PR interval prolongation >280 ms.<br>• New prolongation of QTcF to >480 ms (absolute).<br>• New prolongation of QTcF by >60 ms from baseline.<br>• New-onset atrial flutter or fibrillation, with controlled ventricular response rate: ie, rate <120 bpm.<br>• New-onset type I second-degree (Wenckebach) AV block of >30-second duration.<br>• Frequent PVCs, triplets, or short intervals (<30 seconds) of consecutive ventricular complexes.
**ECG Findings That May Qualify as SAEs**
• QTcF prolongation >500 ms.<br>• Absolute value of QTcF >450 ms AND QTcF change from baseline >60 ms.<br>• New ST-T changes suggestive of myocardial ischemia.<br>• New-onset LBBB (QRS complex >120 ms).<br>• New-onset right bundle branch block (QRS complex >120 ms).<br>• Symptomatic bradycardia.<br>• Asystole<br>○ In awake, symptom-free subjects in sinus rhythm, with documented asystolic pauses ≥3 seconds or any escape rate <40 bpm, or with an escape rhythm that is below the AV node;<br>○ In awake, symptom-free subjects with atrial fibrillation and bradycardia with 1 or more asystolic pauses of at least 5 seconds or longer.<br>• Atrial flutter or fibrillation, with rapid ventricular response rate: rapid = rate >120 bpm.<br>• Sustained supraventricular tachycardia (rate >120 bpm) ("sustained" = short duration with relevant symptoms or lasting >1 minute).<br>• Ventricular rhythms >30 second duration, including idioventricular rhythm (HR <40 bpm), accelerated idioventricular rhythm (HR 40 bpm to <100 bpm), and monomorphic/polymorphic ventricular tachycardia (HR >100 bpm [such as torsades de pointes]).<br>• Type II second-degree (Mobitz II) AV block.<br>• Complete (third-degree) heart block.
**ECG Findings That Qualify as SAEs**
• Change in pattern suggestive of new myocardial infarction.<br>• Sustained ventricular tachyarrhythmias (>30-second duration).<br>• Second- or third-degree AV block requiring pacemaker placement.<br>• Asystolic pauses requiring pacemaker placement.<br>• Atrial flutter or fibrillation with rapid ventricular response requiring cardioversion.

090177e19e292258\Approved\Approved On: 28-Jul-2023 15:57 (GMT)

- Ventricular fibrillation/flutter.
- At the discretion of the investigator, any arrhythmia classified as an adverse experience.

The major events of potential clinical concern listed above are recommended as "alerts" or notifications from the core ECG laboratory to the investigator and Pfizer study team, and not to be considered as all-inclusive of what is to be reported as AEs/SAEs.

090177e19e292258\Approved\Approved On: 28-Jul-2023 15:57 (GMT)

16.3 APPENDIX III: Contraceptive and Barrier Guidance

16.3.1 Male Subject Reproductive Inclusion Criteria

No contraception methods are required for male subjects in this study, as the calculated safety margin is ≥100 fold between the estimated maternal exposure due to seminal transfer and the NOAEL for serious manifestations of developmental toxicity in nonclinical studies.

16.3.2 Female Subject Reproductive Inclusion Criteria

The criteria below are part of Inclusion Criterion 2.b (Age and Sex; Section 5.2) and specify the reproductive requirements for including female subjects. Refer to Section 16.3.4 for a complete list of contraceptive methods permitted in the study.

A female subject is eligible to participate if she is not pregnant or breastfeeding and at least 1 of the following conditions applies:

- Is not a WOCBP (see definitions below in Section 16.3.3).

OR

- Is a WOCBP and agrees to use an acceptable contraceptive method during the intervention period (for a minimum of 28 days after the last dose of study intervention). The investigator should evaluate the effectiveness of the contraceptive method in relationship to the first dose of study intervention.

The investigator is responsible for review of medical history, menstrual history, and recent sexual activity to decrease the risk for inclusion of a woman with an early undetected pregnancy.

16.3.3 Woman of Childbearing Potential

A woman is considered fertile following menarche and until becoming postmenopausal unless permanently sterile (see below).

If fertility is unclear (eg, amenorrhea or oligomenorrhea) and a menstrual cycle cannot be confirmed before the first dose of study intervention, additional evaluation should be considered.

Women in the following categories are not considered WOCBP:

1. Premenarchal.
2. Premenopausal female with 1 of the following:
    - Documented hysterectomy
    - Documented bilateral salpingectomy

090177e19e292258\Approved\Approved On: 28-Jul-2023 15:57 (GMT)

- Documented bilateral oophorectomy

For individuals with permanent infertility due to a medical cause other than the above (eg, mullerian agenesis, androgen insensitivity), investigator discretion should be applied to determining study entry.

Note: Documentation for any of the above categories can come from the site personnel's review of the subject's medical records, medical examination, or medical history interview. The method of documentation should be recorded in the subject's medical record for the study.

3. Postmenopausal female:

- A postmenopausal state is defined as no menses for 12 months without an alternative medical cause. In addition:
    - A high FSH level in the postmenopausal range must be used to confirm a postmenopausal state in women under 60 years of age and not using hormonal contraception or HRT.
    - A female on HRT and whose menopausal status is in doubt will be required to use one of the highly effective nonestrogen hormonal contraception methods if she wishes to continue her HRT during the study. Otherwise, she must discontinue HRT to allow confirmation of postmenopausal status before study enrollment.

16.3.4 Contraception Methods

Contraceptive use by men or women should be consistent with local availability/regulations regarding the use of contraceptive methods for those participating in clinical trials.

The following contraceptive methods are appropriate for this study:

<u>Highly Effective Methods That Have Low User Dependency</u>

1. Implantable progestogen-only hormone contraception associated with inhibition of ovulation.
2. Intrauterine device.
3. Intrauterine hormone-releasing system.
4. Bilateral tubal occlusion.
5. Vasectomized partner:
    - Vasectomized partner is a highly effective contraceptive method provided that the partner is the sole sexual partner of the WOCBP and the absence of sperm has been

090177e19e292258\Approved\Approved On: 28-Jul-2023 15:57 (GMT)

confirmed. If not, an additional highly effective method of contraception should be used. The spermatogenesis cycle is approximately 90 days.

Highly Effective Methods That Are User Dependent

6. Combined (estrogen- and progestogen-containing) hormonal contraception associated with inhibition of ovulation:
   - Oral
   - Intravaginal
   - Transdermal
7. Progestogen-only hormone contraception associated with inhibition of ovulation:
   - Oral
   - Injectable
8. Sexual Abstinence
   - Sexual abstinence is considered a highly effective method only if defined as refraining from heterosexual intercourse during the entire period of risk associated with the study intervention. The reliability of sexual abstinence needs to be evaluated in relation to the duration of the study and the preferred and usual lifestyle of the subject.

Other Effective Methods

9. Progestogen-only oral hormonal contraception where inhibition of ovulation is not the primary mode of action.
10. Male or female condom, with or without spermicide.
11. Cervical cap, diaphragm, or sponge with spermicide.
12. A combination of male condom with either cervical cap, diaphragm, or sponge with spermicide (double-barrier methods).

090177e19e292258\Approved\Approved On: 28-Jul-2023 15:57 (GMT)

090177e19e292258\Approved\Approved On: 28-Jul-2023 15:57 (GMT)

16.4 APPENDIX IV: Protocol Amendment History

The protocol amendment summary of changes table for the current amendment is located directly before the Study summary. The protocol amendment summary of changes tables for past amendment(s) can be found below:

Version Number	Brief Description Summary of Changes	Date
Version 1.0 – Original	Not Applicable	21 Oct 2021
Version 2.0 – Amendment 1	Corrected the requirement: Subjects must have had at least two instances of facial pain/pressure/fullness that had reached a current intensity of moderate or severe on a 4-point rating scale (0 = None, 1 = Mild, 2 = Moderate, 3 = Severe) within 30 days prior to the Screening Visit. This was corrected in two places that it appears in the protocol by adding "or severe" following the word "moderate". This was updated in the synopsis and in Inclusion 1c.<br><br>Updated the power calculation/sample size in Section 9.2 to increase the number of evaluable subjects from 160 to 200.<br><br>Primary, secondary, and exploratory objectives were updated to ensure consistency in the way they are written in the synopsis and body of the protocol.<br><br>The word "acute" was inserted into the description of the study design and primary objective to provide clarity. The study design now reads: This is a double-blind, randomized, multicenter, outpatient evaluation of the safety and efficacy of rimegepant as compared to placebo for the acute treatment of chronic rhinosinusitis (CRS) with or without nasal polyps.<br><br>Following feedback from the FDA, the following sentence was added to the protocol: Subjects should have access to systemic corticosteroids and antibiotics during the trial for treatment of symptoms, if indicated. However, subjects should discontinue the study if they receive those therapies. This was added to the protocol synopsis, Section 4.2.2.1, and Section 5.5.<br><br>The text "stable dosing, not PRN" was added to Section 5.5 for Leukotriene modifiers and Cromolyn.<br><br>The study schematic was updated to be more concise to the study and for formatting purposes. References to pain were changed to pain/pressure/fullness.<br><br>In Section 6.1, Electronic CRF Instructions were removed from the list of study materials. CRF instructions are embedded in the eCRF.<br><br>Weekly contact (e.g., phone, text, email) was added to the study between the Baseline Visit and dosing to monitor (and remind subjects	21 Jan 2021

Version Number	Brief Description Summary of Changes	Date
	to report) any systemic corticosteroid and/or antibiotics (other than topical nasal antibiotics) use. This was added to Table 1 BHV3000-316 Schedule of Assessments and Section 6.2.6.<br><br>FSH, HbA1c and Lipid panel were added to Table 1 BHV3000-316 Schedule of Assessments and Section 6.2.4.1.<br><br>Table 1 BHV3000-316 Schedule of Assessments was updated to clarify and correct the comments for the C-SSRS and the SNOT-22 procedures.<br><br>The following exclusion criteria were updated to either correct or clarify what was written or to add consistency with other aspects of the protocol: 1f, 4a, 6a, and 6i.<br><br>In Section 5.4 Prohibited and Restricted Concomitant Medications was updated. Number 3 in the list describing systemic corticosteroids was updated and a description of prohibited antibiotics was added to be consistent with the FDA's feedback.<br><br>In Section 6.4 Early Discontinuation from the Study, use of systemic corticosteroids and use of antibiotics (other than topical nasal antibiotics) were added to the list of reasons a subject must discontinue the study.<br><br>In section 8.2.1 Collection and Reporting of Non-Serious Adverse Events, the sentence "Non serious AE information should also be collected from the start of a placebo lead-in phase or other observation period intended to establish a baseline status for a subject."	
Version 3.0 – Amendment 2	Topical nasal anticholinergics were added to the list of allowed rescue medications and medications that may be taken for non-qualifying CRS associated facial pain/pressure/fullness. This has been added in the Study Design section of the synopsis; Section 4.2.2.1, and Section 5.5.<br><br>Section 5.2, Inclusion 2.b. was further clarified with additional text: Original text: Radiographic imaging showing inflammation of the paranasal sinuses. New Text: Radiographic imaging showing inflammation of the paranasal sinuses *with partial opacification of at least two sinuses or complete opacification of at least one sinus*.<br><br>Section 5.2, Inclusion 2.d. was updated to clarify requirements for daily use of medications. Original Text: Subject with daily use of medications to treat *CRS symptoms (please refer to Section 5.5)* is permitted to remain on therapy if they have been on a stable dose for at least *3 months* prior to the Screening Visit, and the dose is not expected to change during the course of the study. New Text: Subject with daily use of medications *listed in Section 5.5* is permitted to remain on therapy if they have been on a stable dose for at least *1*	15 Mar 2022

090177e19e292258\Approved\Approved On: 28-Jul-2023 15:57 (GMT)

Version Number	Brief Description Summary of Changes	Date
	month prior to the Screening Visit, and the dose is not expected to change during the course of the study.<br><br>Section 5.3, Exclusion 1a was updated to clarify that infrequent tension-type headache is not exclusionary. The following sentence was added: Infrequent tension-type headache consistent with diagnostic criteria according to the ICHD-3 is not exclusionary.<br><br>Section 5.3, Exclusion 1e., n., o., and p. were updated to clarify exclusionary concomitant illnesses:<br><br>1.e. was changed from Subject has *diagnosed or suspected* invasive fungal rhinosinusitis. to Subject has *a history* of invasive fungal rhinosinusitis.<br><br>1.n. was changed from Subject has a *diagnosis* of sinus mucocele. to Subject has a *history of* sinus mucocele *within 1 year prior to the Screening Visit.*<br><br>1.o. was changed from Subject has a *diagnosis* of nasal or paranasal tumor. to Subject has a *history* of *malignant* nasal or paranasal tumor.<br><br>1.p. was changed from Subject has a *diagnosis* of odontogenic sinusitis. to Subject has a *history* of odontogenic sinusitis *within 1 year prior to the Screening Visit.*<br><br>Section 5.3 Exclusion 2.d. clarifies the definition of uncontrolled hypertension. The following sentence was added: *A single blood pressure measurement of greater than 150 mmHg systolic or 100 mmHg diastolic after 10 minutes of rest is exclusionary.*<br><br>Section 5.3 Exclusion 2.g. was updated to add that subjects with severe heptic impairment (Child-Pugh C) are to be excluded. The following sentence was added to Exclusion 2.g: *Severe hepatic impairment (Child-Pugh C) is exclusionary in all cases.*<br><br>Section 5.3 Exclusion 6.g. updated the history of new or recently modified leukotriene modifiers therapy. Text was changed from Subject has a history of new or recently modified leukotriene modifiers therapy within the *3 months* prior to Screening Visit. to Subject has a history of new or recently modified leukotriene modifiers therapy within the *1 month* prior to Screening Visit.<br><br>Section 5.3 Exclusion 6.h. updated the history of new or recently modified leukotriene modifiers therapy. Text was changed from Subject has a history of new or recently modified β-adrenoceptor agonists therapy within the *3 months* prior to Screening Visit. to Subject has a history of new or recently modified β-adrenoceptor agonists therapy within the *1 month* prior to Screening Visit.<br><br>Section 5.4 Prohibited and Restricted Concomitant Medications: #16 was added to read *Topical nasal decongestants should not be taken*	

090177e19e292258\Approved\Approved On: 28-Jul-2023 15:57 (GMT)

Version Number	Brief Description Summary of Changes	Date
	more than 3 times per week.<br><br>Section 5.4 Prohibited and Restricted Concomitant Medications: Numbers 3, 4 and 5 were updated to be restricted prior to the Screening Visit. Formerly they were restricted prior to the Baseline Visit. See text below.<br><br>3. Systemic corticosteroids (e.g., prednisone, prednisolone, methylprednisolone, betamethasone, dexamethasone, triamcinolone, hydrocortisone, cortisone) should not be taken 30 days prior to the *Screening* Visit and throughout the study. Subjects should have access to systemic corticosteroids during the trial for treatment of symptoms, if indicated. However, subjects should discontinue the study if they receive this therapy.<br><br>4. Antibiotics, other than topical nasal antibiotics, should not be taken 14 days prior to the *Screening* Visit and throughout the study. Subjects should have access to antibiotics during the trial for treatment of symptoms, if indicated. However, subjects should discontinue the study if they receive this therapy.<br><br>5. Monoclonal antibody therapy (e.g., dupilumab, mepolizumab, omalizumab, benralizumab, reslizumab) should not be taken 6 months prior to the *Screening* Visit and throughout the study.<br><br>Section 5.4: Paragraph was updated to further clarify the criteria for daily use medications. Text was updated from Subjects with daily use of medications *to treat CRS symptoms* are permitted to remain on therapy provided they have been on a stable dose for at least *3 months* prior to the *Baseline* Visit *(see Section 5.5).* to Subjects with daily use of medications *listed in Section 5.5* are permitted to remain on therapy provided they have been on a stable dose for at least *1 month* prior to the *Screening* Visit.<br><br>Section 5.5 Daily and Rescue Medications was updated to reduce the time medications must be stable and to clarify that saline, as specified in the new text, may be used with up to two topical nasal medications. In addition, the list of medications was updated and clarified.<br><br>Section was updated from: *Subjects may not use more than 1 of the following medications*. Medication doses must be **stable within** ***3 months*** **prior to the Baseline Visit** and throughout the study.<br><br>*<u>Daily medications that are permitted during the study include:</u>*<br><br>• *<u>Daily medications that are permitted during the study include:</u>*<br><br>• *Topical nasal corticosteroids (spray or rinse; stable dosing, not PRN)*<br><br>• *Topical nasal antibiotics and antifungal medications (spray or*	

090177e19e292258\Approved\Approved On: 28-Jul-2023 15:57 (GMT)

090177e19e292258\Approved\Approved On: 28-Jul-2023 15:57 (GMT)

Version Number	Brief Description Summary of Changes	Date
	rinse; stable dosing, not PRN)<br><br>• *Topical nasal anticholinergics (spray or rinse; stable dosing, not PRN)*<br><br>• *Topical and oral antihistamines (non-sedating; stable dosing, not PRN)*<br><br>• *Saline (spray or rinse, additives such as detergents or moisturizers are ok)*<br><br>• *Oral decongestants (stable dosing, not PRN)*<br><br>• *Leukotriene modifiers (stable dosing, not PRN)*<br><br>• *Cromolyn (stable dosing, not PRN)*<br><br>• *Mucolytics (e.g., acetylcysteine) (stable dosing, not PRN)*<br><br>After dosing with study medication, all other *pain* medication is prohibited during the 2 hours post-dose. At the end of 2 hours after dosing with study medication (and after the 2-hour assessments have been completed on the eDiary), subjects will be permitted to use the following rescue medication: acetaminophen (up to 1000 mg/day), or aspirin, ibuprofen, naproxen (or any other type of non-steroidal anti-inflammatory drug (NSAID)), oral antihistamines (non-sedating), oral decongestants, topical nasal decongestants (all up to the daily recommended dose indicated on the drug packaging). These are the only medications allowed for rescue treatment after 2 hours post dose of study medication.<br><br>To: Medication doses must be **stable within *one month* prior to the *Screening*** Visit and throughout the study. <u>Subjects may use:</u><br><br>• *Saline and other non-medicated spray/rinse (additives such as detergents, moisturizers, mucolytics are permitted) - <u>maintain dosing regimen established prior to the Screening Visit</u>*<br><br>*AND <u>up to two of the topical</u>* ***nasal medications (stable dosing, not PRN, any indication):***<br><br>• *Topical nasal corticosteroids (spray or rinse)*<br><br>• *Topical nasal antibiotics and antifungal medications (spray or rinse)*<br><br>• *Topical nasal antihistamines (non-sedating)*<br><br>• *Topical nasal cromolyn*<br><br>*AND any of the following medications (**stable dosing, not PRN**):*	

Version Number	Brief Description Summary of Changes	Date
	• *Leukotriene modifiers*<br>• *Cromolyn (oral or inhalation)*<br>• *Methylxantines (e.g., theophylline, aminophyllines)*<br>• *Mucoactive agents (e.g., guaifenesin, acetylcysteine)*<br><br>*Subjects with concomitant asthma or COPD may remain on their baseline SoC treatment if they have been on a <u>stable dose for at least 1 month</u> prior to the Screening Visit and if not specifically listed in Section 5.4 Prohibited and Restricted Concomitant Medications, and the <u>dose is not expected to change</u> during the course of the study. Subjects may use their rescue inhalers and/or nebulizers as directed by their HCP.*<br><br>**After dosing with study medication, all other *CRS* medication is prohibited during the 2 hours post-dose.** At the end of 2 hours after dosing with study medication (and after the 2-hour assessments have been completed on the eDiary), subjects will be permitted to use the following rescue medication: acetaminophen (up to 1000 mg/day), or aspirin, ibuprofen, naproxen (or any other type of non-steroidal anti-inflammatory drug (NSAID)), oral antihistamines (non-sedating), oral decongestants, topical nasal decongestants, *topical nasal anticholinergics* (all up to the daily recommended dose indicated on the drug packaging). These are the only medications allowed for rescue treatment after 2 hours post dose of study medication.<br><br>Section 8.4 Pregnancy: This section instructing where to email the Pregnancy Form has a typographical error. BHV3000-314 was updated to BHV3000-316.	

090177e19e292258\Approved\Approved On: 28-Jul-2023 15:57 (GMT)

17 REFERENCES

1. Rosenfeld RM, Piccirillo JF, Chandrasekhar SS, et al. Clinical practice guideline (update): adult sinusitis. Otolaryngol Head Neck Surg 2015;152:S1-S39.

2. Bhattacharyya N, Orlandi RR, Grebner J, Martinson M. Cost burden of chronic rhinosinusitis: a claims-based study. Otolaryngol Head Neck Surg 2011;144:440-5.

3. Sedaghat AR. Chronic Rhinosinusitis. Am Fam Physician 2017;96:500-6.

4. Bachert C, Gevaert P, Hellings P. Biotherapeutics in Chronic Rhinosinusitis with and without Nasal Polyps. J Allergy Clin Immunol Pract 2017;5:1512-6.

5. Shah SA, Ishinaga H, Takeuchi K. Pathogenesis of eosinophilic chronic rhinosinusitis. J Inflamm (Lond) 2016;13:11.

6. Hamilos DL. Chronic rhinosinusitis: epidemiology and medical management. J Allergy Clin Immunol 2011;128:693-707; quiz 8-9.

7. Fokkens WJ, Lund VJ, Hopkins C, et al. European Position Paper on Rhinosinusitis and Nasal Polyps 2020. Rhinology 2020;58:1-464.

8. Suh JD, Kennedy DW. Treatment options for chronic rhinosinusitis. Proc Am Thorac Soc 2011;8:132-40.

9. Stevens WW, Schleimer RP. Aspirin-Exacerbated Respiratory Disease as an Endotype of Chronic Rhinosinusitis. Immunol Allergy Clin North Am 2016;36:669-80.

10. Bonfils P, Halimi P, Malinvaud D. Adrenal suppression and osteoporosis after treatment of nasal polyposis. Acta Otolaryngol 2006;126:1195-200.

11. Sato I, Imura K, Miwa Y, Yoshida S, Sunohara M. Distributions of calcitonin gene-related peptide and substance P in the human maxillary sinus of Japanese cadavers. J Craniomaxillofac Surg 2012;40:e249-52.

12. Sato T, Sasahara N, Kanda N, et al. Distribution of CGRP and TRPV2 in Human Paranasal Sinuses. Cells Tissues Organs 2017;203:55-64.

13. Rangi SP, Sample S, Serwonska MH, Lenahan GA, Goetzl EJ. Mediation of prolonged increases in nasal mucosal blood flow by calcitonin gene-related peptide (CGRP). J Clin Immunol 1990;10:304-10.

14. Baraniuk JN, Kaliner MA. Neuropeptides and nasal secretion. J Allergy Clin Immunol 1990;86:620-7.

15. Baraniuk JN, Lundgren JD, Goff J, et al. Calcitonin gene-related peptide in human nasal mucosa. Am J Physiol 1990;258:L81-8.

090177e19e292258\Approved\Approved On: 28-Jul-2023 15:57 (GMT)

16. Brokaw JJ, White GW. Calcitonin gene-related peptide potentiates substance P-induced plasma extravasation in the rat trachea. Lung 1992;170:85-93.

17. Cambridge H, Brain SD. Calcitonin gene-related peptide increases blood flow and potentiates plasma protein extravasation in the rat knee joint. Br J Pharmacol 1992;106:746-50.

18. Guarnaccia S, Baraniuk JN, Bellanti J, Duina M. Calcitonin gene-related peptide nasal provocation in humans. Ann Allergy 1994;72:515-9.

19. Lacroix JS, Kurt AM, Pochon N, Bretton C, Lundberg JM, Deshusses J. Neutral endopeptidase activity and concentration of sensory neuropeptide in the human nasal mucosa. Eur Arch Otorhinolaryngol 1995;252:465-8.

20. Bellamy JL, Cady RK, Durham PL. Salivary levels of CGRP and VIP in rhinosinusitis and migraine patients. Headache 2006;46:24-33.

21. Zheng C, Wang Z, Lacroix JS. Effect of intranasal treatment with capsaicin on the recurrence of polyps after polypectomy and ethmoidectomy. Acta Otolaryngol 2000;120:62-6.

22. Malis DD, Rist B, Nicoucar K, Beck-Sickinger AG, Morel DR, Lacroix JS. Modulatory effect of two novel CGRP receptor antagonists on nasal vasodilatatory responses to exogenous CGRP, capsaicin, bradykinin and histamine in anaesthetised pigs. Regul Pept 2001;101:101-8.

23. Rinder J, Lundberg JM. Effects of hCGRP 8-37 and the NK1-receptor antagonist SR 140.333 on capsaicin-evoked vasodilation in the pig nasal mucosa in vivo. Acta Physiol Scand 1996;156:115-22.

24. APA. Diagnostic and Statistical Manual of Mental Disorders, 5th Edition: DSM-5 5th Edition. 5th ed: American Psychiatric Publishing; 2013.

25. Posner K, Brown GK, Stanley B, et al. The Columbia-Suicide Severity Rating Scale: initial validity and internal consistency findings from three multisite studies with adolescents and adults. Am J Psychiatry 2011;168:1266-77.

26. Posner K, Brent D, Lucas C, et al. Columbia-Suicide Severity Rating Scale. The Research Foundation for Mental Hygiene, inc 2009;Version 1/14/09.

27. Salaffi F, Stancati A, Silvestri CA, Ciapetti A, Grassi W. Minimal clinically important changes in chronic musculoskeletal pain intensity measured on a numerical rating scale. Eur J Pain 2004;8:283-91.

28. Banholzer ML, Wandel C, Barrow P, et al. Clinical trial considerations on male contraception and collection of pregnancy information from female partner: update. Clin Transl Med. 2016;5(1)(Dec): 1-14.

090177e19e292258\Approved\Approved On: 28-Jul-2023 15:57 (GMT)

Document Approval Record

**Document Name:**	PF-07899801_Protocol Amendment_3_C4951015_FINAL_clean_26Jul 2023
**Document Title:**	PF-07899801_Protocol Amendment_3_C4951015_FINAL_clean_26Jul 2023

**Signed By:**	**Date(GMT)**	**Signing Capacity**
PPD	28-Jul-2023 13:33:26	Final Approval
PPD	28-Jul-2023 15:57:39	Final Approval

090177e19e292258\Approved\Approved On: 28-Jul-2023 15:57 (GMT)
